# A molecular systems architecture of asthma

**DOI:** 10.3389/fimmu.2026.1788984

**Published:** 2026-04-24

**Authors:** V. A. Shiva Ayyadurai, Prabhakar Deonikar, Yamuna Manoharan

**Affiliations:** 1 Systems Biology Group, CytoSolve Research Division, CytoSolve, Inc., Cambridge, MA, United States; 2 Open Science Institute, International Center for Integrative Systems, Cambridge, MA, United States

**Keywords:** airway inflammation, airway remodeling, asthma, CytoSolve^®^, hyperresponsiveness, pathogenesis, psychological stress, systems biology

## Abstract

**Background:**

Asthma is a heterogeneous inflammatory disease driven by complex genetic, immunological, environmental, and neuro-immune interactions. Modern therapeutic strategies increasingly target distinct molecular mechanisms underlying specific asthma endotypes. Emerging evidence highlights the role of psychological stress in modulating the neuro-immune axis, contributing to allergic airway inflammation. Systems biology offers a powerful framework to understand the multi-cellular and cross-organ interactions between lung and brain microenvironments that drive asthma pathogenesis.

**Objective:**

To develop a molecular systems architecture of asthma using the CytoSolve^®^ systems biology platform and process. This approach enables a multi-layered, systems-level analysis of molecular pathway interactions across thirty-one pulmonary, immune, and neuronal cell types involved in allergic-eosinophilic and non-allergic asthma phenotypes, and identifies potential therapeutic targets.

**Methods:**

A systematic bioinformatics literature review was conducted using Medical Subject Headings (MeSH) across PubMed, Medline, and Google Scholar, covering peer-reviewed publications from January 2008 to August 2025. Relevant full-length articles were curated and analyzed using the CytoSolve^®^ platform to construct a molecular systems architecture of asthma. The relevant literature was critically analyzed to understand the link between environmental and psychological stress triggers that drive asthma pathogenesis and disease exacerbations.

**Result:**

The systems architecture identified biomolecular interactions across thirty-one cell types spanning bronchial, immune, stromal, vascular, endocrine, and neuronal compartments, including airway epithelial cells, T-cells, eosinophils, mast cells, fibroblasts, microglia, hypothalamic and brainstem neurons, vagal sensory neurons, and autonomic airway neurons. Environmental triggers such as pollutants and infections initiate cascades that promote three core pathobiological processes: airway inflammation, hyperresponsiveness, and remodeling. Psychological comorbidities, including anxiety and depression, further amplify airway inflammation through brain-lung cross-talk, contributing to neuronal inflammation and asthma exacerbations.

**Conclusions:**

This system architecture generated a multilayered visual map that shows the associations between various triggers and biomolecular interactions across airway and neuronal cell types in the lung and brain microenvironment, respectively. The architecture may be utilized for target identification, discovery of single and combination therapeutics, biomarkers, and clinical strategies to treat asthma endotypes.

## 1 Introduction

Nearly 213,795 research studies on asthma have been carried out in the last 50 years (1). Asthma is a complex systemic disease, and these research studies have used the reductionist approach to understand the components of asthma pathogenesis. This study aims to inspire the field to embrace systems biology approaches to advance asthma research. Specifically, this study hypothesizes that the development of a molecular systems architecture of the airway microenvironment in asthma may: 1) enable the visualization of complex biomolecular systems interactions across the airway microenvironment involving thirty-one cell types; 2) reveal the biological processes and the underlying molecular pathways leading to disease pathogenesis; 3) identify potential targets for treatment; and, 4) provide a framework to develop predictive and quantitative models for drug development.

Systems biology elucidates the diverse types of high-dimensional interactions among molecular signaling networks, leading to cellular interactions that affect the biological processes associated with system physiology (2). The current drug development paradigm, focused primarily on reductionist approaches, has several drawbacks. These comprise the inability to develop combination therapies that produce curative effects, the lack of “one-size-fits-all” personalization, the inability to integrate the complexity of molecular interactions, and the lack of a robust computational framework to predict systems-level adverse effects (3, 4). Since system biology has improved our understanding of the complex molecular mechanisms in disease, it is hypothesized that a systems biology approach may offer a new paradigm for drug development, as it addresses these weaknesses (5).

**Figure 1:**
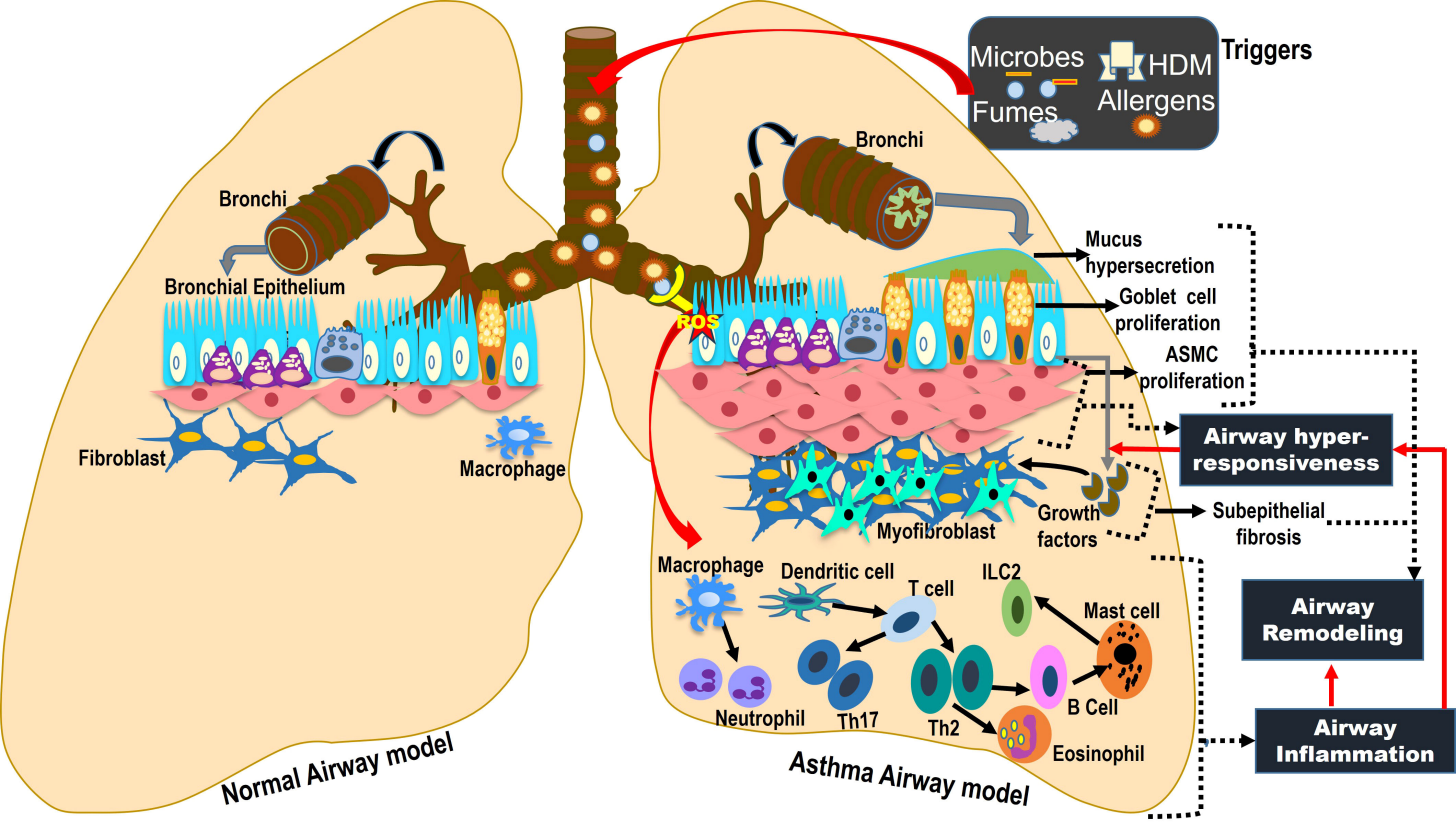
The Asthma Lung Microenvironment (ALM). ALM shows the onset of allergic airway inflammation in asthma. Environmental triggers generate ROS in conducting airways, damaging the bronchial epithelium, initiating the activation of lung resident alveolar macrophages and influx of dendritic cells and CD4+ T cells, promoting the cascade of T helper cell polarizations like Th2 and Th17 that contribute to both eosinophilic and neutrophilic airway inflammation. The cytokines and growth factors released by inflammatory cells and damaged epithelial cells, respectively, interact with airway structural cells like airway smooth muscle cells, goblet epithelial cells, and fibroblasts, causing airway remodeling and hyperresponsiveness.

**Figure 2:**
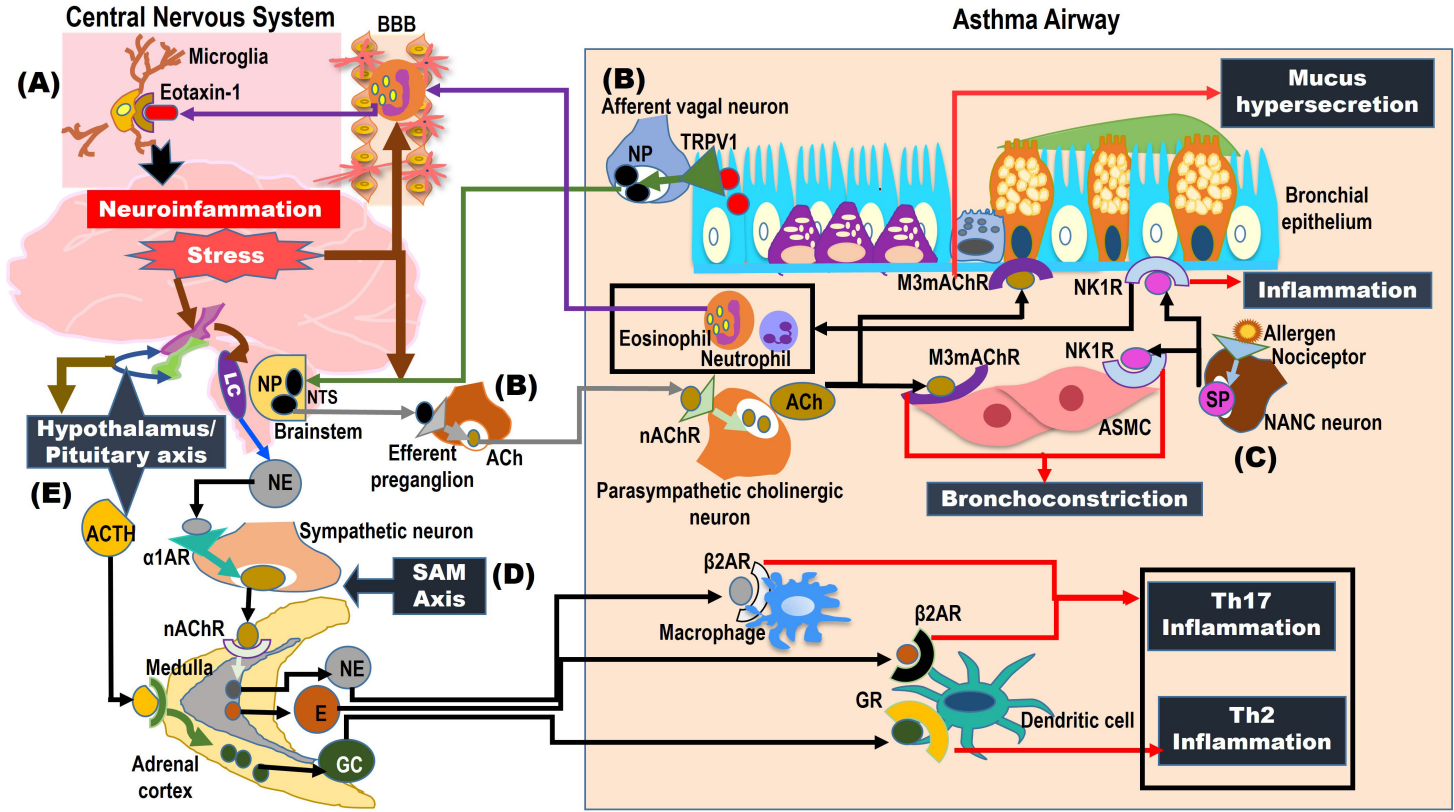
Neuro-immune interactions triggered by psychological stress in ALM. The figure illustrates the humoral and neural pathways that mediate lung-brain crosstalk under the influence of psychological stress in asthma. **(A)** In the humoral pathway, chronic airway inflammation in asthma leads to increased BBB permeability, causing pulmonary eosinophils to penetrate the BBB and release eotaxin-1 that activates microglia in the brain region, contributing to neural inflammation. **(B)** Neural mechanisms involve the afferent vagal neurons that innervate inflamed allergic asthmatic airways, which are activated at their nociceptors, such as TRPV1, by inflammatory mediators. Activated TRPV1 transmits the signals to NTS in the brainstem, where the vagal neuron receptors release NPs, which in turn activate efferent parasympathetic cholinergic neurons to release ACh. The ACh signaling via M3mAChR in ASMC and goblet epithelial cells contributes to bronchoconstriction and mucus hypersecretion, respectively. **(C)** Another parasympathetic e-NANC neurons that innervate the allergic airways are activated by chemical irritants or allergens, which under the influence of psychological stress, release increased levels of neuropeptide SP in asthmatic airways. The SP signaling via NK1R in ASMC causes bronchoconstriction and in epithelial cells stimulates the release of chemokines that increase the influx of eosinophils and neutrophils, contributing to the augmentation of airway inflammation. **(D)** Psychological stress activates CRH secretion from the hypothalamus, which in turn stimulates NE secretion from the LC of the brain stem and ACTH secretion from the pituitary gland. The LC/NE signaling activates the sympathetic adrenomedullary (SAM) axis, releasing epinephrine and norepinephrine, which, through β2AR, stimulate macrophages and dendritic cells in airways, leading to augmentation of Th2 and Th17 inflammation, contributing to asthma exacerbation. **(E)** The ACTH derived from the HPA axis activates the secretion of GC by the adrenal cortex, and this GC/GR signaling in dendritic cells augments Th2 inflammatory response in asthmatic airways.

A systems biology methodology is developed in this study to conduct a systematic literature review of the current understanding of the airway and CNS microenvironments in asthma. This study conducts a systematic review of the current understanding of the airway and CNS microenvironments in asthma. The goal is to construct a molecular systems architecture—a visual, multi-layered depiction of the interactome encompassing molecular interactions within and between airway structural cells, immune cells, and neuronal cells in these microenvironments. In asthma, the anatomical components of the airway and CNS microenvironments, as illustrated in **Figures 1** and **2A–E**, are essential for the development of this molecular systems architecture. Insights from this study seek to deliver the asthma research community a systems-level, visual representation of the intricate biomolecular interactions underlying asthma pathogenesis, including key targets, inter-microenvironment crosstalk between airway and CNS compartments, and a framework for computational modeling to inspire novel therapeutic strategies. Hence, in contrast to conventional pathway-based systems biology models, which focus on isolated linear pathways with sequential steps, the systems architecture framework accelerates therapy development through computational modeling of asthma as dynamic, multi-scale networks integrating direct cell-cell crosstalk and emergent interactions—enabling holistic, personalized drug design based on disease severity.

In complex diseases, networked systems of molecular pathways span multiple organs, cell types, organelles, and compartments. The authors of this work, in previous studies, have applied a systems biology approach to develop a methodology that enables the identification, aggregation, and integration of the signaling cascades at the cellular level to develop molecular systems architectures. Such molecular systems architectures have been successfully developed to reveal systems-level understanding of neurovascular disease (6), acute myeloid leukemia (7), periodontal disease (8), C1 metabolism in plants (9), low-grade chronic inflammation (10), and liver detoxification (11).

Asthma is a chronic airway inflammatory disease with an allergic pattern of airway inflammation that is associated with airway hyperresponsiveness, mucus hypersecretion, and airway remodeling (12). Pathology of asthma is triggered by the combination of genetic and multiple environmental factors, which initiates the activation of T helper (Th) cell immune response that orchestrates the accumulation of inflammatory cells such as macrophages, mast cells, eosinophils, and neutrophils, eliciting deleterious effects on lung epithelium, contributing to the allergic pattern of airway inflammation (3, 13). Based on the molecular pathogenesis of asthma involving the inflammatory and immune cells, asthma is classified into two subtypes: type 2 (Th2-high subtype) and non-type 2 (Th2-low subtype) asthma (14).

Genetic variations, environmental pollutants like tobacco smoke, particulate matter with a size of 2.5–10 microns, pest allergens, nitrogen dioxide, and ozone, respiratory tract infections, lifestyle disorders including vitamin D deficiency, obesity, effects of microbiome, and stress increase the risk of development of asthma (15, 16). Co-morbidities such as gastroesophageal reflux, obstructive sleep apnea, and chronic rhinitis can play a critical role in asthma exacerbations (17). Alveolar epithelial cells are the primary defense immune mechanism of the lung and act as a physical barrier against environmental factors including outdoor pollutants like cigarette smoke, diesel exhaust, particulate matter (PM), laundry, house hold chemicals, microplastics and biological allergens such as pollen, house dust mite (HDM), cockroach, respiratory viruses, fungi and bacteria (18, 19). Reports reveal that some outdoor pollutants (PM 2.5) and proteases derived from biological allergens can facilitate the proteolytic cleavage of epithelial cell-cell tight junctions that regulate the maintenance of structural integrity. This disruption of airway epithelial barrier integrity has been found to trigger the immune system on the mucosal surface, promoting lung inflammation, which is an active player, initiating the pathophysiology of asthma (18). Activation and opening up of epithelial cell-cell tight adherens junctions induce the secretion of epithelial-derived cytokines such as TSLP, IL-33, IL-25, and GM-CSF, which promote the Th2 immune response, driving the airway eosinophilic inflammation (20).

Antigen-presenting dendritic cells (DCs) reside below the lung epithelial layer in the airway microenvironment. Numerous studies have revealed the critical role of pattern recognition receptors (PRRs) like Toll-like receptors (TLRs), expressed by both DCs and epithelial cells, in the recognition of a wide range of environmental pathogens, which initiates an adaptive immune response (21). Among the inflammatory cells, eosinophilic infiltration causes mild to moderate allergic asthma, triggered by Th2 lymphocytes. Neutrophilic infiltration of airways exacerbates asthmatic symptoms mediated by Th1 and Th17 lymphocytes, contributing to the severity of disease (20). Alterations in bronchial epithelium and smooth muscles resulting in mucus hyperproduction by goblet cell hyperplasia and airway smooth muscle cell (ASMC) hypertrophy, respectively, lead to airway thickening that contributes to subepithelial fibrosis under chronic conditions and subsequent airway remodeling – a central pathomorphological feature of bronchial asthma, as depicted in **Figure 1** (22). Furthermore, the psychological stress is identified as a major triggering factor, driving the neuro-immune axis, where the airway inflammation in asthma is transmitted to the CNS, as shown in **Figure 2A** (23, 24). This activates the associated brain regions to elicit responses mediated by sensory, parasympathetic, and sympathetic nerves, which release neurotransmitters in asthmatic airways, increasing the airway inflammation and bronchoconstriction, thereby contributing to asthma exacerbations, which are illustrated in **Figures 2B–E** (23, 24).

Epidemiological studies indicate that asthma affects about 300 million people worldwide and is more prevalent in boys and women (16, 25). Therapies for asthma management include bronchodilators (e.g., β-agonists, theophylline, and anticholinergics), followed by anti-inflammatory agents (e.g., corticosteroids, leukotriene receptor antagonists, and cromones). Current therapeutic approaches provide only symptomatic treatment without curative effects and can cause adverse side effects, including asthma exacerbations and neuropsychiatric symptoms (3, 4).

The Global Initiative for Asthma (GINA) has identified three distinct “endotypes” of asthma: early-onset atopic asthma responsive to steroids, late-onset eosinophilic asthma associated with steroid resistance, and asthma responsive to leukotriene modifiers, each with distinct molecular mechanisms and treatment responses based on pathophysiological mechanisms (22). Given the heterogeneous nature of asthma disease, this study hypothesized that the development of a molecular systems architecture of the airway microenvironment in asthma will provide the following:

1. Multi-layered visual map of biomolecular interactions in the airway microenvironment.
2. Understanding of the complex crosstalk in the airway microenvironment.
3. Potential targets for drug development.
4. Framework to develop computational models providing quantitative predictions.

### 1.1 Significance statement

Systems biology approaches hold great potential for advancing a systems-level understanding of asthma pathogenesis, which involves intricate interactions among multiple cell types. The molecular systems architecture represented herein applies an engineering systems approach to systems biology, wherein, specifically, a multi-layered visualization is provided that interconnects triggers that affect the anatomy to molecular pathways that influence specific biological processes that lead to the pathogenesis of disease. In addition, this architecture is also represented by a “wiring diagram” as well as a web-enabled version to provide the details of molecular interactions within and across cells. This architecture provides understanding of the complex neuroimmune biomolecular interactions between the airway and CNS microenvironment, driven by psychological comorbidities, that amplify airway inflammation and trigger asthma exacerbations. In the future, this architecture will enable the development of predictive, quantitative computational models to identify combination therapies, clinical solutions, and strategies to minimize adverse side effects.

## 2 Methods

### 2.1 Study design

The scientific literature is searched to identify journal papers that contain research on the pathobiology of asthma, molecular interactions between the environmental triggers and airway structural cells, molecular signaling pathways involved in cellular crosstalk between lung resident immune cells and the airway structural cells, and neuroimmune molecular interactions across the lung-brain axis triggered by psychological stress. CytoSolve, a systems biology tool used in this study supports curation, organization and collaborative review of scientific articles to enable systematic bioinformatics literature reviews. The CytoSolve^®^ tool has been applied to diverse areas in systems biology, such as osteoarthritis (26), neurovascular diseases (6), acute myeloid leukemia (7), periodontitis (8), amyotrophic lateral sclerosis (27), and mesenchymal stromal cells (28). The CytoSolve^®^ Operating Guide used in the methodology herein, has been previously published (7, 8, 10).

### 2.2 Literature search and review process

The systems architecture development process includes searching and reviewing the public repository of scientific literature. CytoSolve^®^ (29) was employed to perform a systematic bioinformatics literature review, enabling screening, archiving, distributed collaborative review, and curation of relevant publications. The literature search and review process involved the following steps:

1. A list of keywords was generated (**Supplementary Table 1**), consisting of MeSH terms “Asthma”, “Inflammation”, “Fibrosis”, and “Pathogenesis”, combined with the Boolean operator “AND”, to improve the retrieval of relevant articles.
2. Peer-reviewed articles relevant to the topic, published from January 2008 to March 2025, were searched and retrieved from PubMed (encompassing MEDLINE) using identical MeSH keywords to form the Initial Set. Google Scholar was used only to obtain full-text versions of articles initially identified in PubMed.
3. Relevant articles were shortlisted from the Initial Set based on title and abstract screening to create the “Final Set”.
4. A full-text review was performed on the peer-reviewed articles included in the Final Set.

### 2.3 Eligibility criteria

The full text of the articles, not only the abstracts, was reviewed completely by the authors. Articles were deemed relevant only if the body of the text contained one or more of the keywords from **Supplementary Table 1** in the **Supplementary Material** and was directly related to the pathobiological process of asthma. The following exclusion criteria were applied during screening: unpublished literature and abstracts (due to insufficient peer review); studies on other pulmonary diseases unrelated to asthma pathogenesis; asthma studies lacking molecular pathway details; and review articles on asthma pathogenesis published after 2015 were prioritized to focus on contemporary insights and minimize redundancy in well-established pathological pathways. Studies unavailable in full text or published in non-English languages were also excluded. The quality assessment of the papers selected for the study involved the following process: criteria: 1) Did the paper have a clear hypothesis and objectives; 2) Did the paper have appropriate experimental design; 3) Are the methods transparent; and, 4) Does the paper have adequate sample size for the experiments conducted. Only studies that met all of these criteria were shortlisted for further review.

### 2.4 Study selection and data extraction

Duplicates were removed, and two reviewers independently screened the titles and abstracts of the retrieved studies against the predefined inclusion and exclusion criteria. Studies judged as potentially relevant were advanced to full-text review by the two independent reviewers. Any disagreements regarding study eligibility were resolved through discussion until consensus was reached, or by consultation with a third reviewer when necessary, ensuring PRISMA compliance. Based on PRISMA guidelines (30), the identification, screening, eligibility assessment, and final inclusion of studies in this systematic review are illustrated in the PRISMA flow diagram (**Supplementary Figure 8**).

## 3 Results

The CytoSolve^®^ systematic bioinformatic analysis resulted in the molecular system architecture of asthma pathogenesis, represented in: 3.1) a multi-layered systems architecture schematic; 3.2) a systems architecture interactome of the molecular pathways; and 3.3) subsystems of the interactome.

### 3.1 Multi-layered schematic of the molecular systems architecture

**Figure 3:**
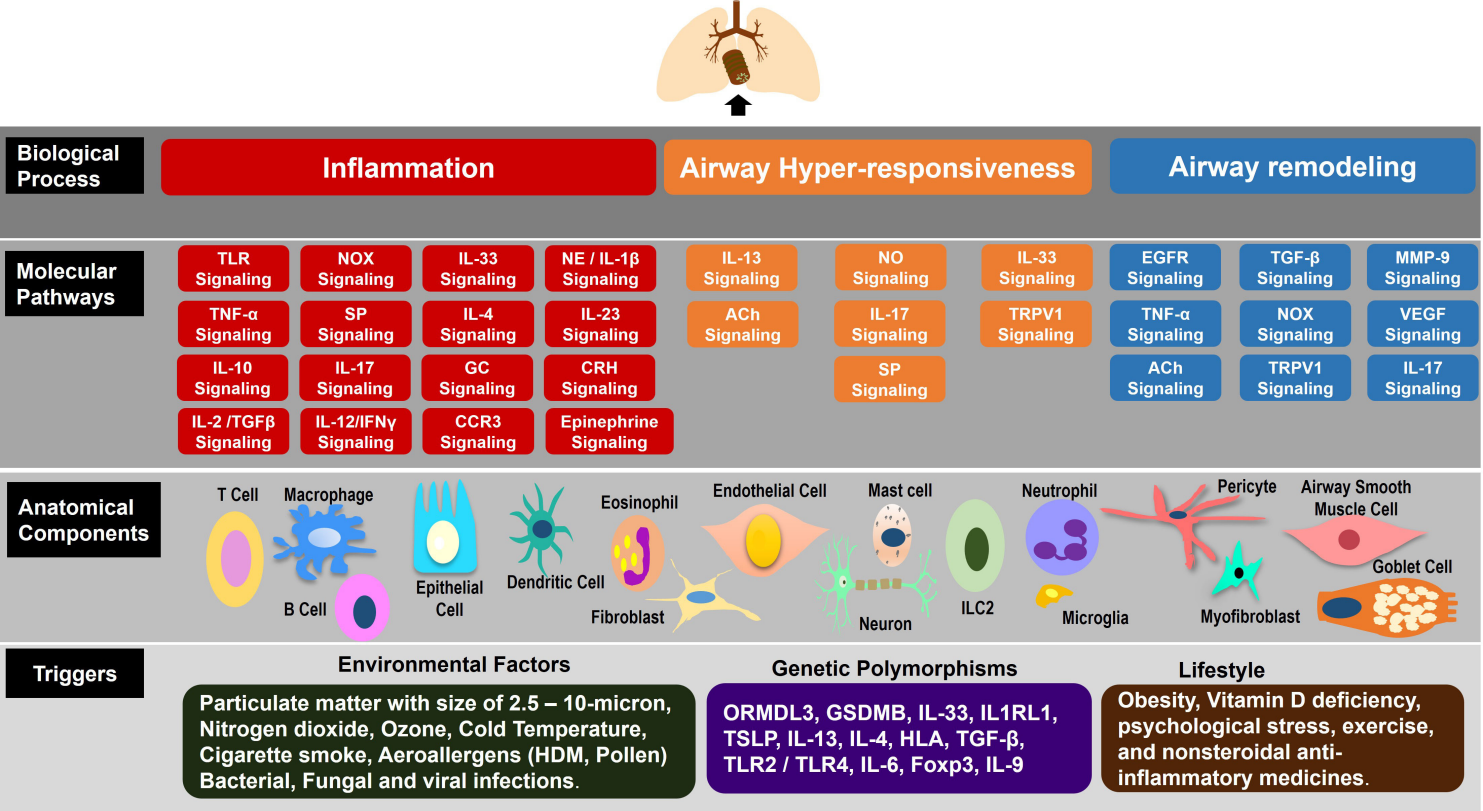
Molecular systems architecture of airway microenvironment signaling in Asthma. In the four-layered architecture, the bottom layer, Triggers, consists of potential triggers: environmental factors, genetic polymorphisms, and lifestyle factors that are implicated in affecting the anatomical components leading to dysfunction in molecular pathways of asthma pathogenesis. The second layer from the bottom, Anatomical Components, consists of the anatomical components of the airway microenvironment (AME): epithelial cells, dendritic cells, T effector cells, innate lymphoid cells 2 (ILC2), eosinophils, neutrophils, mast cells, B cells, airway smooth muscle cells, fibroblasts, and endothelial cell that are involved in the pathogenesis of asthma. The third layer from the bottom, Molecular Pathways, consists of the molecular pathways within and among the anatomical components. The top layer, Biological Processes, reveals the three biological processes: Airway Inflammation, Airway Hyperresponsiveness, and Airway Remodeling, resulting from the dysfunction in molecular pathways of the airway microenvironment, leading to asthma.

There are four layers to the molecular systems architecture schematic of asthma pathogenesis, as shown in **Figure 3**, and are described in detail below.

#### 3.1.1 The triggers layer

The Trigger layer consists of genetic, environmental, and lifestyle factors. Among these genes, the most widely studied gene polymorphisms associated with asthma patients, affecting the lung function, include IL33, IL1RL1, IL13, thymic stromal lymphopoietin (TSLP), and HLA (31). Specifically, polymorphisms in genes such as IL1RL1, TSLP, IL33, IL4, and IL13 has been directly implicated in type 2 immune response (31). Polymorphisms in the ORMDL sphingolipid biosynthesis regulator 3 (ORMDL3) and gasdermin B (GSDMB) genes have been linked to childhood asthma (32). Exposure to environmental pollutants interacts with individual genetic susceptibility to affect the pathogenesis of asthma. Potential environmental risk factors for asthma include exposure to viruses, fungi, proteolytic aeroallergens, cigarette smoking, particulate matter, and diesel smoke (31, 33). Lifestyle disorders such as obesity, vitamin D deficiency, medicines such as non-steroidal anti-inflammatory drugs, and psychological stress increase the risk of the development and progression of asthma (15, 16).

Psychological stress resulting from various lifestyle factors, like violence, lower socioeconomic status, occupational and domestic problems, and academic examinations, has been shown to augment airway inflammation, contributing to the severity of asthma (34–36). According to reports, 20-35% of asthma patients experience that disease exacerbation symptoms are found to be strongly influenced by psychological stress (37, 38). According to Chen et al., negative emotions like fear and anger can increase the Th-2 pathway’s sensitivity, which augments the immune response to environmental triggers, leading to an exacerbation of asthma (34). Thus, psychological stress is a recognized risk factor that influences the worsening of asthma symptoms, and the therapeutic management of asthma (34, 37, 39).

Moreover, other emotional stressors, like depression or anxiety, have been reported to stimulate airway constriction in adult asthma patients (40). Recent reports from meta-analyses studies also indicate that prenatal and early life stress significantly increases the risk of developing asthma-related phenotypes in children. Prenatal stress alters fetal lung growth and disrupts the development of the immune, neuroendocrine, and autonomic systems, which predisposes children to an increased risk of asthma onset following environmental exposures (37, 41–44). Chronic airway inflammation in asthma disrupts the homeostasis of the neuroimmune environment of the CNS, which promotes the development of neurological comorbidities such as depression, anxiety, attention deficit hyperactivity disorder (ADHD), autism spectrum disorders (ASD), and cognitive impairment (23, 45). Psychological stress-induced neuronal cell death in the hippocampal region of the brain of adults with asthma is found to contribute to cognitive impairment (45, 46).

#### 3.1.2 The anatomical components layer

The second layer from bottom, as shown in **Figure 3**, represents cellular components of the airway and CNS microenvironment: bronchial epithelial cells, dendritic cells, macrophages, innate lymphoid cells, CD4+ naïve T cells, Th1 cells, Th2 cells, Th17 cells, Treg cells, CD8+ T cells, eosinophils, neutrophils, B cells, mast cells, basophil, ASMC, fibroblasts, myofibroblast, endothelial cells, pericytes, microglia, adrenal cortex and medulla cells, mucosal gland cells, neurons such as paraventricular nuclei (PVN) of hypothalamus, amygdala brain neurons, brainstem neurons, pituitary neurons, sympathetic neurons, afferent vagal sensory neurons, efferent parasympathetic cholinergic and noncholinergic neurons. Interactions among these thirty-one cell types occur across twenty-five molecular pathways that are implicated in lung dysfunction of three biological processes leading to asthma.

#### 3.1.3 The molecular pathways layer

The third layer from the bottom, as shown in **Figure 3**, across the multiple molecular pathways in the airway microenvironment. Dysfunction in these molecular pathways leads to biological processes causing airway pathogenesis. These twenty-five pathways are: TLR signaling, NOX signaling, TNF-α signaling, IL-33 signaling, IL-4 signaling, IL-12/IFNγ signaling, IL-6/TGFβ signaling, IL-17 signaling, IL-2/TGFβ signaling, NO signaling, IL-13 signaling, EGFR signaling, TGF-β signaling, VEGF signaling, MMP-9 signaling, IL-10 signaling, CCR3 signaling, TRPV1 signaling, acetylcholine signaling, SP signaling, CRH signaling, NE/IL-1β signaling, Epinephrine signaling, glucocorticoid signaling, and IL-23 signaling.

#### 3.1.4 The biological processes layer

The top layer, as shown in **Figure 3**, identifies the three biological processes of asthma pathogenesis: airway inflammation, airway hyperresponsiveness, and airway remodeling. Airway inflammation is affected by TLR signaling, NOX signaling, Tumor necrosis factor alpha (TNF-α) signaling, IL-33 signaling, IL-4 signaling, IL-12/Interferon gamma (IFNγ) signaling, IL-6/TGFβ signaling, IL-17 signaling, IL-2/TGFβ signaling, CRH signaling, NE/IL-1β signaling, GC signaling, SP signaling, CCR3 signaling, and Epinephrine signaling. Airway hyperresponsiveness is affected by IL-33 signaling, NO signaling, IL-13 signaling, IL-17 signaling, SP signaling, TRPV1 signaling, and ACh signaling. Airway remodeling is affected by EGFR signaling, TGF-β signaling, NOX signaling, TNF-α signaling, MMP-9 signaling, IL-17 signaling, VEGF signaling, TRPV1 signaling, and ACh signaling. The biological processes of airway inflammation, airway hyperresponsiveness, and airway remodeling, individually and in combination, drive asthma pathogenesis.

#### 3.1.5 Central pathways

Of the 25 pathways, the most frequent literature support across the curated Final Set (n=764; PRISMA diagram in **Supplementary Figure 8**) include TLR/NOX/TNF-α signaling representing airway inflammation, TGF-β/VEGF signaling representing airway remodeling, and CRH/NE-IL1 signaling representing the neuroimmune axis. This curation highlights evidence strength, informing target prioritization for future development of quantitative mathematical models of asthma pathogenesis.

### 3.2 Interactome of the molecular systems architecture

**Figure 4:**
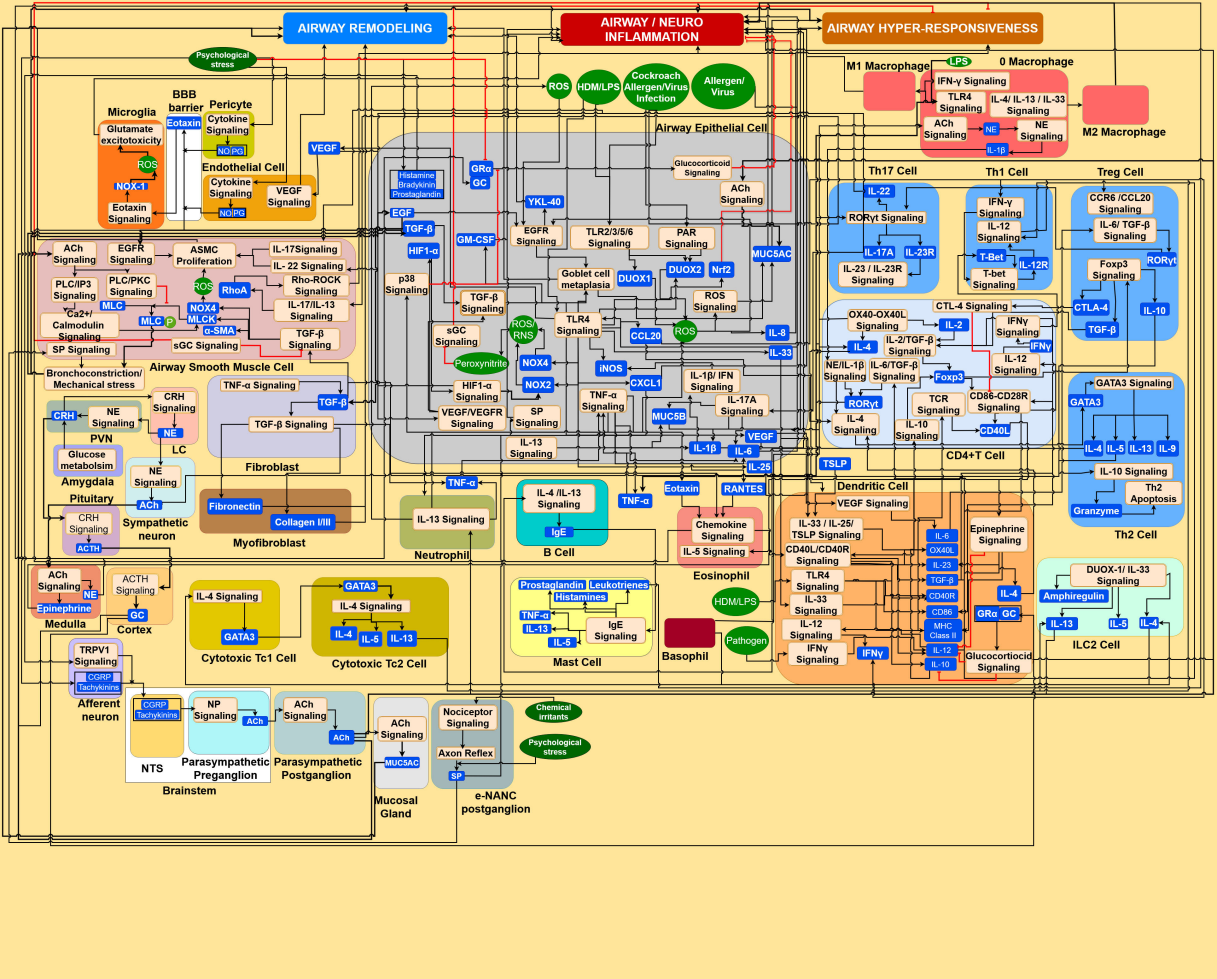
Interactome of the molecular systems architecture of the airway microenvironment involved in asthma pathogenesis. Molecular pathways interact across the airway structural cells (epithelial cell, airway smooth muscle cell, endothelial cell, and fibroblast) as well as across multiple lung resident immune cells: dendritic cell, macrophage, CD4+ T cell, Th1 cell, Th2 cell, Th17 cell, Treg cell, B cell, mast cell, ILC2 cell, CD8+ T cell, neutrophil and eosinophil.

Cells in the airway microenvironment interact with each other via a myriad of molecular pathways. These molecular interactions – the interactome as shown in **Figure 4** – occur across bronchial epithelial cells, dendritic cells, macrophages, innate lymphoid cells, CD4+ naïve T cells, Th1 cells, Th2 cells, Th17 cells, Treg cells, CD8+ T cells, eosinophils, neutrophils, B cells, mast cells, basophil, ASMC, fibroblasts, myofibroblast, endothelial cells, pericytes, microglia, adrenal cortex and medulla cells, mucosal gland cells, neurons such as PVN of hypothalamus, amygdala brain neurons, brainstem neurons, pituitary neurons, sympathetic neurons, afferent vagal sensory neurons, efferent parasympathetic cholinergic and noncholinergic neurons. **Figure 4** provides details of some of the key molecular interactions underlying the Molecular Pathways layer in **Figure 3** that affect the airway inflammation, airway hyperresponsiveness, and airway remodeling that are identified in the Biological Processes layer of **Figure 3**. **Supplementary Table 2** in the **Supplementary Materials** provides the legend for the symbols used in all interactome figures.

### 3.3 Interactome of subsystems of molecular systems architecture

The details of the interactome are provided in the **Supplementary Information**, which contains the 5 subsystems: TNF-α signaling (**Supplementary Figure 1B**); IL-6 and TGF-β Signaling (**Supplementary Figure 2C**); IL-33/PAR1 signaling (**Supplementary Figure 2A**); MMP-9 signaling (**Supplementary Figure 5**); Macrophage polarization (**Supplementary Figure 10**); Epinephrine signaling (**Supplementary Figure 7**). The macrophage polarization leading to asthma pathogenesis is elaborately documented in the **Supplementary Data** of **Supplementary Material**.

### 3.4 Crosstalk signaling across cellular components of the airway microenvironment

This section is organized as follows: 1) Interactive crosstalk between airway epithelial cells, fibroblasts, and inflammatory cells; 2) Interactive crosstalk among airway epithelial cells, ASMC, dendritic cells, and inflammatory cells; 3) Interactive crosstalk between epithelial cell, smooth muscle cell, fibroblast, neutrophil, and eosinophil; 4) Interactive crosstalk between T Cells, airway epithelial cells, eosinophils, and airway smooth muscle cell; 5) Interactive crosstalk among airway structural cells, immune cells, central and peripheral neurons; 6) Important findings in the recent literature.

#### 3.4.1 Interactive crosstalk between airway epithelial cells, fibroblasts, and inflammatory cells

Imbalance in pro- and anti-oxidant mechanisms leads to the production of excess ROS, contributing to oxidative stress in the lung. ROS has been widely studied for its deleterious effects on the airway epithelium and for being implicated in the initiation of asthma pathogenesis (47). Respiratory viral infections, environmental pollutants, including cigarette smoke, ozone, particulates, and ionizing radiation, are the known major sources of exogenous ROS (47–49). Airway epithelial cells and inflammatory cells are potential sources of endogenous superoxide anions in asthma (47, 50). The pathogenesis of asthma is influenced by the deleterious effects of oxidative stress on airway structural cells, which result in (i) inflammation sustained by airway epithelial cells, (ii) ASMC causing airway hyperresponsiveness, and (iii) ASMC and fibroblasts promoting airway remodeling or fibrosis (51). Environmental pollutants can promote a high level of oxidative stress and subsequent airway inflammation via the activation of pattern recognitions receptors, mainly TLRs, protease-activated receptors (PARs), and their downstream signaling target molecules like MAPK and NF-κB,s which have been found to play a critical role in the development of asthma pathogenesis (19, 47). Key signaling transduction pathways implicated in the ROS-induced asthma pathogenesis include NOX signaling, TLR signaling, and TNF-α signaling.

**Figure 5:**
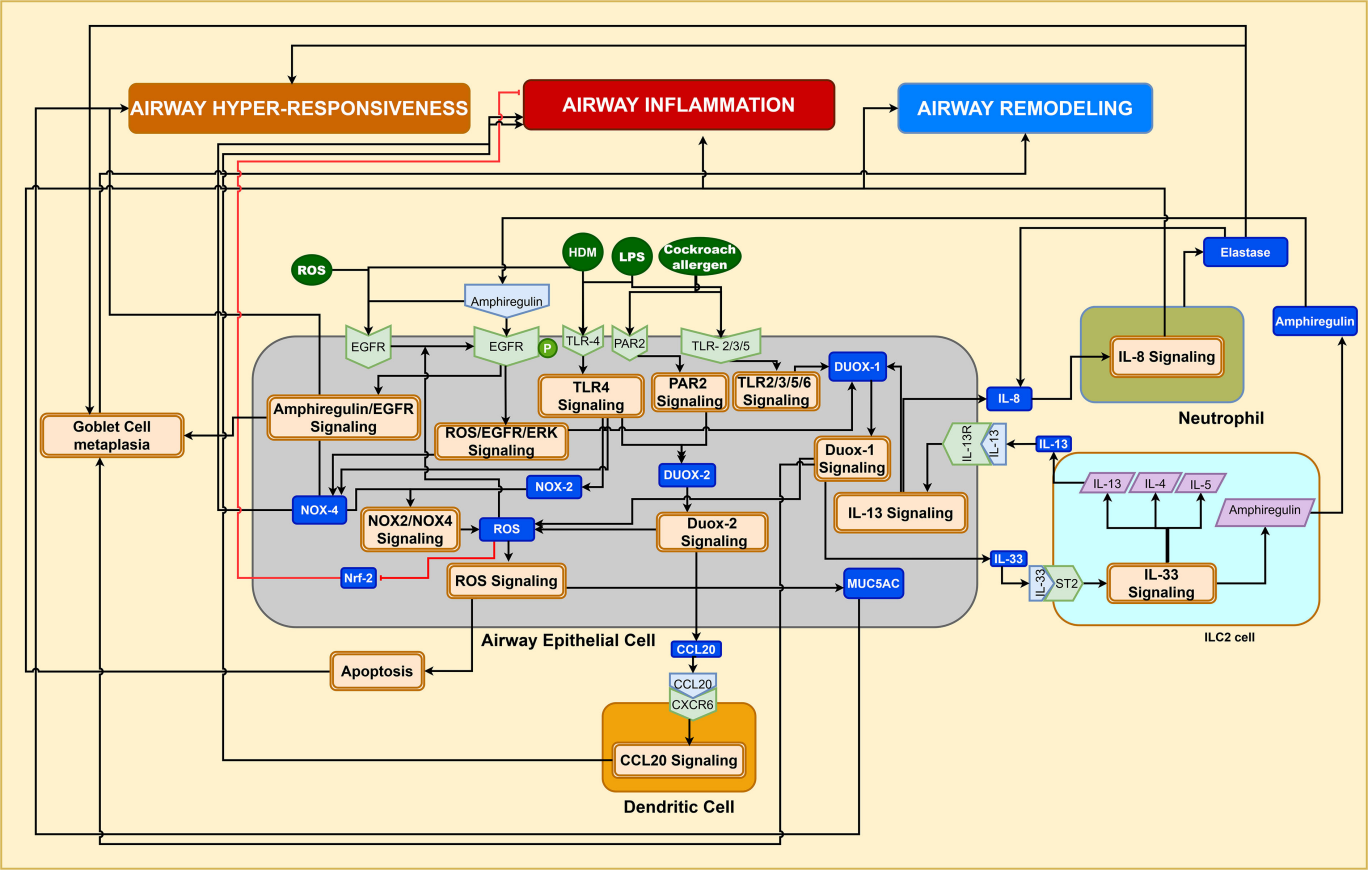
NOX/ROS signaling in the airway microenvironment leads to ROS production, promoting airway inflammation, hyperresponsiveness, and airway remodeling.

Nox signaling: Nicotinamide adenine dinucleotide phosphate (NADPH) oxidases (NOX) and its various isoforms are a major source of endogenous ROS (50, 52). NOX2 is expressed by macrophages, monocytes, neutrophils, and eosinophils, while airway epithelial cells express dual oxidase (DUOX1), DUOX2, NOX2, and NOX4, whereas ASMC express only NOX4 (53). DUOX1 and DUOX2 play a key role in airway inflammation and epithelial cell apoptosis, respectively, in response to triggers like environmental allergens and infections (54). Lipopolysaccharide (LPS) triggered activation of TLR-4 signaling induces activation of DUOX-2, resulting in CCL20 secretion by bronchial epithelial cells. CCL20 chemokine recruits dendritic cells, promoting the Th2 allergic inflammatory response in asthmatic airways (55). DUOX1/2 and NOX-4 mediated ROS signaling facilitates the upregulation of MUC5AC gene expression in airway epithelial cells (54, 56, 57), leading to airway hyperresponsiveness. ROS produced by DUOX-1 promotes oxidation of EGFR, enhancing the ligand-induced EGFR phosphorylation in epithelial cells, which in turn, promotes goblet cell metaplasia leading to airway remodeling, and mucus hypersecretion leading to airway hyperresponsiveness (57–59). Diminished expression of antioxidant gene Nrf-2 were also observed in the presence of HDM triggered NOX2/4 signaling, which contributes to suppression of antioxidant response mediated by SOD2, catalase, and glutathione (52). Comprehensive illustration of NOX signaling is shown in **Figure 5**, and the complete details on NOX isoform signaling mechanisms such as DUOX-1/IL-33 signaling, IL-13 signaling, NOX/Nrf-2 signaling, EGFR/ERK1 signaling, and PAR2/DUOX-2 signaling, are documented in the **Supplementary Data** of **Supplementary Material**.

TLR signaling: Toll-like receptors (TLRs) are transmembrane receptors located on the plasma membrane or endosomal compartments of the cell (60). TLRs are expressed in lung epithelial cells, alveolar macrophages or dendritic cells, and other immune cells (61). Interaction of TLRs with pathogenic ligands, such as pathogen-associated molecular patterns (PAMPs) host-derived damage-associated molecular patterns (DAMPs) activates the TLR signaling that is transduced via two different pathways, as illustrated in **Supplementary Figure 1A**: 1) myeloid differentiation primary response 88 (MyD88); and, Toll/IL-1 receptor-domain containing adapter-inducing interferon-β pathways (TRIF) (60). TLR4/MYD88 signaling pathway in pulmonary dendritic cells promotes the Th2 inflammatory response (60, 62). TLR4/TRIF signaling via the activation of interferon-stimulating factor 3 (IRF3) promotes the Th17 neutrophilic inflammation in asthma (19, 63). Comprehensive details of TLR signaling pathways involved in naïve CD4+Tcell polarization to effector T helper cells are documented in **Supplementary Data**. Additionally, TLR signaling in airway epithelial cells, promoting inflammation, is also documented in **Supplementary Data**. Another key TNF-α signaling is documented in **Supplementary Data**, and schematics of TNF-α signaling are illustrated in **Supplementary Figure 1B**.

#### 3.4.2 Interactive crosstalk among airway epithelial cells, ASMC, dendritic cells, and inflammatory cells

Proinflammatory T helper cells play a critical role in driving airway inflammation in asthma pathogenesis. Interactions between mature dendritic cell and naive CD4+ T cells leads to the polarization of naive CD4+ T cells into Th1, Th2, and Th17 cells in presence cytokines IL-12, IL-4, and IL-6, respectively (21, 64, 65). Treg cells attenuate asthma exacerbation caused by Th2 proinflammatory response. Naive CD4+Foxp3− T cells differentiate into Tregs in the presence of TGF-β, IL-2, retinoic acids, IL-10, and IFN-γ (66–68). The mechanisms of polarization of T helper cells and Treg cells, and their subsequent role in the promotion of airway inflammation, airway hyperresponsiveness, and airway remodeling, are discussed in detail below.

**Figure 6:**
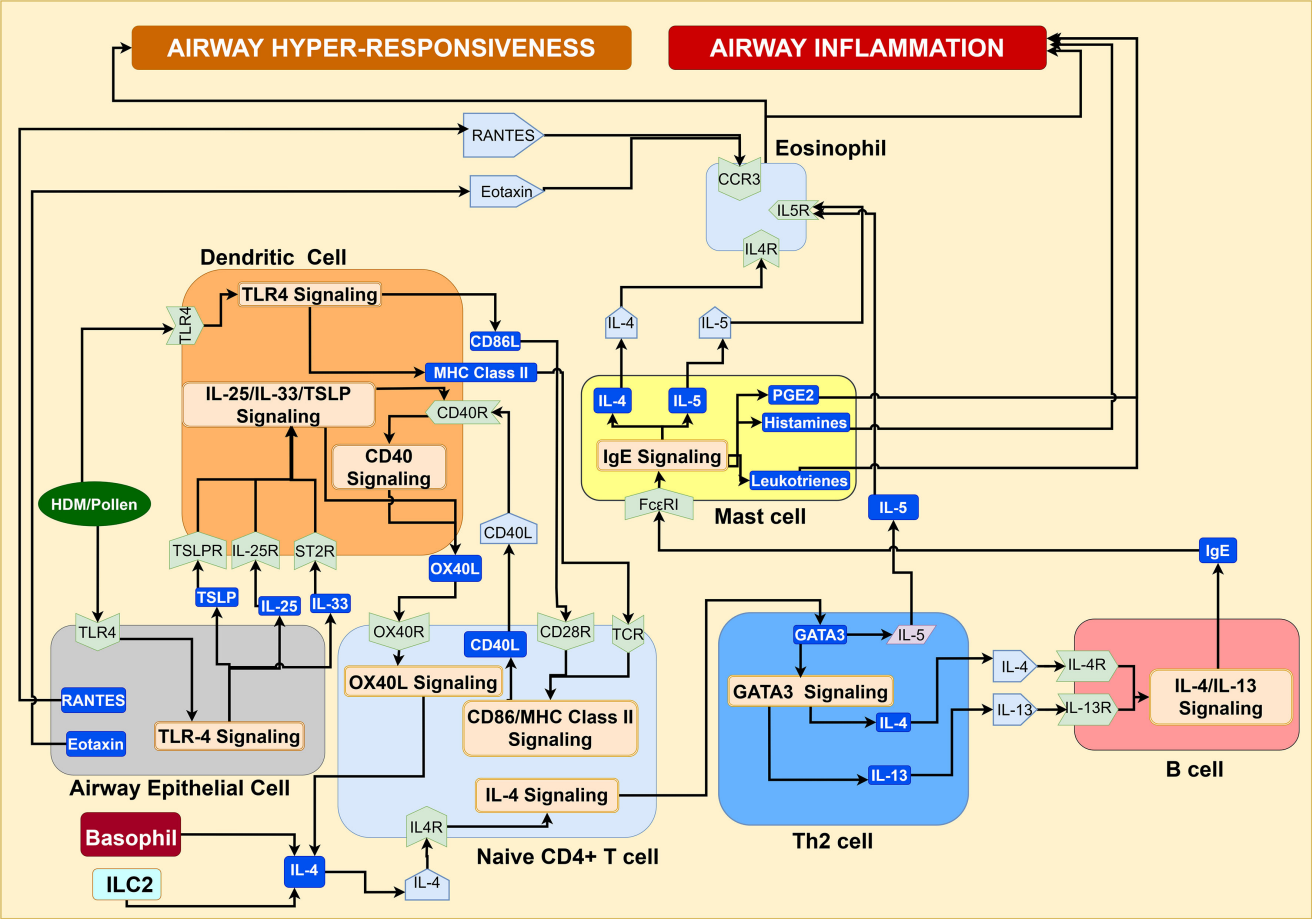
IL-4 signaling in the airway microenvironment promotes the polarization of activated naïve CD4+T cells towards Th2 cell differentiation through the secretion of Th2 cytokines, leading to Th2 eosinophilic airway inflammation, mast cell degranulation, and airway hyperresponsiveness.

IL-4 signaling: Antigen-presenting dendritic cells activate the naive CD4+ T-cells to differentiate into Th2 cells in the IL-4 cytokine-enriched airway microenvironment, promoting airway eosinophilic inflammation and consequent bronchial hyperresponsiveness (20, 67). The IL-4 signaling activates STAT6, which mediates the GATA3 transcription factor expression and that drives the synthesis of Th2 specific cytokines IL-4, IL-5, IL-9, and IL-13, as shown in **Figure 6** (20, 67, 69). Of these cytokines, IL-4 and IL-13 promote IgE production in B cells, leading to activation and degranulation of mast cells, and activation of eosinophils via IL-5 (14, 20). The proinflammatory response of Th2 cells eventually contributes to airway epithelial damage, promotes airway hyperresponsiveness (3, 20), and airway inflammation (14). The IL-4-initiated signaling cascade is further elaborated in **Supplementary Data** of the **Supplementary Materials**. Additionally, the IL-33/PAR2 signaling in bronchial epithelium augments the type 2 inflammation and airway hyperresponsiveness by promoting the interaction between airway epithelial and mast cells, which is discussed in detail in the **Supplementary Data** (see **Supplementary Figure 2A**).

IL-12- IFN-γ signaling: IFN-γ and IL-12 have been reported to act as Th1 polarizing cytokines (70, 71). Macrophage or dendritic cell-derived IL-12 drives the expression of IFNγ in naïve T cell, and both IL-12 and IFNγ upregulate the expression of transcription factor T-bet, which is responsible for differentiation of naïve T cells into Th1 cell, as illustrated in **Supplementary Figure 2B** (70, 72). IFN-γ signaling acts in an autocrine manner and generates a positive feedback loop, resulting in the augmentation of IL-12 secretion (72). IFN-γ signaling has been shown to inhibit the effects on the expression of Th2 cytokine IL-4 via the upregulation of IFN-γ-responsive gene IRF, as seen in **Supplementary Figure 2B** (70, 73). Comprehensive details of the IFN-γ/IL-12 initiated signaling, IFN-γ/SP-1 signaling, and IFN-γ/CXCL10 signaling axis, leading to airway inflammation, are provided in **Supplementary Data**.

IL-6-TGF-β/IL-17 signaling: Th17/Treg imbalance is implicated in Th2-low asthma pathogenesis. Increased IL-17 expression in asthmatic patients with neutrophilic inflammation and corticosteroid resistance has been correlated with disease severity (20, 74). Cytokines produced by the activated DCs, such as IL-1*β*, IL-6, and TGF-β in the airway microenvironment, promote Th17 polarization (20, 65). The IL-6 that was produced during inflammation acts synergistically with TGFβ signaling to promote the differentiation of CCR6+ Treg cells into Th17 cells by inhibiting the Foxp3 expression and Treg cell development. This promotes an increased number of Th17 cells, causing Th17/Treg imbalance in airways as illustrated in **Supplementary Figure 2C**. IL-6 and TGF-β signaling in naïve CD4+T cells promote expression of RORγt, which in turn drives the differentiation of Th17 cells secreting IL-17A, IL-17F, IL-22, and IL-23R, as seen in **Supplementary Figure 2D** (65, 67, 75). The IL-23 cytokine signaling stabilizes the production of Th17 cytokines such as IL-22, thereby promoting the Th17 pathogenicity (76). Further details of IL-6 and TGF-β signaling components and IL-17 signaling in airway epithelial cell and ASMC are elaborated in the **Supplementary Data** of **Supplementary Materials**.

IL-2-TGF-β signaling: Lack of naive CD4+ differentiation into the Treg cell population augments the Th2 proinflammatory response, promoting asthma exacerbations. TGF-β and IL-2 drive the differentiation of naive CD4+CD25+ cells into Foxp3+ pTreg cells, which induces immune tolerance via the secretion of IL-10 and TGF-β (68). TGF-β signaling activates the Smad-3 pathway, whereas IL-2 activates of STAT-5 pathway, leading to Treg differentiation (77). Tregs inhibit airway inflammation by two mechanisms: (1) Inhibitory effects on allergy mediated inflammations through various mechanisms such as suppression Th1/Th2/Th17cells via CTLA-4 receptor, eosinophils and inflammatory dendritic cells, followed by an induction of antibody isotype switching from IgE to IgG4 (69); and, (2) secretion of anti-inflammatory cytokine IL-10 to prevent chronic inflammation in asthma (78), as schematically represented in **Supplementary Figure 2E**. Comprehensive details of the TGF-β-IL-2 signaling cascade and IL-10 signaling, inducing apoptosis in Th2 cells, are described in **Supplementary Data**.

#### 3.4.3 Interactive crosstalk between epithelial cells, ASMCs, fibroblasts, neutrophils, and eosinophils

Airway remodeling is the hallmark of the chronic progression of asthma pathogenesis. Increased permeability of injured epithelium enhances the exposure of underlying subepithelial tissue to inhaled environmental allergens, leading to the release of growth factors from the damaged epithelium, such as EGFR, TGF-β1, and VEGF, that promote airway remodeling (79, 80). These growth factors promote structural changes in airways such as loss of epithelial integrity, goblet-cell hyperplasia, smooth muscle hypertrophy, accumulation of fibroblasts and their differentiation into myofibroblasts, and increased angiogenesis (22, 81). Inactive forms of latent growth factors like TGF-β and VEGF embedded within the extracellular matrix (ECM), undergo proteolytic cleavage mediated by MMP-9, as illustrated in **Supplementary Figure 5**, and details of the MMP-9 pathway are provided in the **Supplementary Data** (81, 82). The effects of EGFR, TGF-β1, and VEGF on airway remodeling are discussed in detail below.

**Figure 7:**
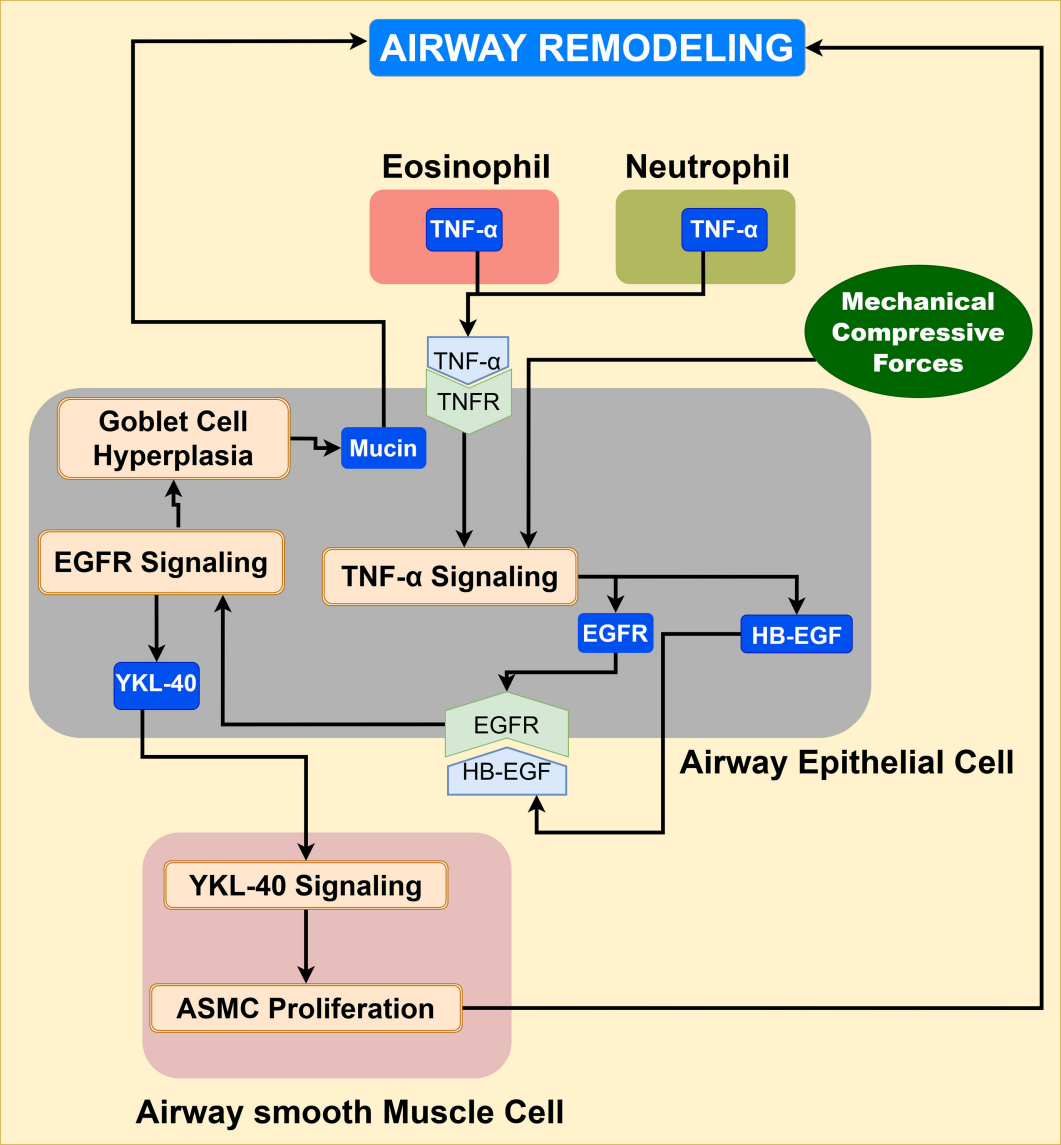
EGFR signaling in the airway microenvironment stimulates smooth muscle cell proliferation, goblet cell hyperplasia, and mucus hypersecretion, leading to airway remodeling of asthmatic airways.

EGFR signaling: Compressive forces from the airway smooth muscle cell contraction during bronchoconstriction and pro-inflammatory cytokine TNF-α from eosinophils and neutrophils upregulate the expression of EGFR and its ligand heparin-bound-EGF (HB-EGF) in the injured airway epithelial cells (83), leading to activation of EGFR signaling, as shown in **Figure 7**. Once activated, EGFR signaling leads to goblet cell hyperplasia and expression of YKL-40 (84). YKL-40 further stimulates angiogenesis, smooth muscle cell proliferation, and migration, contributing to airway remodeling, as depicted in **Figure 7**. Further details of the EGFR signaling pathway are provided in **Supplementary Data** of the **Supplementary Materials**.

TGF-β signaling: TGF-β1 derived from epithelial cells and eosinophils is a major contributor to asthma pathogenesis via airway remodeling and airway hyperresponsiveness (79, 83, 85). As schematically represented in **Supplementary Figure 3A**, ROS generated in asthmatic airway epithelium triggers the production of TGF-β1 by epithelial cells and eosinophils, which in turn augment ROS levels via NOX-4 activation, resulting in fibroblast activation, myofibroblast differentiation, and consequent airway remodeling (47). Additionally, TGF-β1 stimulates the proliferation of airway goblet cells, ASMC, and fibroblasts, while inducing apoptosis in epithelial cells (85, 86). TGF-β1 signaling inhibits the production of MMPs by airway epithelial and inflammatory cells and promotes the synthesis of tissue inhibitors of metalloproteinase (TIMPs) by epithelial cells (87), thereby promoting airway remodeling. Comprehensive details of TGF-β1 signaling pathways mediating ASMC proliferation and fibroblast to myofibroblast transition are provided in the **Supplementary Data** of **Supplementary Materials**.

VEGF signaling: VEGF is a mitogenic peptide that is well known for its potent induction of endothelial cell migration, proliferation, and tubule formation (88, 89). VEGF expression is higher in the lung tissue biopsies from asthmatic patients and is directly correlated with disease severity (88–90). **Supplementary Figure 3B** displayed the VEGF-induced sub-epithelial fibrosis via upregulation of TGF-β in lung epithelial cells, causing accumulation of collagen I, III, and V, leading to thickening of the basement membrane and subsequent airway remodeling (22, 88). VEGF also downregulates expression of E−cadherin and β−catenin, as shown in **Supplementary Figure 3B**, which promotes disruption of tight junction barrier integrity in airway epithelium, leading to increased access of inhaled allergens and consequent exacerbation of Th2 inflammatory response (90). VEGF signaling cascade in epithelial and endothelial cells leading to myofibroblast production and angiogenesis, respectively, has been extensively discussed in the **Supplementary Data**.

#### 3.4.4 Interactive crosstalk between T Cells, airway epithelial cells, eosinophils, and airway smooth muscle cells

Airway hyperresponsiveness is a major pathological manifestation of asthma characterized by bronchoconstriction resulting from interactions among T cells, airway epithelial cells, eosinophils, and ASMC (91). Two key signaling pathways that contribute to hyperresponsiveness include IL-13/IL-17 signaling across Th2, Th17, and airway smooth muscle cell; and, nitric oxide (NO) signaling across ASMC.

IL-13/IL-17 signaling: IL-13 and IL-17, inflammatory mediators from Th2 and Th17, respectively, increase the expression of GTPase RhoA protein in ASMC (92, 93). RhoA activates its downstream target Rho-associated, coiled-coil containing protein kinase (ROCK), which results in upregulation of p-MLC and increased contraction of airway smooth muscle, inducing airway hyper-responsiveness (91, 92). IL-13/IL-17 signaling is illustrated in **Supplementary Figure 4**. Comprehensive details of the IL-13/IL-17 signaling mechanism in ASMC are provided in the **Supplementary Data**.

**Figure 8:**
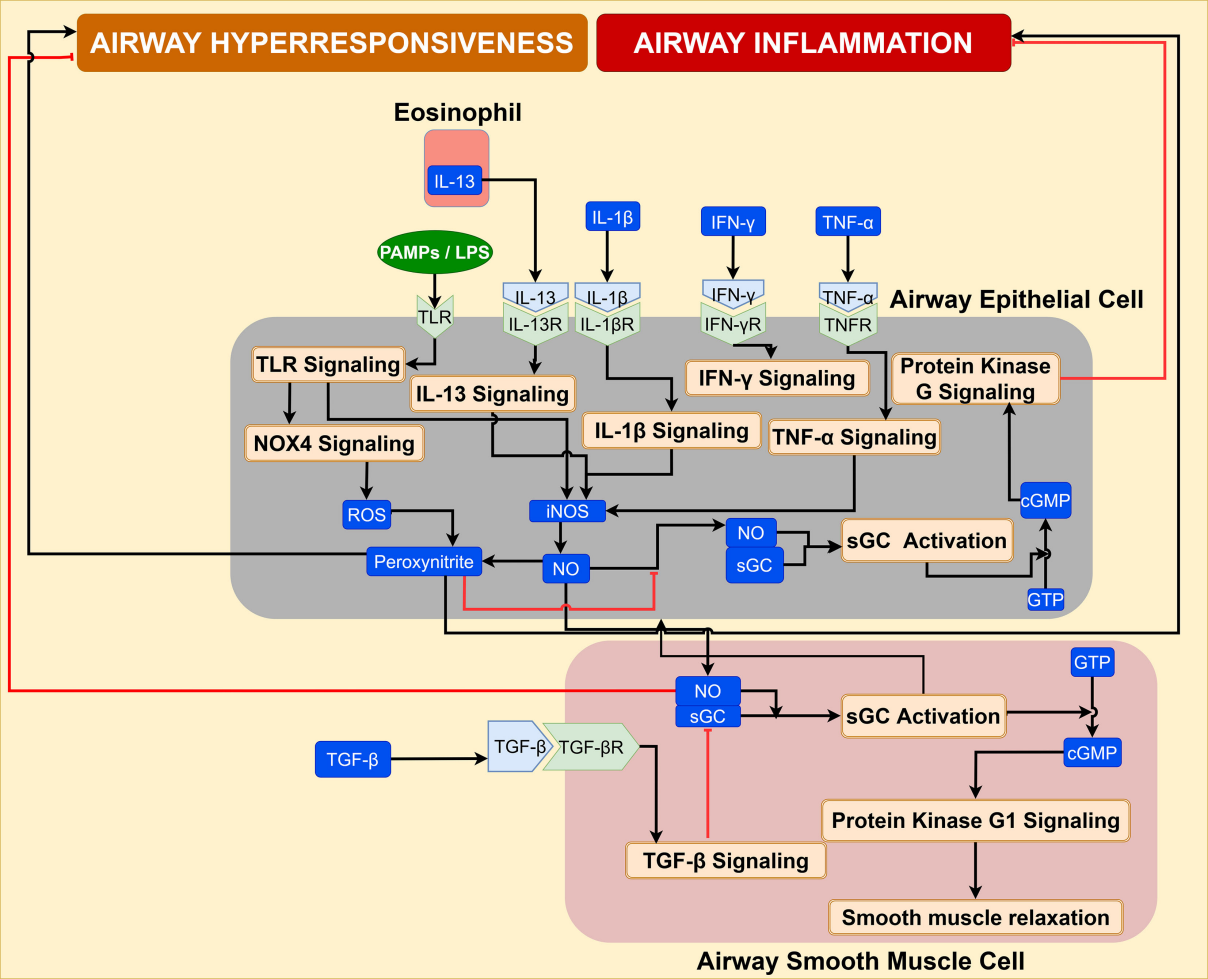
NO signaling is responsible for bronchorelaxation in the airway microenvironment. It is diminished by epithelial ROS, leading to airway hyperresponsiveness.

Nitric oxide signaling: Increased expression of inducible nitric oxide synthase (iNOS) in asthmatic airway epithelium and elevated levels of exhaled nitric oxide (NO) have been observed in asthmatic patients (94, 95). Additionally, IL-13 secretion from eosinophils was shown to upregulate iNOS synthesis in airway epithelial cells (94, 96), resulting in elevated NO production. Inflammatory conditions in the airway epithelial cells upregulate NOX-4, and the ROS generated by NOX-4 react with NO to produce peroxynitrite, thereby lowering the availability of NO for bronchodilation (94, 97). Additionally, diminished expression of soluble guanylate cyclase (sGC), the target of NO, in asthmatic airway epithelial cells leads to depletion of cGMP needed for bronchorelaxation (98). Depletion of NO availability, suppression of sGC expression, and increased peroxynitrite in the airway epithelium and ASMC contribute to hyperresponsiveness and airway inflammation observed in asthma. NO signaling cascade is illustrated in **Figure 8**, and details of NO pathways are comprehensively discussed in **Supplementary Data** of **Supplementary Materials**.

#### 3.4.5 Interactive crosstalk among airway structural cells, immune cells, and central and peripheral neurons

Psychological stress and negative emotions like pain and depression can promote bronchoconstriction and increased airway inflammation, contributing to asthma exacerbation and poor control of asthma symptoms (99). Proinflammatory factors produced during chronic airway inflammation in asthma can be transmitted to the CNS through the activation of neural and humoral pathways, which involve airway sensory nerves and the blood-brain barrier, respectively (23, 99). Psychological stress in asthma can influence the neuroinflammation in brain regions to stimulate the nervous system, activating the Locus caeruleus (LC)/norepinephrine (NE)-autonomic nervous system (ANS), and neuroendocrine axis involving the hypothalamus-pituitary-adrenal axis (HPA) (23, 100). This vicious cycle of lung-brain crosstalk augments airway inflammation, contributing to bronchoconstriction and asthma exacerbations that are accompanied by cognitive decline, anxiety, and depression in asthma patients (23, 101).

Psychological stress exacerbates asthma via brain-lung axis crosstalk, engaging CNS components through established neural and humoral pathways. Animal models provide mechanistic evidence that psychological stress directly activates central nervous system pathways, including the HPA axis and the LC/NE nervous system, thereby amplifying airway inflammation through glucocorticoid dysregulation and neural reflexes. In contrast, human studies offer correlational evidence linking chronic stress, anxiety, and depression to increased frequency of asthma exacerbations and reduced control of symptoms.

Brain-lung axis crosstalk regulates the cellular interactions among (i) structural cells of airways like epithelial cells, smooth muscle cells, fibroblasts, mucosal gland and blood brain barrier including endothelial cell and pericyte; (ii) brain immune cell microglia and airway immune cells like macrophage, eosinophil, dendritic cell, CD4+ T cell, Th2 cell, Th17 cell and Treg cell; CNS neurons such as LC and nucleus of the solitary tract (NTS) of brainstem, PVN of hypothalamus, pituitary neurons and sensory afferent neurons; Peripheral neurons such as efferent parasympathetic neurons and sympathetic neurons; cortical and medulla cells of adrenal gland. This intercellular crosstalk leads to biological processes of airway inflammation, airway hyperresponsiveness, and airway remodeling that are implicated in asthma pathogenesis. Cellular crosstalk between the lung and brain is bi-directional in nature, with stress influencing the pathophysiology of asthma and asthma causing neurodegeneration. There are two main stress systems modulated by the Brain-Lung Axis Crosstalk, including

1. Humoral signaling pathway that governs lung-to-brain communication
2. Neural signaling pathway that governs brain-to-lung and lung-to-brain communications.

The details of these systems are described in detail below

**Figure 9:**
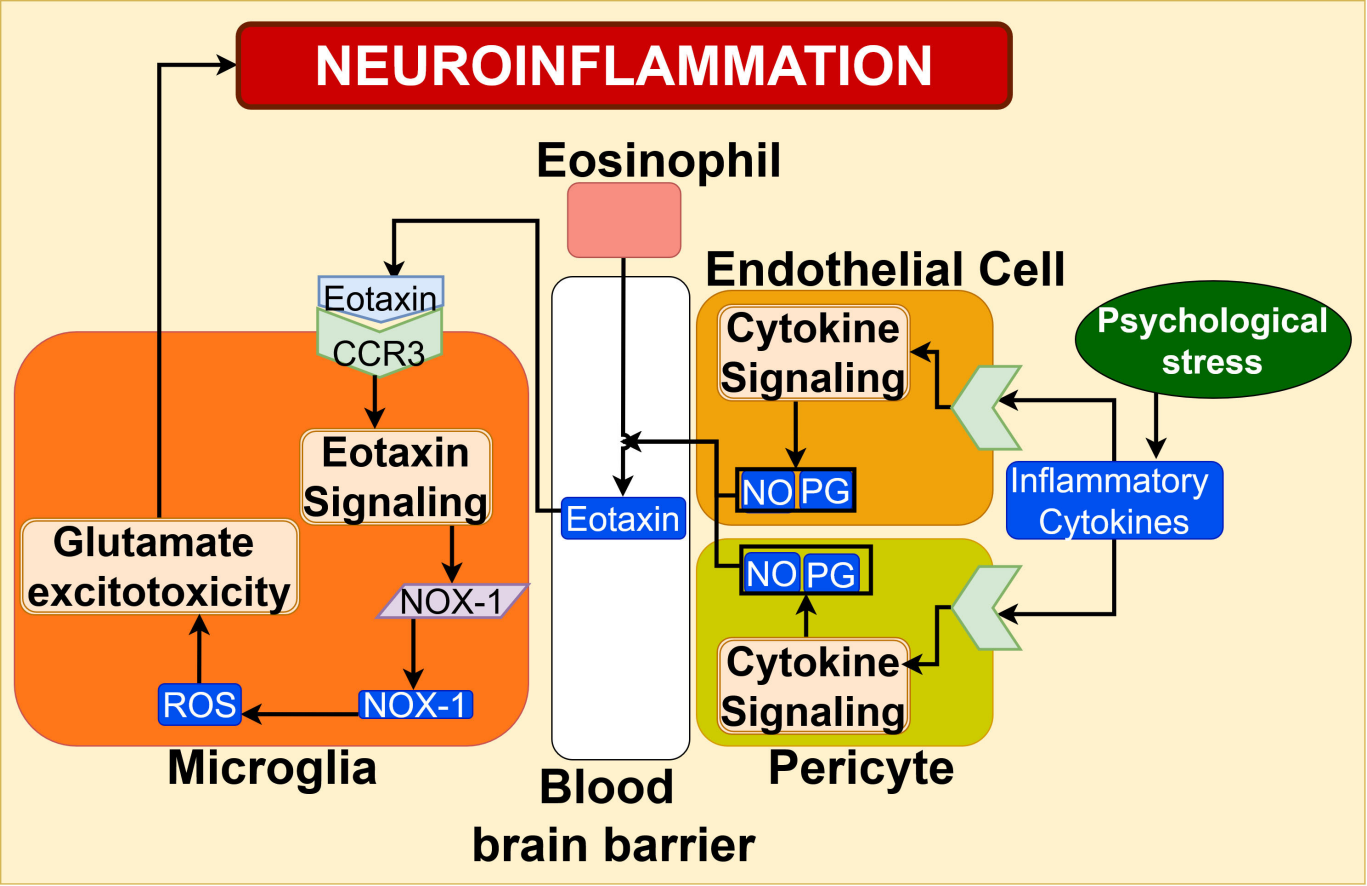
CCR3 signaling in the blood-brain barrier microenvironment, under the influence of psychological stress, stimulates the eotaxin signaling in brain microglia, causing neuronal inflammation, contributing to the exacerbation of asthma pathogenesis.

Eotaxin-1/CCR3 signaling: Preclinical studies show that in the humoral pathway, inflammatory cytokines and chemokines released into the bloodstream from asthmatic airways stimulate the production of prostaglandin (PG) and nitric oxide (NO) by cerebrovascular endothelial cells and pericytes, which make up the blood-brain barrier (BBB). This results in a loss of BBB integrity and an increase in BBB permeability, which in turn leads to increased cytokine infiltration into the central nervous system (23). Infiltrated inflammatory mediators like eotaxin-1 (CCL11) derived from pulmonary eosinophils have been shown to stimulate its C-C Chemokine Receptor 3 (CCR3) receptor on microglia, increasing the NOX1 expression, leading to ROS production, as shown in **Figure 9** (23, 102). This microglial ROS promotes glutamate-induced neurotoxicity and neuronal cell death. These necrotized neuron cells act as DAMP, further activating microglia, thereby perpetuating neuroinflammation (23, 102).

Neural signaling: Neural mechanisms of the ANS play a critical role in triggering airway hyperresponsiveness in asthma, which is predominantly mediated by the sensory vagus nerve fibers that originate from the jugular and nodose ganglia, with their axons extending peripherally into the airways and its other branches projecting centrally into the NTS in the brainstem (99, 103). These vagus nerve fibers is comprised of two components such as afferent nerves and efferent parasympathetic nerves (99). This NTS neuron projects to multiple regions of the brain, such as the anterior cingulate cortex (ACC) and amygdala. Activation of NTS neurons has been found to affect the functional activities of these brain regions, influencing mood, cognition, and sensory processing, which cause depression, cognitive decline, and abnormal respiratory sensations in asthma (23, 99).

The pseudonupilar neurons that constitute the sensory vagus nerve fibers are further categorized as myelinated A-fibers and unmyelinated C-fibers. Mechanoreceptors like rapidly adapting receptors (RARs) and slowly adapting receptors (SARs) of A fibers that innervate the lung parenchyma respond to mechanical stimuli that influence breathing pattern during lung inflation and deflation. Whereas nociceptors of both A and C-fibers respond to both chemical mediators from damaged tissue and mechanical stimuli, and chemoreceptors of C-fibers respond to inflammatory mediators and inhaled chemical irritants (99, 104). These C-fiber receptors are distributed throughout the airway smooth muscle, epithelium, blood vessels, ganglia, and pulmonary neuroendocrine cells (PNEC) or neuroepithelial bodies that are localized in the bronchial epithelium (99, 104–106).

Clinical studies associate bronchial asthma symptoms such as wheezing, chest tightness, and cough with dysfunction of airway sensory nerve fibers (103, 107). Preclinical studies establish four major neural mechanisms involved in this bidirectional neural signaling include (i) Afferent vagal TRPV1 signaling triggered by inflammatory mediators in airways leading to the release of neuropeptides (NPs) in brainstem; (ii) Efferent parasympathetic signaling triggered by NPs in brainstem, which contribute to airway inflammation and bronchoconstriction through the release of neurotransmitters such as acetylcholine (ACh) and neuropeptides in airways (iii) CRH signaling that activates LC/NE-ANS and HPA axis, which increases the airway inflammation through the secretion neurotransmitters like norepinephrine, epinephrine, and glucocorticoids (iv) NE/IL-1β signaling axis augment airway inflammation. These signaling pathways are discussed in detail below.

**Figure 10:**
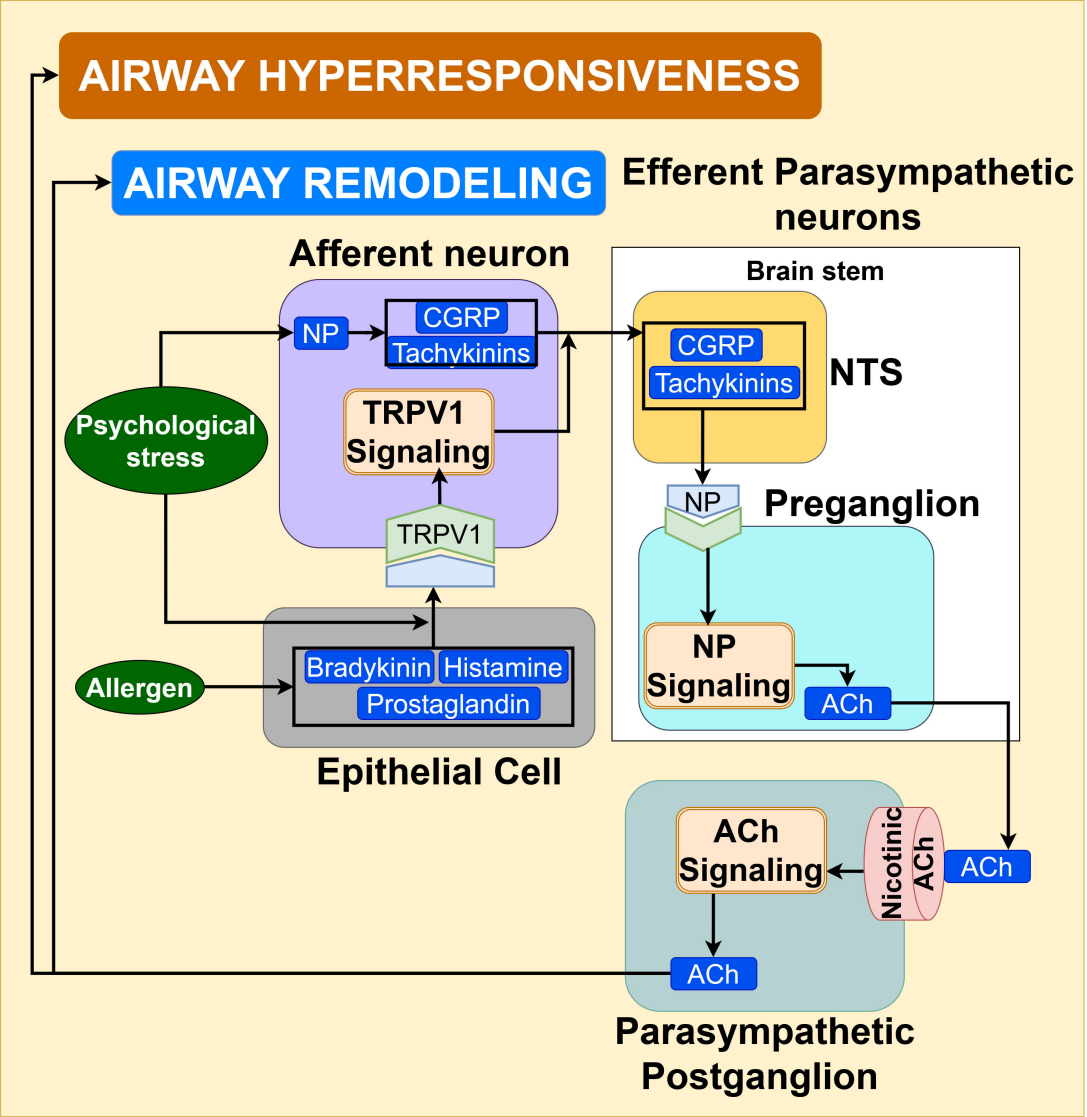
TRPV1 signaling in afferent vagal neurons under the influence of psychological stress triggers the efferent parasympathetic ACh signaling, contributing to airway hyperresponsiveness and airway remodeling, thus exacerbating the asthma pathogenesis.

Afferent TRPV1 signaling: Mechanistic evidence indicates that afferent vagal nociceptors, such as transient receptor potential cation channel subfamily V member 1 (TRPV1) innervated in airways, can be activated by inflammatory mediators like histamine, bradykinin, prostaglandins, and inhaled irritants in allergen-exposed asthmatic airways (104, 105). As illustrated in **Figure 10**, the activated TRPV1 nociceptor signaling in vagal afferent C and A-fibers innervating the airway epithelium transmits the signals to NTS in the brainstem, where the C-fiber receptors release neuropeptides such as calcitonin gene-related peptide (CGRP) and tachykinins like Substance P, and neurokinin A (NKA), promoting the parasympathetic reflex (99, 104, 105, 107).

**Figure 11:**
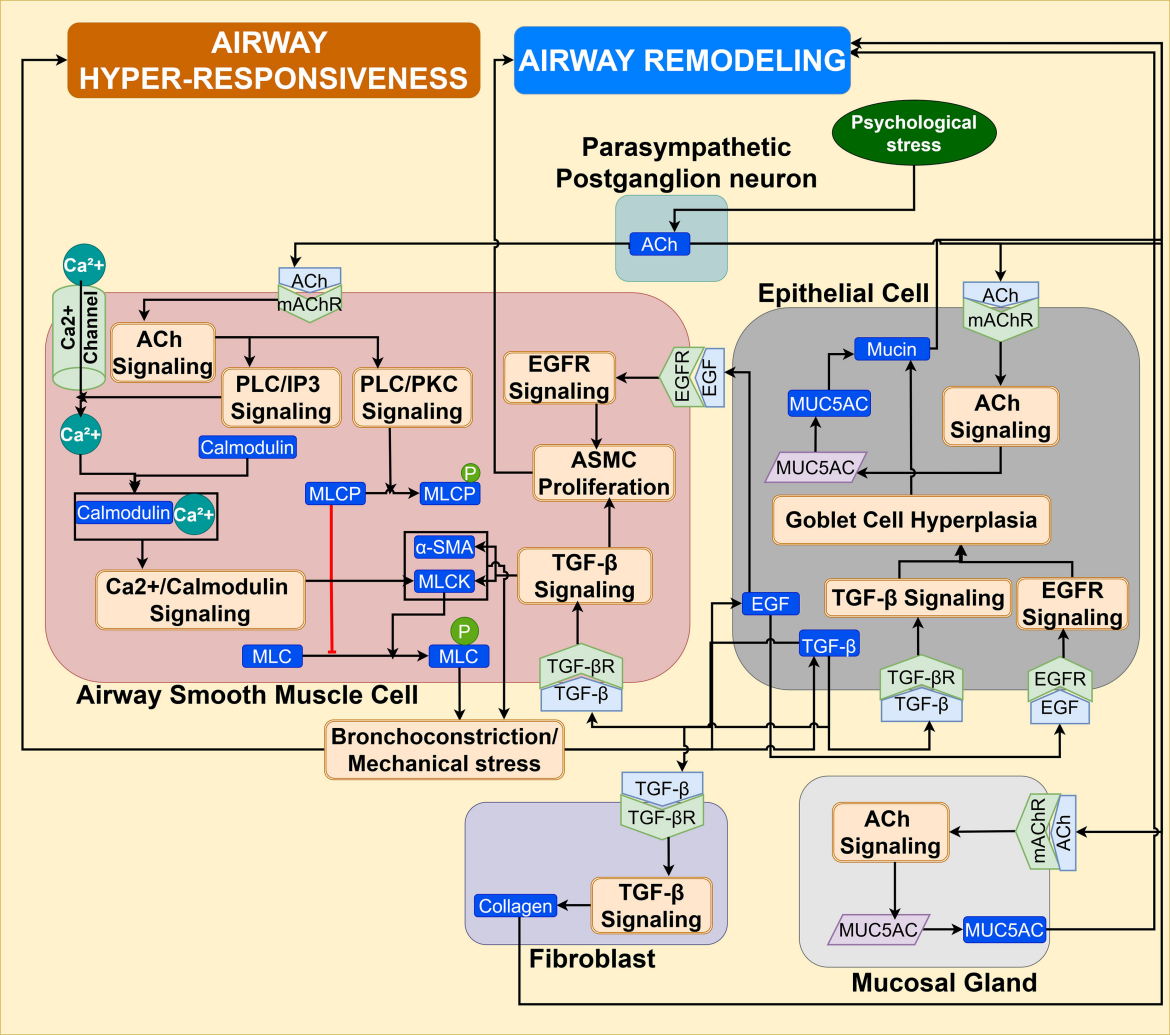
Parasympathetic ACh/PLC/IP3-PKC signaling in ASMC induced by psychological stress aggravates airway hyper-responsiveness. EGF/EGFR signaling in goblet epithelial cells promotes goblet cell hyperplasia and TGF-β signaling in fibroblast and ASMC, promotes ASMC proliferation, myofibroblast differentiation, and augments the airway remodeling in asthmatic airways. The ACh signaling in bronchial epithelial cells and mucosal glands increases the mucus secretion, contributing to airway remodeling and asthma exacerbations.

Efferent parasympathetic acetylcholine and Substance P signaling pathways: Preclinical studies demonstrate that the parasympathetic reflex arc controls airway smooth muscle contraction via a defined neural pathway (103). Neuropeptides released by activated afferent C-fiber receptors in the brainstem interact with receptors on preganglionic neurons, which originate in the brainstem. Activated preganglionic parasympathetic vagal neurons synapse on postganglionic parasympathetic neurons in the airway ganglia, which innervate the airway bronchial wall for neurotransmission, resulting in cholinergic and non-cholinergic parasympathetic pathways, and are schematically represented in **Figure 11** (103, 104, 108). At the synapse, acetylcholine (ACh) released by preganglionic neurons, acts on nicotinic receptors of the postganglionic parasympathetic cholinergic nerve terminals (**Figure 11**), which then release ACh to stimulate the M3 muscarinic acetylcholine receptor (mAChR) on ASMCs, causing bronchoconstriction (103, 105, 108–110).

This binding of neurotransmitter Ach to M3 mAChR, which is a G-protein coupled receptor, leads to activation of the G protein Gq, followed by sequential activation of downstream target phospholipase C (PLC), in ASMC. Activated PLC generates inositol 1,4,5-trisphosphate (IP3) by hydrolyzing phosphoinositol 4,5-bisphosphate (PIP2) and also activates another target protein kinase C. This IP3 promotes intracellular cytoplasmic Ca2+ influx, inducing the formation of calcium-calmodulin complex, which in turn activates myosin light chain kinase (MLCK), and consequent phosphorylation of myosin light chain (MLC), leading to contraction of airway smooth muscle, as shown in **Figure 11**. Activated PKC phosphorylates and inactivates the myosin light chain phosphatase (MLCP), which is a negative regulator of MLCK (109, 111).

Moreover, mechanistic studies show that bronchoconstriction in ASMC causes mechanical stress on the airway epithelium, triggering the release of epithelial growth factors like TGF-β and EGF, which leads to airway remodeling in asthma, as illustrated in **Figure 11** (109, 112). TGF-β signaling in ASMC has been shown to promote ASMC proliferation, as well as increase the expression of contractile proteins such as alpha-SMA and MLCK. While in the airway epithelium, TGF-β signaling enhances goblet cell metaplasia and stimulates collagen secretion in fibroblasts, thereby inducing the differentiation of myofibroblasts (112). Additionally, this mechanical stress is also shown to cause the shedding of EGF ligand, which activates EGFR signaling and promotes ASMC proliferation and goblet cell metaplasia, contributing to airway remodeling (109, 112). Furthermore, ACh also binds to M3 receptor on goblet cells and mucosal gland, stimulating the upregulation of MUC5AC gene expression in goblet cells and triggers the mucus and fluid secretion by submucosal gland, contributing to mucus hypersecretion (**Figure 11**). Thus, airway remodeling promotes exacerbations in allergen-induced asthma (105, 112).

**Figure 12:**
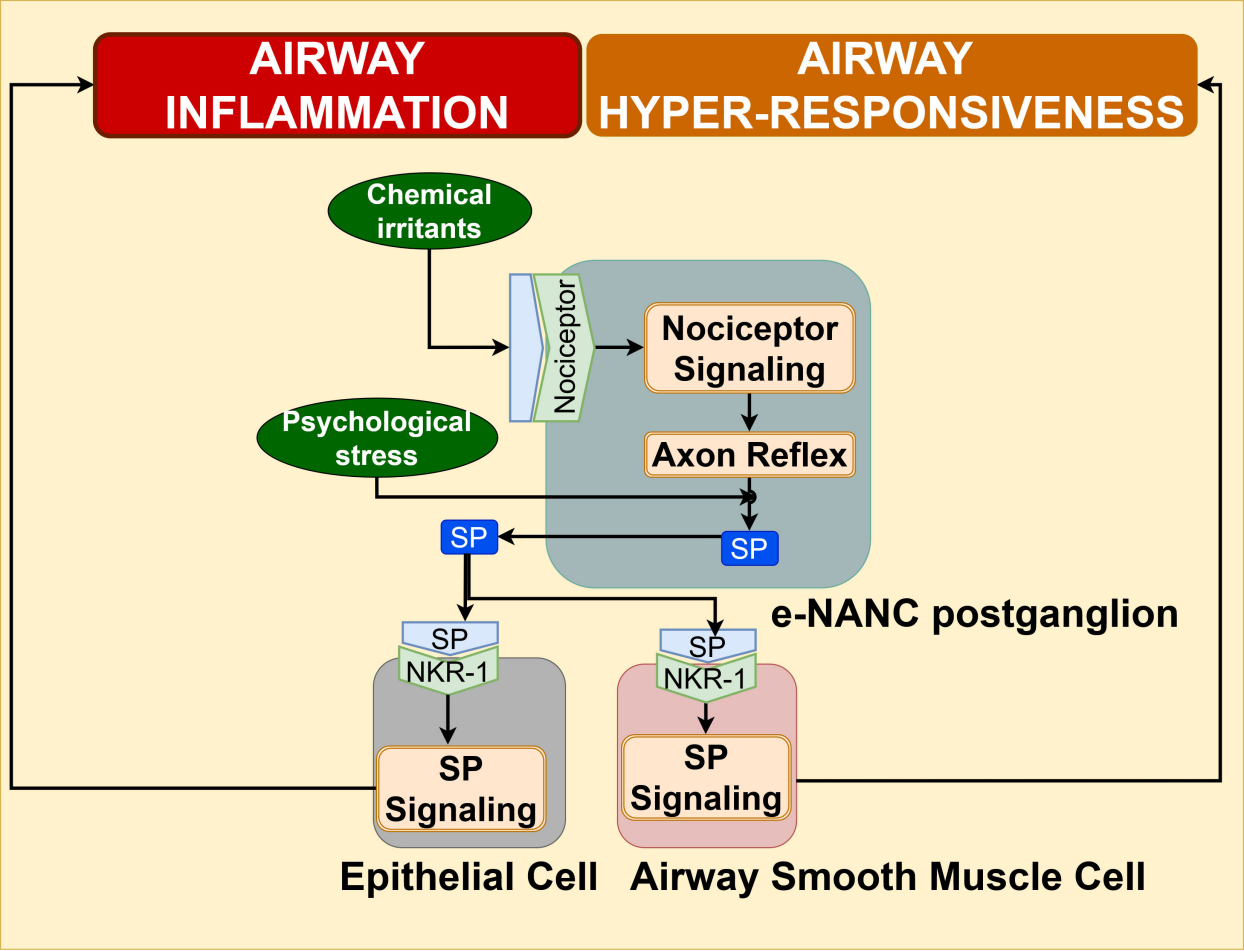
Substance P signaling in airway epithelium and airway smooth muscle cells promotes airway inflammation and airway hyperresponsiveness, respectively, contributing to exacerbation of asthma pathogenesis.

The axon reflex mechanisms triggered by chemical irritants in airways stimulate the nociceptors of excitatory non-cholinergic, non-adrenergic (e-NANC) C-fiber nerve terminals innervating the airway epithelium and smooth muscle layer, which release neuropeptides such as substance P (SP), vasoactive intestinal peptide (VIP), and CGRP (103, 113, 114). These neuropeptides, such as SP and VIP, act on immune cells in the airways to promote Th2 inflammatory response in asthma (114). As illustrated in **Figure 12**, the binding of SP to the neurokinin-1 receptor (NK1R) stimulates the SP/NKR-1 signaling in airway epithelial cells and ASMC, which has been implicated in inflammation and bronchoconstriction, respectively (115–117).

Observational studies link negative emotions in asthma patients with increased vagal cholinergic airway responses and airway hyperresponsiveness (118). Furthermore, M3 mAChR expression on airway epithelium, ASMCs, and glands correlates with MUC5AC upregulation and mucus hypersecretion in asthmatic airways (105, 110). Clinical studies report that psychological stress is associated with elevated release of SP by activated NANC sensory nerves into the airways after exposure to environmental allergens (103, 119).

**Figure 13:**
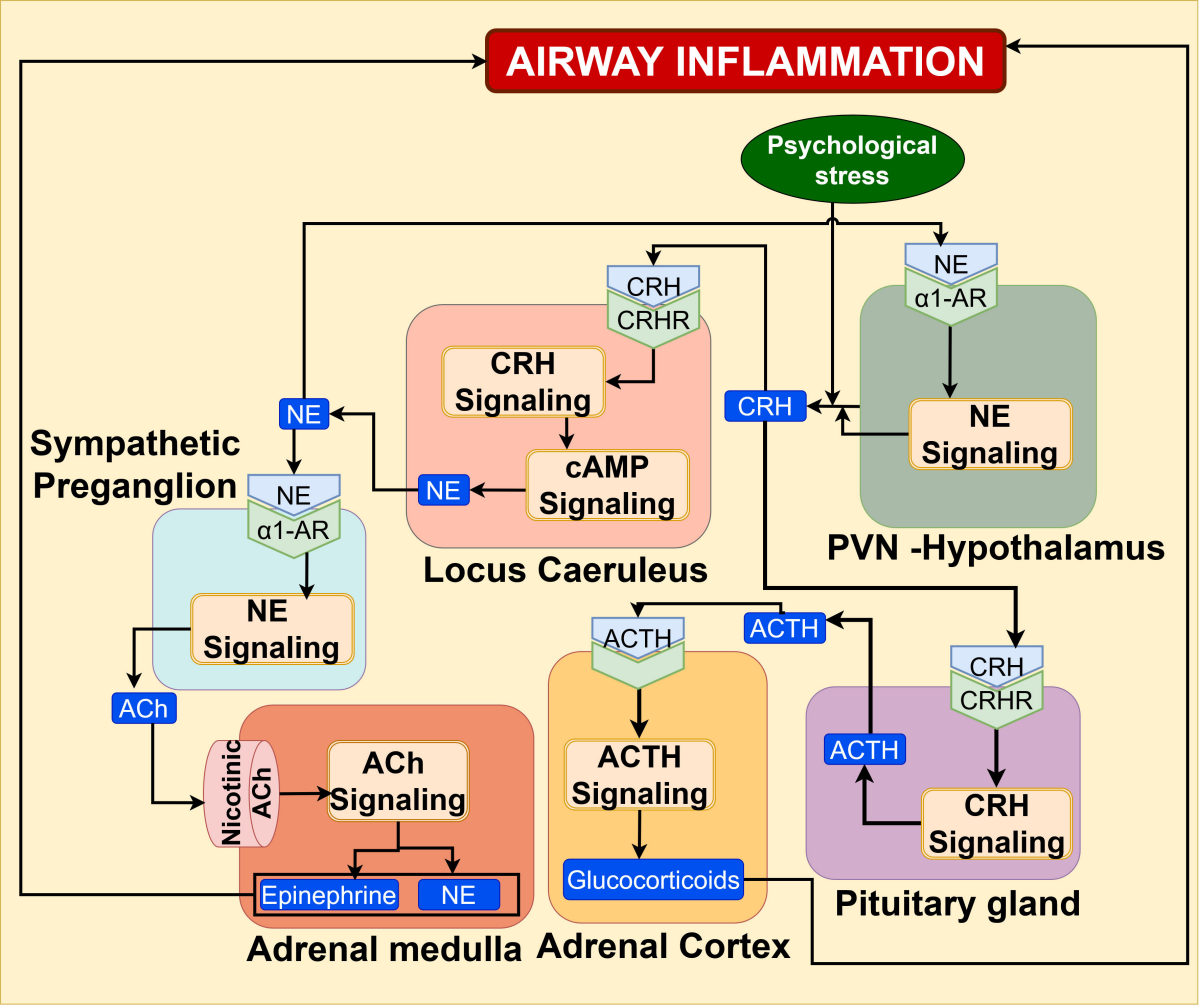
CRH signaling axis under the influence of psychological stress promotes the secretion of GC, NE, and epinephrine, leading to airway inflammation and asthma exacerbation.

CRH signaling axis: Mechanistic evidence from stress-asthma animal models demonstrate that endogenous opioid signaling stimulates the hypothalamic-pituitary-adrenal (HPA) axis and sympathetic and adrenomedullary (SAM) system (120–123). Neuropeptides belonging to the corticotropin-releasing hormone (CRH) family are found to play a major role in regulating the central stress response and the hypothalamic–pituitary–adrenal (HPA) axis. Psychological stress stimulates the secretion of corticotropin-releasing hormone (CRH) neuropeptides by parvocellular CRH neurons in PVN of the hypothalamus (124). Subsequently, the CRH stimulates the CRH1 receptor signaling and its downstream cAMP second messenger system in locus coeruleus (LC) neurons of the brainstem, leading to the release of norepinephrine (NE) (125). This NE triggers the α1-adrenergic receptors (α1-adrenoceptors) signaling in CRH neurons of the hypothalamus, thereby increasing the secretion of CRH, which in turn promotes the adrenocorticotropic hormone (ACTH) secretion from the pituitary gland. This release of circulating ACTH evokes the secretion of glucocorticoid hormones by the cortex of the adrenal gland (124, 126). These stress hormones have been shown to augment airway inflammation in asthma (120–122). LC-derived NE also stimulates sympathetic preganglion nerve terminals synapsing with chromaffin cells of the adrenal medulla to release acetyl choline, which in turn activates nicotinic receptors on the medulla, resulting in epinephrine and norepinephrine secretion (127, 128). The CRH signaling is schematically represented in **Figure 13**, and comprehensive details of SAM/epinephrine signaling are described in the **Supplementary Data**.

Glucocorticoid signaling: Preclinical models demonstrate that chronic psychological stress dysregulates the HPA axis, elevating systemic glucocorticoids (GC) (34, 122, 129). These glucocorticoid hormones, like cortisol, regulate the immune response by binding to an intracellular glucocorticoid receptor (GR) that is expressed in immune cells in lymphoid organs (34, 126). The binding of GC to the cytoplasmic GR induces nuclear translocation of the GC-GR complex, where the GR binds to glucocorticoid response elements in the promoter region of DNA, initiating the transcription of GC-responsive anti-inflammatory genes. This GR is also known to interact with other transcription factors like AP-1 and NF-kB, where the GR exerts an inhibitory effect on NF-kB, resulting in downregulation of proinflammatory genes under normal physiological conditions (130).

**Figure 14:**
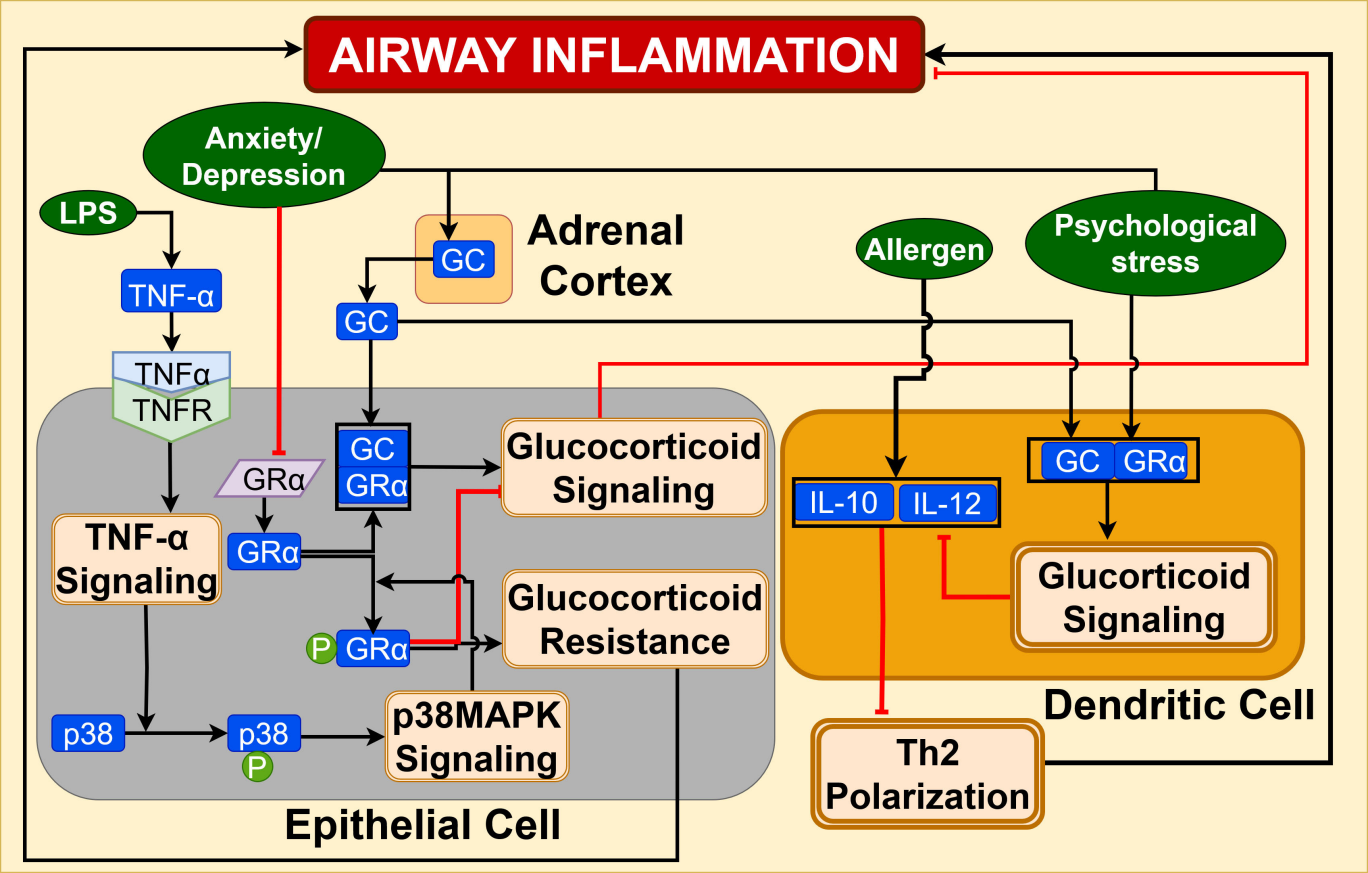
Glucocorticoid signaling in bronchial epithelium is inhibited by psychological stress, causing glucocorticoid resistance and augmentation of airway inflammation. GC signaling in dendritic cells promotes the Th2-mediated inflammation in airways, contributing to asthma exacerbations.

Findings from preclinical investigations using combined ovalbumin-induced and social disruption stress asthmatic mouse models have shown that anxiety or depression can promote increased activation of the HPA axis and glucocorticoid insensitivity, which augment airway inflammation and failure of glucocorticoid therapy in asthma. Depression has been shown to downregulate the expression of GRα as well as inhibit its DNA binding activity through the activation of p38MAPK signaling in bronchial epithelial cells (131–133). As shown in **Figure 14**, increased levels of cytokines like TNFα activate p38 signaling that induces phosphorylation of GRα at serine residue 226 in the cytoplasm, leading to defective nuclear translocation (131, 132). This diminishes the inhibitory effect on anti-inflammatory activities of endogenous glucocorticoid and promotes increased expression of Th2 cytokines like IL-4 and IL-5, contributing to glucocorticoid resistance and exacerbation of airway inflammation in asthma (130, 131).

Mechanistic evidences show that elevated glucocorticoids under stress conditions promote Th2 inflammation in the lungs by inducing Th1/Th2 imbalance and suppressing regulatory T cell development, impairing immune tolerance (38, 134). Kawano et al. demonstrate that the psychological stress-induced glucocorticoid signaling inhibits the growth of pDC-derived Treg cells by decreasing the quantity of plasmacytoid dendritic cells (pDCs) in bronchial lymph nodes (BLNs), and that leads to impaired immunological tolerance in a model of restraint stress-exposed allergen-tolerant mice (134). Moreover, it has been shown that glucocorticoid signaling in dendritic cells can suppress IL-12 and IL-10 production in response to stress. This impairs the respiratory tolerance to airway hyperresponsiveness upon antigen exposure, resulting in the suppression of Th1 and Treg polarization. The downregulation of IL-10 and IL-12, which induces IFNϒ cytokines, leads to enhanced Th2 polarization and amplifies the allergic inflammatory response. This is characterized by eosinophil accumulation and increased level of IL-13 expression in the lung tissue of stressed mice, as depicted in **Figure 14** (122, 134, 135). Therefore, this stress-induced glucocorticoid-dependent skewing of T immune cells towards Th2 inflammatory response increased the susceptibility to allergic asthma, contributing to exacerbations in asthma (37, 122).

Clinical studies report decreased GR mRNA expression strongly correlating with asthma exacerbations in stressed asthmatic children. This indicates an impairment of GC signaling that diminishes the anti-inflammatory properties of GC, contributing to GC resistance (130, 136). Observational studies report higher prevalence of depression symptoms in asthma patients compared to healthy controls (131).

**Figure 15:**
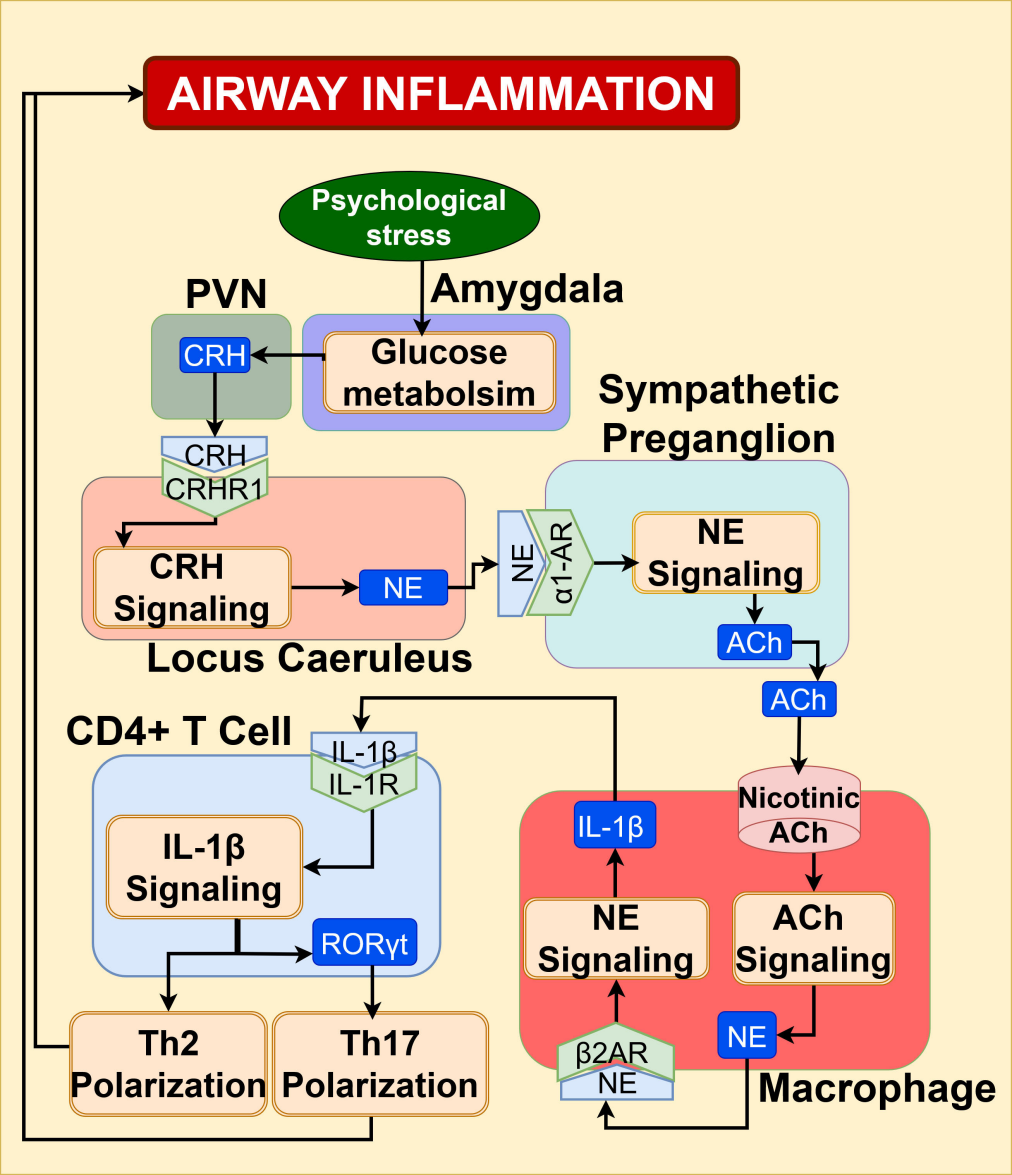
NE/IL-1β signaling augments the Th2 and Th17 airway inflammation, contributing to asthma exacerbation.

NE/IL-1 β signaling: Psychological stress plays a critical role in the activation of brain regions like the amygdala, which has been identified as a neural driver of stress response (100, 137). Mechanistic studies demonstrate that social stress-induced amygdala activation drives CRH secretion from the hypothalamic PVN, triggering brainstem LC-NE release and sympathetic signaling. CRH activates brainstem signaling, facilitating the release of NE from sympathetic nerve terminals that innervate various tissues in the body (100, 137, 138). As schematically represented in **Figure 15**, the NE signaling in immature monocytes of bone marrow induces the IL-1β secretion, and these primed monocytes are recruited to airways, thereby increasing IL-1β/IL-1R inflammatory signaling in airways (139). Sato et al. (140) have shown that in allergen-exposed airways, psychological stress enhances the production of IL-1β by macrophages recruited to the airways. This IL-1 beta signaling promotes Th17 development, raising the amount of IL-17, and lowers the number of T regulatory cells, which increases the level of Th2 cytokines, leading to both eosinophilic and neutrophilic inflammation in asthma (140). Bidirectional IL-1β monocyte trafficking sustains brain-lung inflammation (137).

Clinical PET imaging studies show that amygdala glucose metabolism increases during social stress, correlating with airway inflammation markers in asthma patients (137). Rosenkranz et al. (137) propose bidirectional brain-lung crosstalk whereby IL-1β-producing monocytes traffic to the brain, sustaining IL-1β/IL-1R-mediated inflammation implicated in asthma-associated anxiety and mood disorders (137).

#### 3.4.6 Important findings in the recent literature

Role of ILC2 and CD8+ cytotoxic T (Tc) cells in asthma pathogenesis: CD8+cytotoxic T (Tc) cells are associated with exacerbations of asthma pathogenesis triggered by viral infections and are implicated in resistance to corticosteroid therapy (141, 142). In response to antigen-induced expression of airway epithelial cytokine IL-33, the activated Tc cells deviate from the classical type 1 IFN-γ-secretion (Tc1) to type 2 cytokine (Tc2) producing cells phenotype, contributing to the exacerbation of inflammation in asthmatic airways, as illustrated in **Supplementary Figure 9** (142). Animal models showed the polarized Tc2 effector cells produce IL-13, IL-5, and IL-4 Th2 cytokines, which further augment the airway hyperresponsiveness in asthmatic lungs (142, 143).

IL-33 may promote the Tc2 differentiation through increasing the IL-4 production by immune cells (Th cells, dendritic cells, ILC2 cells) residing in asthmatic lung tissue microenvironment, as IL-4 is a key driver for stimulating the GATA3 expression and consequent synthesis of Tc2 cytokines, as shown in **Supplementary Figure 9** (142). Additionally, studies have reported that rhinovirus infection triggers an increased secretion of human epithelial cytokine IL-33, which is also a known mediator of antiviral T cell responses, where the primary IFN-γ cytokine suppresses the type 2 inflammatory response (142, 144). High levels of IL-33 cytokine in BAL samples obtained from asthma patients have been positively correlated with virus-induced asthma exacerbations (144).

IL-23 signaling: Clinical studies used PET neuroimaging during the Trier Social Stress Test followed by whole-lung allergen challenge in adults to show that chronic stress activates the salience network, evidenced by increased glucose metabolism in the amygdala, insula, and dorsal anterior cingulate cortex. This brain activation was associated with elevated stress markers (e.g., salivary cortisol) and correlated with higher IL-23 mRNA levels and exhaled FeNO, reflecting enhanced Th17-mediated and eosinophilic airway inflammation in allergen-exposed individuals (145).

Preclinical studies show that IL-23 is known to enhance the Th17 differentiation and recruitment of the Th2 inflammatory mediator eosinophils to the airways in asthma (146, 147). Thus, the salience network triggered by psychological stress may intensify the immune response in the airways, resulting in a mixed Th2 and Th17 inflammatory response, leading to both eosinophilic and neutrophilic inflammation, respectively, causing the severity of asthma (145).

## 4 Discussion

This study hypothesized that a molecular systems architecture can provide a roadmap that interconnects the exposure to multiple triggering factors and the pathological mechanisms involved in asthma manifestations, contributing to systems-level understanding of asthma pathogenesis. Understanding of asthma pathogenesis is still an evolving process due to the heterogeneous nature of asthma, characterized by multiple asthma phenotypes and endotypes. The integration of psychological stress and CNS components expands our molecular systems architecture, supported by growing evidence of bidirectional brain-lung axis crosstalk driving asthma exacerbation. Unlike conventional pathways focused on crosstalk among airway cell types that promote Th2/Th17 inflammation in asthma pathogenesis, our architecture maps a broader spectrum of crosstalk across airway structural cells, immune cells, CNS neurons, and adrenal components via stress-activated HPA/LC-NE pathways, driving airway inflammation. Thus, positioning psychological stress as a central modulator of pathobiological processes, including inflammation, hyperresponsiveness, and remodeling, our architecture study may facilitate the mapping of holistic intervention targets that address both environmental and psychosocial triggers for improved disease management.

The results from this system architecture study provide: A multi-layered visual map of biomolecular interactions among the airway and CNS microenvironment, an understanding of the complex crosstalk in both microenvironments, potential targets for drug development, and a framework to develop computational models providing quantitative predictions. One of the results from this study is a multi-layered molecular systems architecture to visualize molecular interactions within and across the cells of the airway microenvironment. These molecular interactions – as shown in **Figure 4** – occur across the airway epithelial cell, airway smooth muscle cell, endothelial cell, pericyte, fibroblast, central and peripheral neurons innervate the airways, and lung resident immune cells such as dendritic cell, microglia, macrophage, CD4+ T cell, Th1 cell, Th2 cell, Th17 cell, Treg cell, B cell, mast cell, ILC2 cell, CD8+ T cell, neutrophil and eosinophil. This visual map can be used to identify how perturbations to a molecular species, e.g., protein, receptor, promoter, gene, mRNA, etc., in one cell may impact the biological functions in a neighboring microenvironmental cell, culminating in inhibiting or activating a biological process such as airway inflammation, airway hyperresponsiveness, and airway remodeling leading to asthma pathogenesis. To make this molecular systems architecture more accessible to the scientific community, there is an effort underway to provide web-based access, wherein viewers may interact to navigate the multiple layers of the architecture. Such a web-based environment will, in the future, enable the scientific community to provide feedback to update the existing version of the architecture to reflect advances in ongoing research, especially by integrating the genetic polymorphisms and their associated environmental triggers, which may facilitate the discovery of newer diagnostic, prognostic, and therapeutic markers for effective management of asthma.

In addition to a visual multi-layered map of biomolecular interactions provided by the molecular systems architecture, the architecture has also revealed the crosstalk across the thirty-one different cells in the lung and CNS microenvironment. Environmental pollutants trigger the crosstalk across airway epithelial cells, fibroblasts, and inflammatory cells; the architecture identified the following three crosstalk signaling pathways: NOX signaling, TLR signaling, and TNF-α signaling. Among airway epithelial cells, ASMC, dendritic cells, and inflammatory cells, the architecture identified the following four crosstalk signaling pathways: IL-4 Signaling, IFN-γ/IL-12 signaling, IL-6 and TGF-β signaling, and TGF-β and IL-2 signaling. Among the crosstalk between epithelial cells, smooth muscle cells, fibroblasts, neutrophils, and eosinophils, the architecture identified the following three crosstalk signaling pathways: EGFR signaling, TGF-β1 signaling, and VEGF signaling. The crosstalk across T Cells, airway epithelial cells, eosinophils, and ASMCs identified the following two crosstalk signaling pathways: IL-13/IL-17 signaling and NO signaling.

Psychological stress exacerbates asthma via established neural and humoral pathways, evidenced by preclinical animal models and clinical studies. Combined with allergen exposure, it triggers crosstalk among airway structural cells, immune cells, central and peripheral neurons, and adrenal components. The molecular systems architecture developed herein identifies nine crosstalk signaling pathways: Eotaxin-1/CCR3 signaling, afferent TRPV1 signaling, efferent ACh signaling, and Substance P signaling, CRH signaling, NE/IL-1β signaling, epinephrine signaling, glucocorticoid signaling, and IL-23 signaling. These insights provide an integrative whole systems understanding of asthma pathogenesis. These established mechanisms align with clinical observations: chronic stress, anxiety, and depression promote bronchoconstriction, inflammation, poor control, exacerbations, and glucocorticoid resistance. Human studies offer primarily correlational evidence like salience network activation linking to increased exacerbations, airway inflammation markers (e.g. FeNO, IL-23), and mixed Th2/Th17 responses, often without direct causal demonstration in patients. the architecture offers a predictive framework for how perturbations in one compartment (e.g. stress-induced CNS activation) may propagate to airway processes, highlighting targets for intervention while noting areas (e.g. full salience network-Th17 priming) requiring further mechanistic validation. The architecture provides a predictive framework for how stress-induced CNS perturbations propagate to airway pathology, identifying intervention targets while flagging needs for further mechanistic validation.

### 4.1 Potential targets for drug development

The molecular systems architecture of asthma identifies candidate molecular targets that warrant further experimental validation for potential drug development. This architecture identifies several candidate targets across airway and CNS cell types that may modulate biological processes of airway inflammation, airway hyper-responsiveness, and airway remodeling, subject to further in silico analyses, ex vivo studies, and ultimately clinical validation.

These potential targets are summarized in **Table 1** and categorized according to one of the three biological processes and mapped in the molecular systems architecture schematics in **Figure 4**. A total of 32 molecular targets were mapped within the molecular pathways involved in airway inflammation, airway hyperresponsiveness, and airway remodeling across airway microenvironmental cells and neurons of the nervous system. Among these thirty-two, twenty-three targets were mapped in cells, including epithelial cells, T helper effector cells, dendritic cells, mast cells, afferent vagal neurons, macrophages, e-NANC neurons, and eosinophils, that are implicated in the molecular pathways involved in airway inflammation. One target was mapped in microglia that is involved in neuroinflammation. Seven targets were mapped in various cells, such as epithelial cells, ASMC, mast cells, ILC2 cells, macrophages, and e-NANC neurons that are involved in molecular pathways mediating airway hyper-responsiveness or bronchoconstriction. Finally, eight targets mapped in epithelial cells, ASMCs, macrophages, mucosal glands, and Th17 cells were found to be associated with molecular mechanisms involved in airway remodeling.

**Table 1:** Molecular targets in asthma pathways.

S.No	Molecular target	Cell type	Biological process	Reference
1.	IL-33	Airway epithelial cell	Airway inflammation, Airway hyperresponsiveness	(148)
2.	IL-13	Th2 cell, Mast cell, ILC2 cell	Airway hyper-responsiviness	(14, 20, 93)
3.	IL-5	Th2 cell, Mast cell, ILC2 cell	Airway inflammation	(20, 93)
4.	IL1RL1	Mast cell	Airway inflammation	(148)
5.	IL-6	Dendritic cell, Epithelial cell, Fibroblast	Airway inflammation	(74)
6.	IL-17	Th17 cell	Airway remodeling, Airway inflammation, Airway hyper-responsivity	(63, 75, 149)
7.	CCR6	Treg cell	Airway inflammation	(150)
8.	IL-10	Treg cell	Airway inflammation	(68)
9.	EGFR	Bronchial epithelial cell	Airway inflammation; Airway remodeling	(151)
10.	YKL-40	Bronchial epithelial cell	Airway inflammation; Airway remodeling	(84)
11.	TGF-β1	Bronchial epithelial cell and eosinophil	Airway remodeling	(85)
12.	NOX4	Airway epithelial cell, Smooth muscle cell	Airway inflammation; Airway remodeling	(152)
13.	MMP-9	Bronchial epithelial cell	Airway remodeling	(153)
14.	VEGF	Airway epithelial cell, Macrophages, and Smooth muscle cell	Airway remodeling	(89)
15.	RhoA	Airway smooth muscle cell	Airway hyperresponsiveness	(93)
16.	ROCK	Airway smooth muscle cell	Airway hyperresponsiveness	(93)
17.	iNOS	Airway epithelial cells, alveolar macrophages	Airway hyperresponsiveness	(94)
18.	sGC	Airway epithelial cells, Smooth muscle cells	Airway hyperresponsiveness	(94)
19.	Glutathione	Airway epithelial cells	Airway inflammation	(49, 154)
20.	SOD	Bronchial epithelial cells	Airway inflammation	(49, 154)
21.	Thioredoxin	Bronchial epithelial cells	Airway inflammation	(49, 154)
22.	Nrf-2	Airway epithelial cells	Airway inflammation	(49, 154)
23.	CCR3	Microglia	Neuronal inflammation	(23, 102)
24.	SP	eNANC neurons	Airway hyperresponsiveness, Airway inflammation	(105, 115, 117)
25.	CGRP	eNANC neurons	Airway inflammation	(114)
26.	VIP	eNANC neurons	Airway inflammation	(114)
27.	NKR-1	Airway smooth Muscle cell, Bronchial epithelial cell,	Airway hyperresponsiveness, Airway inflammation	(116)
28.	mAChR M3	Airway smooth Muscle cell, Bronchial epithelial cell, Mucosal gland	Airway remodeling	(109, 112)
29.	TRPV1	Vagal afferent neurons	Airway inflammation	(99)
30.	β2AR	Bone marrow-derived dendritic cells	Airway inflammation	(120)
31.	P38MAPK	Bronchial epithelial cell	Airway inflammation	(131, 132)
32.	IL-1β	Macrophage	Airway inflammation	(137)

IL, Interleukin; IL1RL1 Interleukin 1 receptor-like 1; CCR6, CCR6-C-C chemokine receptor type 6; EGFR, Epidermal growth factor receptor; YKL-40-chitinase-3-like protein 1; TGF-β1, Transforming Growth Factor beta-1; NOX4, NADPH oxidase; MMP-9, Matrix metalloproteinase-9; VEGF, Vascular endothelial growth factor; ROCK, Rho-associated coiled-coil containing protein kinase; RhoA, Ras homolog family member A; iNOS, Inducible nitric oxide synthase; sGC, Soluble guanylate cyclase; SOD, Superoxide dismutase; Nrf2, Nuclear factor erythroid 2-related factor 2; CCR3, CC Chemokine Receptor 3; SP, Substance P; NKR-1, Neurokinin-1 receptor; CGRP, Calcitonin gene-related peptide; VIP, vasoactive intestinal peptide; TRPV1, Transient receptor potential cation channel subfamily V member 1; mAChR, muscarinic acetylcholine receptor; IL-1β, Interleukin 1 beta; β2AR, Beta-2-adrenergic receptor; p38 Mitogen-Activated Protein Kinase.

Moreover, this architecture study has also provided valuable insight into the link between psychological stress and asthma exacerbations. Psychological stress can influence the environmental exposures on asthma by disrupting the homeostasis of the lung-brain axis mediated by neuroimmune interactions, which alters the brain function as well as augments the Th2 and Th17 inflammation in airways and airway hyperresponsiveness. The architecture study has provided a visual map of the relationship between psychological stress and the biphasic regulation of the molecular interactions mediating lung-brain crosstalk in asthma. Airway inflammatory response in asthma triggers the release of neurotransmitters from the central and peripheral nervous systems, which are key players in worsening asthma symptoms like bronchoconstriction. Hence, the molecular architecture of asthma developed herein can be a powerful tool to identify molecular targets that are involved in pathological pathways regulated by neurotransmitters and airway inflammatory mediators, in response to environmental pollutants and psychological stress.

Current drug development aims to identify a single compound that affects a particular target to inhibit or delay asthma progression. This systems architecture may inform multi-target strategies via in silico modeling, but requires experimental validation to assess the key role of underlying neuro-immune pathways in asthma exacerbations and related neurodegenerative disorders. For example, the molecular systems architecture may be used to identify how activating or inhibiting more than one target using multi-combination therapies can affect multiple biological processes implicated in asthma pathogenesis. In addition, the visual map could provide insights into how targeting one or more mechanisms in the airway microenvironment affects the biological process in either a positive or negative therapeutic outcome. Such insights may also include understanding how a therapeutic that has a beneficial effect on a target in a particular cell, may interact with another target in a different cell of the airway microenvironment to lead to adverse effects on treatment outcome.

### 4.2 Limitations

Although this molecular systems architecture provides a comprehensive visual map of asthma pathogenesis, integrating interactions across 31 cell types and 25 key pathways in both airway and CNS microenvironments, several important limitations remain. The architecture is based on a systematic curation of peer-reviewed literature published between 2008 and 2025 using the CytoSolve^®^/PRISMA-guided process, which may underrepresent very recent advances such as single-cell RNA sequencing (scRNA-seq) insights. Asthma’s heterogeneity (e.g., Th2-high/low endotypes) limits strong causal links between environmental triggers and specific endotypes, as the same trigger can drive both eosinophilic and neutrophilic inflammation. Though this architecture maps triggers to endotypes, definitive associations remain elusive due to intricate underlying biology, where triggers initiate or exacerbate disease but do not dictate endotype. Identified targets thus require endotype-stratified biomarker validation to enable precision therapies across diverse immune responses. As a static interactome, it lacks dynamic simulations or wet-lab validation, an aspect that is currently being explored for future work. Murine stress-axis data may not fully translate to humans due to differences in microbiome composition, glucocorticoid responses, and other confounders. These limitations highlight the need for future enhancements, including AI-assisted literature curation, multi-omics integration for parameterization, dynamic CytoSolve-based modeling, and targeted clinical validation studies to strengthen translational applicability.

## 5 Conclusion

This architecture offers a framework for integrative computational models to prioritize **Table 1** candidates, similar to validated precedents, with experimental calibration needed for translation.”

In previous work, a molecular systems architecture of pancreatic cancer was successfully used to develop computational models that predicted dosages of a two-drug combination that was allowed by the U.S. Food and Drug Administration (FDA) (155). Additionally, in silico models based on a molecular systems architecture of osteoarthritis were used to derive an optimal combination of two bioflavonoids targeting molecular pathways of pain and inflammation (156). These computational models of airway microenvironment may be used to answer research questions, such as, of the targets identified by the molecular systems architecture in **Table 1**, which ones will prove more effective in reducing airway inflammation/hyper-responsiveness/remodeling. Moreover, these in silico models may be used to provide quantitative predictions for applications such as efficacy and safety of single and multi-combination therapeutics, and determination of optimal dosages.

## Data Availability

The original contributions presented in the study are included in the article/**Supplementary Material**. Further inquiries can be directed to the corresponding author.
