# Supplementary material for: A molecular systems architecture of asthma
Source: Front Immunol. 2026 Apr 24;17:1788984. doi: 10.3389/fimmu.2026.1788984 (PMC13154161; doi:10.3389/fimmu.2026.1788984)
Supplement: Supplementary file 1 [file DataSheet1.docx]

**Supplementary Information**

**A molecular systems architecture of asthma**

V.A. Shiva Ayyadurai^1, 2*^, Yamuna Manoharan^1, 2,^, and Prabhakar Deonikar^1, 2^

^1^Systems Biology Group, CytoSolve Research Division, CytoSolve, Inc., Cambridge, MA

^2^Open Science Institute, International Center for Integrative Systems, Cambridge, MA

^3^Departments of Biological Engineering and Mechanical Engineering, Massachusetts Institute of Technology, Cambridge, MA

**Table of Contents**

Supplementary Data

Supplementary Figures

Supplementary Tables

Supplementary References

Supplementary Glossary

**1. Supplementary Data**

**1. NOX signaling:** NOX isoforms DUOX1 and DUOX2 have been identified as major producers of ROS (H_2_O_2_) in alveolar epithelial cells and are found to play a key role in airway inflammation, triggered by environmental allergens and infections (Nadeem et al., 2015). Increased level of DUOX2 expression have been observed in people with asthma, viral infections and smokers. Epithelial cell receptors involved in recognition of pathogens including PAR-2 and TLRs 2, 3, 4, 5 and 6 have been implicated in the activation of DUOX1/2 and promotion of airway inflammation (Koff et al., 2008; Nadeem et al., 2015). Allergic asthma mouse model studies by Nadheem et al, demonstrates the triggering of PAR-2 activation by cockroach allergens, resulting in the upregulation of DUOX-2, which induces the H_2_O_2_ production and consequent mucus hypersecretion and epithelial apoptosis, driving the airway remodeling pathogenesis of asthma. The DUOX-2/ROS signaling in airway epithelial cells has been shown to induce mitochondrial dysfunction that characterized by increased expression of proapoptotic proteins BAX and Caspase-3 (Nadeem et al., 2015). Additionally, Ryu et al, had also reported the LPS triggered activation of TLR-4 signaling induced activation of DUOX-2 resulting in CCL20 secretion by bronchial epithelial cells. The CCL20 chemokine recruits dendritic cells, promoting the Th2 allergic inflammatory response in asthmatic airways (Ryu et al., 2013).

Moreover, previous studies have shown that DUOX1/2 and NOX-4 mediated ROS signaling facilitates the upregulation of MUC5AC gene expression in airway epithelial cells (Hyun et al., 2008; Nadeem et al., 2015; van der Vliet et al., 2018). Data from Hyun et al. indicates that the activated EGFR-ERK1 MAP kinase signaling in response to exogenous ROS, can stimulate NOX-4 expression and subsequent ROS production can upregulate MUC5AC expression in goblet epithelial cells (Hyun et al., 2008). In vitro studies by Chan et al, demonstrates enhanced expression of NOX2 and NOX4 in the human bronchial epithelial cells, triggered by TLR-4 signaling, in response to aeroallergen HDM. This NOX2/4/ROS signaling promotes the production of cellular nitrogen species (RNS) and ROS that damage DNA causing double stranded DNA breaks and mitochondrial oxidative stress leading to airway epithelial cell injury (Chan et al., 2017). Diminished expression of antioxidant gene Nrf-2 were also observed in the presence of HDM triggered NOX2/4 signaling and that contributes to suppression of antioxidant response mediated by SOD2, catalase and glutathione (Chan et al., 2017).

Glutathione peroxidase (GPx) has peroxyl scavenging mechanism, while catalyzing the oxidation of GSH to glutathione disulfide (GSSG), thereby removing the lipid peroxidation products and hydrogen peroxide, which are generated by the infiltrated leukocytes in the lung (Ammar et al., 2022). Data from Ammar et al, reports an increased level of SOD activity and decreased glutathione level and GPx activity in plasma from asthmatic patients, as predictive biomarkers of severe asthma GPx has peroxyl scavenging mechanism, while catalyzing the oxidation of GSH to glutathione disulfide (GSSG), thereby removing the lipid peroxidation products and hydrogen peroxide, which are generated by the infiltrated leukocytes in the lung. (Ammar et al., 2022). Data from Ammar et al, reports an increased level of SOD activity and decreased glutathione level and GPx activity in plasma from asthmatic patients, as predictive biomarkers of severe asthma (Ammar et al., 2022). In vivo and ex vivo studies have shown the significant association between bronchial epithelial overexpression of NOX4 and epithelial ciliary dysfunction contributing to neutrophilic asthma that accompanied by persistent inflammation and increased susceptibility to bacterial infection (Wan et al., 2016).

Animal studies by Koffman et al. and Habibovic et al. have displayed the pathological role of Duox-1 contributing to neutrophilic inflammation and airway hyperresponsiveness via the persistent activation of EGFR signaling in HDM induced asthmatic airway epithelium (Habibovic et al., 2016; Koff et al., 2008; van der Vliet et al., 2018). HDM induced activated DUOX-1 has been shown to promote epithelial secretion of IL-33, which subsequently interacts with ILC2 cells via the ST2 receptor, inducing the expression of Th2 cytokines (IL-13, IL-5, IL-4) and EGF ligand amphiregulin (Habibovic et al., 2016; van der Vliet et al., 2018). The IL-13 signaling in airway epithelial cells stimulates the DUOX-1 expression and IL-8 secretion, followed by neutrophil recruitment, thereby contributing to neutrophilic inflammation (Habibovic et al., 2016). The ILC2 derived amphiregulin ligand of EGFR, which is known to have a physiological role in EGFR mediated epithelial repair response, were also found to induce sustained EGFR activation, due to its high affinity binding to the epithelial EGFR, in airways (Habibovic et al., 2016). Additionally the ROS produced by DUOX-1 has also been implicated in cysteine oxidation of EGFR, enhancing the ligand induced EGFR phosphorylation in epithelial cells. Thus, DUOX-1, induced dysregulated EGFR signaling has been shown to promote goblet cell metaplasia and consequent mucus hypersecretion and airway hyperresponsiveness (Habibovic et al., 2016; Koff et al., 2008; van der Vliet et al., 2018). Interactions of NOX across epithelial cells, dendritic cells, ICL2 cells, and neutrophils are illustrated in Figure 5 in the main text.

**2. TLR Signaling:** Toll like receptors (TLRs) are well known transmembrane receptors that located on the plasma membrane or endosomal compartments of the cell (Zakeri & Russo, 2018). Evidence from previous literatures have shown the TLR expression in lung epithelial cells, alveolar macrophages or dendritic cells and other immune cells, where the TLR recognizes antigenic ligands such as pathogen-associated molecular patterns (PAMPs) and host-derived damage-associated molecular patterns (DAMPs) (Zuo et al., 2015). Interaction of TLRs with its pathogenic ligands activates the TLR signaling that transduced via two different pathways including MyD88 (myeloid differentiation primary response 88) and TRIF (Toll/IL-1 receptor-domain containing adapter-inducing interferon-β) pathways (Zakeri & Russo, 2018). TLR signaling is illustrated in Supplementary Figure 1A.

Among several TLR receptors, data from multiple studies have identified that TLR2 and TLR4 receptors recognizing the gram positive bacteria and LPS (lipopolysaccharides) of gram negative bacteria and house dust mite allergen, respectively, play a major role in asthma pathogenesis (Zuo et al., 2015). Animal studies demonstrates that LPS-induced TLR4/MYD88 signaling pathway in pulmonary dendritic cells, via the activation of its downstream targets including IRAK4 and NF-kB, increases the expression of co-stimulatory molecules (CD86 and MHC class II peptide) and cytokine TNF-α, promoting the Th2 inflammatory response (Piggott et al., 2005; Zakeri & Russo, 2018). Hsia et al. have shown that the TLR4/TRIF signaling via the activation of interferon stimulating factor (IRF) 3, stimulates the upregulation of CD40 receptor on pulmonary DC, accompanied by increased level of IL-6 cytokine, thereby promoting the Th17 neutrophilic inflammation in asthma (Hsia et al., 2015; Mishra et al., 2018).

Animal asthma model studies have reported that inhalation of LPS containing allergens stimulates TLR4 signaling in airway epithelium, promoting VEGF (vascular endothelial growth factor) secretion, which activates VEGFR2 signaling pathway in matured DCs that had migrated to regional lung lymph node, promoting the upregulation of Th17 polarizing cytokine IL-6 that drives the differentiation of primed naïve T cells into Th17 cells mediating airway inflammation (Y.-S. Kim et al., 2009). Furthermore, TLR4 signaling in airway epithelial cells triggered by exposure to HDM has been shown to promote allergic asthma characterized by type 2 inflammatory responses. (Hammad et al., 2009; Komlósi et al., 2022). Apart from airway immune responses, the TLR4 signaling in epithelial cells also induces the production of inflammatory cytokines like GM-CSF (granulocyte-macrophage colony-stimulating factor ), IL-33, IL-25 and chemokines (CCL20 and CXCL1) mediating the DC migration on airway epithelium, upon exposure to LPS or HDM (Hammad et al., 2009; Komlósi et al., 2022; Zakeri & Russo, 2018). The GM-CSF secretion by airway epithelium activates DCs, which primes the CD4+ T cells in the lymph node leading to Th2 effector cells differentiation, initiating the development of allergic asthma pathogenesis (Mishra et al., 2018; Rayees et al., 2020; Zakeri & Russo, 2018).

**3. TNF-α Signaling:** TNF-α is a proinflammatory cytokine and plays a critical role in the host innate immune response against microbial infection. TNF-α is primarily produced by macrophages, in response to activation of its Toll-like receptors, which detects common bacterial antigen LPS (lipopolysaccharides) or allergen ovalbumin (Brightling et al., 2008). Lung resident inflammatory cells including neutrophils, mast cells, eosinophils, dendritic cells, B cells, CD4+ cells, and structural cells such as fibroblasts, epithelial and smooth muscle cells produce TNF-α (Brightling et al., 2008; Cazzola & Polosa, 2006). TNF-α plays a critical role in the host innate immune response against microbial infection. TNF-α induces the production of ROS, which in turn stimulates MAPK/ERK/p38 signaling leading to expression of proinflammatory mediators like IL1β, IL6, IL8, TNF-α, and adhesion molecules such as MCP-1 and eotaxin-1, leading to inflammatory cells migration (Brightling et al., 2008; Ma et al., 2016). TNF-α signaling also induced mucus production via NFkB dependent MUC5AC gene transactivation in airway epithelial cells (Lora et al., 2005). TNF-α is associated with airway remodeling through stimulating fibroblasts proliferation, myofibroblast differentiation and promoting TGF-β expression (Brightling et al., 2008). TNF-α signaling pathways across various cells of airway microenvironment are shown in Supplementary Figure 1B.

**4. IL-4 Signaling:**  The Th2 immune response help in the elimination of extracellular pathogens (Awasthi et al., 2008; Pelaia et al., 2015). Clinically, GATA3 expression in lung tissue has been positively correlated with allergic airway inflammation and airway hyperresponsiveness (S. H. Kim et al., 2014). Reports indicate that Th2 cytokines IL-4 and IL-13 interacts through the IL-4R⍺/IL-13R⍺1 receptor complex on B lymphocytes, stimulating the immunoglobulin class switching from IgG to IgE, thereby increases the production of IgE. Binding of IgE to FcεRI receptor on mast cells leads to mast cell activation, promoting mast cell degranulation and secretion of eosinophil recruiting cytokine IL-5 (Pelaia et al., 2015; Suraya et al., 2021). These cytokines, in synergistic action with epithelial derived chemokines including eotaxin (CCL11, CCL24) and RANTES (CCL5), have been shown to bind to the CCR3 receptor on eosinophils, facilitating eosinophil infiltration in the airways, which contributes to airway epithelial damage and promotes airway hyperresponsiveness (Barnes, 2017; Pelaia et al., 2015). Additionally, the cytokine IL-5 signaling via binding to IL-5Rα on eosinophils and has been shown to regulate eosinophil maturation, activation and survival (Suraya et al., 2021). Moreover, studies have identified that antigenic stimulation of IgE on mast cells triggers the process of mast cell degranulation by releasing inflammatory mediators such as histamine, prostaglandin, leukotriene, IL-13, and tumor necrosis factor TNF-α (Suraya et al., 2021) . IL-4 signaling is illustrated in Figure 6 in the main text.

**5. IL-33 Signaling:** Among epithelial cytokines, IL-33 found to be predominantly expressed by bronchial epithelial cells in response to exposure to pollutants like diesel exhaust particle (Cevhertas et al., 2020). Several studies have reported IL-33 as potent mast cell activator, stimulating the production of cytokine IL-13 and protease tryptase by mast cells (Yoshida et al., 2021). Tryptase has been identified as activator of PAR2 (protease-activated receptor-2) signaling in airway epithelial cells, inducing the secretion of other epithelial cytokine TSLP (Yoshida et al., 2021). Studies have shown that TSLP on synergistic stimulation with other cytokines (IL-1β, TNF-α), specifically enhances IL-5 and IL-13 expression in mast cells (Allakhverdi et al., 2007). Moreover, the level of PAR-2 were found to be increased in the epithelium of asthma patients, initiating the type 2 inflammation (Kouzaki et al., 2009; Yoshida et al., 2021). Thus, the IL-33/PAR-2 signaling, as seen in Supplementary Figure 2A facilitates the crosstalk between airway epithelial and mast cells promoting type 2 inflammation that associated with airway hyperresponsiveness (Yoshida et al., 2021).

IL-33/ST2 signaling in other immune cells such as ILC2, basophils and natural killer cells promotes Th2 inflammatory response through the secretion of type 2 cytokines (IL-4, IL-5, IL-13) by these cells (Duchesne et al., 2022). Moreover, evidences have shown that IL-33 as potent inducer of bronchoconstriction via the expansion of ILC-2 cells producing the IL-13 cytokine, which plays a major role in airway hyperresponsiveness (Barlow et al., 2013). It has been reported that fungal and influenza viral infections can trigger IL-33 dependent signaling in ILC2 cells (Barlow et al., 2013).

**6. IL-12 and IFNγ Signaling:**  Interferon γ (IFNγ) is a well-known Th1 effector cytokine and accumulating evidences indicates that IFNγ plays a dual role in asthma, where it can repress Th2 activation, attenuating the eosinophilic airway inflammation and augments the neutrophilic airway inflammation (Teixeira et al., 2005; Zhu et al., 2020a). Previous literatures have shown that the Th1 polarizing cytokine IL-12, initially secreted by both macrophages and dendritic cells, in response to intracellular bacterial pathogens. Findings demonstrates that the LPS induced IL-12 production by macrophages and dendritic cells can be through the activation of IFN-γ or TNF receptor and antigen triggered TLR receptors, respectively (Flesch et al., 1995; Vroman et al., 2015). Studies also indicate that IL-12 signaling can induce the IFN-γ production by macrophages and dendritic cells, where IFN-γ signaling acts in an autocrine manner, generates a positive feedback loop resulting in augmentation of IL-12 secretion (Kaiko et al., 2008).

As shown in Supplementary Figure 2B, macrophage or dendritic cell derived IL-12 cytokine has been identified to act via IL-12 receptor and its downstream signaling target STAT4 in naïve T cell, driving the expression of IFN-γ, Which in turn mediates the expression of transcription factor T-bet via the activation of STAT-1 (Luckheeram et al., 2012; Teixeira et al., 2005). Binding of IFN-γ to its receptor complex on CD4+ T cell has been shown to stimulate the receptor associated JAK1/2 via auto phosphorylation, followed by STAT-1 activation and that binds to the T-bet promoter sequence known as GAS (gamma activated site), promoting the T-bet expression for further Th1 differentiation (Kaiko et al., 2008; Teixeira et al., 2005). Furthermore, the activated T-bet has been identified to maintain the expression of IL-12 receptor (IL-12Rβ2), thereby stabilizing the production of Th1 effector cytokine IFN-γ and maintenance of Th1 immune response (Luckheeram et al., 2012; Teixeira et al., 2005). Additionally, studies have identified that IFN-γ signaling can exert inhibitory effects on the expression of Th2 cytokine IL-4 via the upregulation of IFN-γ responsive gene IRF, which binds to the IL-4 promoter sites and acts as transcriptional repressors (Elser et al., 2002; Teixeira et al., 2005).

Clinical studies have demonstrated elevated levels of BALF IFN-gamma cytokine and CXCL10 chemokine in human subjects with severe asthma, which have been shown to be strongly associated with airway hyperresponsiveness and corticoid resistance (Gauthier et al., 2017; Raundhal et al., 2015). Moreover, data from Raundhal et al, also indicate an inverse correlation between IFN-gamma expression and SLPI (secretory leukocyte protease inhibitor), which is generally known to inhibit allergen-triggered leukocyte serine proteases such as mast cell-derived tryptase and chymase (Raundhal et al., 2015). Animal studies using the mouse severe asthma model display the IFN-gamma-induced downregulation of SLPI in airway epithelial cells, contributing to activation of mast cell proteases resulting in inflammation (Lambrecht et al., 2019; Raundhal et al., 2015). IFN-gamma and corticosteroids activate STAT1 and the glucocorticoid receptor (GR), respectively, which are recruited to the CXCL10 promoter, thereby stimulating CXCL10 overexpression in monocytes, leading to CXCL10 binds to the CXCR3 receptor expressed by Th1 cells, mast cells, and neutrophils, thus contributing to persistent type 1 inflammation and corticosteroid resistance in severe asthma, as illustrated in Supplementary Figure 2B (Gauthier et al., 2017).

**7. IL-6-TGF-β-IL-17 Signaling:** Antigen-triggered co-stimulatory interactions between dendritic cells and activated CD4+ Tcells, promotes Th17 polarization in the presence of cytokines including IL-1𝛽, IL-6 and TGF-β in airway microenvironment (Pelaia et al., 2015; Vroman et al., 2015). Proinflammatory cytokines IL-6 and TGF-β are produced by the activated DCs (Vroman et al., 2015). Additionally, the lung structural cells including epithelial cells, fibroblast, endothelial cells and effector CD4+ T helper cells have been reported to contribute to the increased level of IL-6 cytokine in airway microenvironment under inflammatory conditions (Sze et al., 2020). Activation of both IL-6 and TGFβ signaling synergistically activates STAT3, which in turn activates transcription factor RORγt, stimulating Th17 cell differentiation and secretion of cytokines such as IL-17A, IL-17F, IL-22, and IL-23R (Awasthi et al., 2008; Newcomb & Peebles, 2013; Vroman et al., 2015). The IL-23 cytokine signaling via IL-23R has been found to stabilize the production of Th17 cytokines like IL-22 in a STAT-3 dependent manner, thereby promoting the Th17 pathogenicity, as seen in Supplementary Figure 2D (McGeachy et al., 2009).

Viruses are one of the primary causes of asthma exacerbations, characterized by neutrophilic inflammation. Exposure to diesel fumes and cigarette smoke is linked to higher serum IL-17 levels in children with asthma and is associated with neutrophilic inflammation (Brandt et al., 2013; Hynes & Hinks, 2020; Siew et al., 2017). It has been reported that IL-17 binds to IL-17R receptor and activates NF-kB, promoting the expression of the mucin gene MUC5B and IL-8 in bronchial epithelial cells. This leads to mucus overproduction and neutrophil recruitment contributing to the airway remodeling and inflammation, respectively, as seen in Supplementary Figure 2D (Fujisawa et al., 2011; Hynes & Hinks, 2020; Newcomb & Peebles, 2013). Moreover, the IL-17A and IL-22 via the activation of the ERK 1/2 pathway signaling can also promote ASMC proliferation, resulting in remodeling of asthmatic airways (Newcomb & Peebles, 2013).

**8.TGF-β-IL-2 Signaling:** Data from studies have shown that lack of naive CD4+ differentiation into Treg cells population augments the Th2 proinflammatory response, promoting asthma exacerbations. T regulatory cells (Tregs) occur in two forms including thymus derived CD4+CD25+ Foxp3+ Tregs cells (nTregs / tTregs) and peripheral Tregs (p Tregs / iTregs) derived from peripheral lymphoid tissue, where naive CD4+Foxp3− T cells differentiates into Tregs, after antigenic stimulation in the presence of cytokine milieu including TGF-β, IL-2, retinoic acids, IL-10 and IFN-γ (Awasthi et al., 2008; Zhang et al., 2022; Zhao & Wang, 2018). TGF-β and IL-2 are the primary cytokines that stimulates the FoxP3 expression in naive CD4+CD25+ cells, driving the differentiation into Foxp3+ pTreg cells, which induces immune tolerance via the secretion of IL-10 and TGF-β (Zhao & Wang, 2018).

TGF-β signaling activates the binding of transcription factor Smad-3 to Foxp3 gene promoter, where it acetylates the H4 histone in the promoter region of Foxp3, contributing to an enhancement of Foxp3 transcription (Zhao & Wang, 2018). Further, the IL-2 is known to signal via CD25 receptor involving the activation of STAT-5 pathway and that accelerates Foxp3 gene expression (Burchill et al., 2007). As illustrated in Supplementary Figure 2E, Tregs are known for its inhibitory effects on allergy mediated inflammations through various mechanisms such as suppression of effector T cells (Th1, Th2, and Th17), eosinophils and inflammatory dendritic cells, followed by an induction of antibody isotype switching from IgE to IgG4 (S. H. Kim et al., 2014). Treg cells have been reported to negatively regulate the differentiation of effector T cells (Th1, Th2), through the competitive binding of its CTLA-4 receptor to B7 molecules (CD80, CD86) that expressed on the antigen presenting dendritic cells (Zhao & Wang, 2018). The CTLA-4 receptor or CD154 has been reported to be constitutively expressed by Foxp3+ T-reg cells and studies demonstrates that CTL-4 captures and degrades the B7 ligands from other activated T cells via trans-endocytosis and consequent suppression of CD28 pathway in T cells (Magee et al., 2012; Walker, 2013).

The IL-10 is a well-known for its pleotropic effects as it can exert anti-inflammatory or immunostimulatory response on different cell types (Chung, 2001). Recent clinical and animal studies shows that IL-10 exherting an anti-inflammatory effect, suppressing the allergen specific immune responses and mainly associated with prevention of chronic inflammation in asthma (Schülke, 2018). Clinically, asthmatic individuals have been reported to have reduced levels of IL-10 secretion from alveolar macrophages and in bronchoalveolar lavage fluid, when compared with non-asthmatic individuals (Coomes et al., 2017). Furthermore, Coomes et al. demonstrated the direct IL-10 signaling in Th2 cells, resulting in increased production of granzyme B, which induces Th2 cell death, leading to amelioration of HDM-induced airway inflammation, as illustrated in Supplementary Figure 2E (Coomes et al., 2017). Recent clinical studies has reported reduced levels of IL-10 in allergic patients upon allergen exposure, attributing to the defects in IL-10-producing T cells, which have been implicated in inflammatory pathogenesis of airway allergy (Coomes et al., 2017). Therapeutic strategies involving the generation of IL-10 producing DCs are being widely studied for its effects on downregulating the allergenic antigen-specific Th2 inflammatory responses (Schülke, 2018).

**9. EGFR Signaling:** Asthmatic airway epithelium is characterized by loss of epithelial integrity and goblet cell hyperplasia promoting mucus hypersecretion that aggravates airway obstruction (Joseph & Tatler, 2022; Tagaya & Tamaoki, 2007). Persistent damage triggered by environmental pollutants and the airway smooth muscle cell contraction exerting mechanical compressive forces on epithelial cells during bronchoconstriction has been shown to augment asthmatic airway epithelial injury (Hough et al., 2020). Several growth factors such as TGF-β, early growth response1 (Egr-1), endothelin 1 and epidermal growth factor receptor (EGFR) that are secreted by injured epithelial cells, facilitates further progression of pathological changes involving airway remodeling in asthma (Hough et al., 2020; Joseph & Tatler, 2022; Tagaya & Tamaoki, 2007).

Mechanical compressive forces and prevalence of high levels of TNF-α derived from neutrophils and eosinophils in asthmatic airways induced the overexpression of EGFR and EGFR ligand HB-EGF (Heparin-bound epidermal growth factor) in injured bronchial epithelial cells. Figure 7 in the main text illustrates that HB-EGF activates EGFR/ERK signaling, which has been shown to promote goblet cell hyperplasia via increasing the number of goblet cells that associated with pathophysiological process of mucus hypersecretion and consequent airway obstruction in asthma (Ha & Rogers, 2016; O’Sullivan et al., 2020; Tschumperlin et al., 2002). Furthermore, the activated EGFR via the stimulation of MEK1/2 and ERK signaling, also shown to induce the expression of chitinase like protein YKL-40, where its increased serum levels in patients with asthma demonstrated significant correlation with airway remodeling and disease severity, as shown in Figure 7 in the main text (Park et al., 2010). It has been reported that YKL-40 can stimulate angiogenesis, smooth muscle cell proliferation and migration, contributing to airway remodeling (Hough et al., 2020; O’Sullivan et al., 2020; Park et al., 2010).

**10. TGF-β1 Signaling**

TGF-β1 cytokine is known to play a major role in airway remodeling and also implicated in airway hyperresponsiveness (Ojiaku et al., 2017). High level of TGF-β1 expression was observed in bronchoalveolar lavage fluid and bronchial biopsies obtained from asthmatic patients, which correlated well with pathological features of subepithelial fibrosis (VIGNOLA et al., 1997). Though studies have reported that TGF-β1 is produced by multiple lung cells including epithelial cells, macrophages, eosinophils, lymphocytes and fibroblasts, among which the TGF-β1 derived from epithelial cells and eosinophils has been found to be major contributor to asthma pathogenesis (Hough et al., 2020; Kariyawasam & Robinson, 2007; Ojiaku et al., 2017). Findings indicate that ROS generated in asthmatic airway epithelium triggers the production of TGF-β1 by epithelial cells and eosinophils, which in turn increases the level of ROS via the activation of NOX-4 resulting in fibroblast activation, myofibroblast differentiation and consequent airway remodeling (K. Liu et al., 2022). TGF-β1 signals via both Smad dependent and Smad independent pathways, mediating the airway remodeling and hyperresponsiveness. In Smad dependent pathway, the activated TGF-β1 is known to bind to the TGF-β type II receptor (TβR-II), which subsequently phosphorylates TGF-β type I receptor (TβR-I), followed by phosphorylation of Smad2 and Smad3 (Smad2/3) transcription factors that translocate to nucleus, promoting the transcription of target genes (Doherty & Broide, 2007; Ojiaku et al., 2017). Studies have identified that TGF-β1 stimulates proliferation of airway goblet cells, smooth muscle cells and fibroblast, while inducing apoptosis in epithelial cells (G. Chen & Khalil, 2006; Ojiaku et al., 2017).

TGF-β1 drove the proliferation of ASMCs via the activation of ERK and p38 dependent signaling pathways and induction of NOX-4 expression in airway smooth muscle cells (G. Chen & Khalil, 2006; Sturrock et al., 2007). TGF-β1 also induced NOX4 expression via Smad 3 dependent signaling, which upregulated the production of ROS and that in turn phosphorylated and activated ERK1/2, resulting in ASMC proliferation, as illustrated in Supplementary Figure 3A. Additionally, TGF-β1/NOX-4 induced ROS was found to increase the phosphorylation of cell cycle regulator protein RB and that subsequently released E2F transcription factor that drives the cell cycle to prolonged S phase, allowing the protein synthesis, thereby promoting the smooth muscle cell hypertrophy (Sturrock et al., 2007). Thus, ASMC hyperplasia and hypertrophy induces morphological alterations in airways that are characterized by thickening of the airway wall, followed by fibrosis in the reticular basement membrane (Savin et al., 2023; Sturrock et al., 2007).

As shown in Supplementary Figure 3A, activated TGF-β1/Smad2/3 signaling cascade in fibroblast involves the interaction between receptor Smads (Smad 2/3) and Smad 4, followed by translocation to the nucleus, where the Smad complex transactivates the expression of fibrogenic genes leading to myofibroblast differentiation (Royce et al., 2012). Clinical, experimental animal model and genome wide association (GWAS) studies have demonstrated the increase in the level of Smad2 and Smad3, suggesting elevated level of TGF-β1 in asthmatic lungs (Le et al., 2007; Moffatt et al., 2010; Royce et al., 2012; Sagara et al., 2002). Differentiated myofibroblast affects subepithelial fibrosis via the secretion of ECM proteins like collagen I, collagen III and fibronectin, which promotes the thickening of reticular basement membrane and showing correlation with airway hyperresponsiveness (Hsieh et al., 2023; Savin et al., 2023). Moreover, findings reports that TGF-β1 signaling inhibits the production of MMPs by epithelial and inflammatory cells and promotes the synthesis of TIMPs (tissue inhibitors of metalloproteinase) by epithelial cells (Boxall et al., 2006).

**11. VEGF Signaling:** Vascular epidermal growth factor (VEGF) is a mitogenic peptide that well known for its potent induction of endothelial cell migration, proliferation and tubule formation (K. S. Lee et al., 2008; Simcock et al., 2008). Functionally, the VEGF plays a major role in the regulation of angiogenesis and vasculogenesis (Chetta et al., 2005). Studies have reported the role of VEGF in the enhancement of vascular permeability, allowing the migration of inflammatory cells and mediators from plasma into the airways (K. S. Lee et al., 2008). Numerous clinical studies have reported the high level of VEGF expression in the lung tissue biopsies and bronchoalveolar lavage fluid (BALF) from asthmatic patients and have shown direct correlation with disease severity (K. S. Lee et al., 2008; Simcock et al., 2008; Türkeli et al., 2021). Data from previous clinical research studies involving bronchial mucosal biopsies obtained from asthma patients, have shown the VEGF expression in macrophages, CD34+ cells, epithelial cells, airway smooth muscle cells, eosinophils and mast cells (Chetta et al., 2005; Hoshino et al., 2001; Simcock et al., 2008). Murine asthmatic model studies by Lee et al. demonstrated the role of VEGF in subepithelial fibrosis via inducing the expression of profibrotic cytokine TGF-β through the activation of PI3K/AKT signaling pathway in lung epithelial cells, promoting the peribronchial accumulation of collagen (I, III, and V), fibronectin, hyaluronan, laminin, tenascin and periostin, within the lamina reticularis of the airways, resulting in thickening of the basement membrane (K. S. Lee et al., 2008; Savin et al., 2023).

As illustrated in Supplementary Figure 3B, activation of PI3K/AKT pathway stimulates the transcription factor HIF1α, which transactivates and increases the VEGF expression, thus suggesting a positive feedback loop between HIF-1α and VEGF exists in allergic airway disease (K. S. Lee et al., 2008). Chetta et al. further reported the correlation between VEGF overexpression and increased level of subepithelial basement membrane thickness in asthmatic patients (Chetta et al., 2005). Recent study by Turkeli et al. demonstrates the anti-VEGF and TNF-α treatment can restore the epithelial barrier integrity, by increasing the levels of E‑cadherin and β‑catenin (Türkeli et al., 2021). VEGF induced diminished expression of E‑cadherin and β‑catenin have been shown to promote disruption of tight junction barrier integrity in airway epithelium leading to increased access of inhaled allergens to antigen presenting cells in the subepithelial basement membrane and consequent exacerbation of Th2 inflammatory response (Türkeli et al., 2021).

*In vitro* angiogenesis assay studies by Simcock et al. demonstrated the increased level of secretion of proangiogenic factors including VEGF, angiopoietin (Ang-1) and angiogenin by airway smooth muscle cells cultured from asthmatic patients. Among these ASMC derived proangiogenic factors, the VEGF signaling in endothelial cells has shown to be a critical mediator in driving the neovascularization process, which has been found to induce vascular endothelial cell proliferation, migration and tubule formation, as shown in Supplementary Figure 3B (Simcock et al., 2008). Findings also indicate that Ang-1 acts synergistically with VEGF, via the Tie-2 surface receptor on endothelial cells to promote stabilization of nascent endothelial vessel network via stimulating the interactions between endothelial cells and surrounding pericytes that support the vessel network (Simcock et al., 2008) . Furthermore, Simcock et al. have demonstrated the TGF-β induced production of VEGF in airway smooth muscle cells (Simcock et al., 2008).

**12. IL-13/IL-17 Signaling:** Increased level of intracellular calcium influx (Ca2+) from the extracellular environment via the voltage-dependent calcium channel and from the sarcoplasmic reticulum in smooth muscle cells, plays a pivotal role in stimulating the contraction of ASM (Kudo et al., 2013). Findings reveals that the intracellular Ca2+ in ASM, forms a complex with serine threonine kinase protein calmodulin and that activates MLC kinase (MLCK), which in turn phosphorylates MLC (Myosin light chain). The p-MLC stimulates of interactions between actin and myosin, triggering the ASM contraction, which is regulated by changes in the level of cytosolic calcium, where at low calcium levels, the Myosin phosphatase dephosphorylates and inactivates the p-MLC, as seen in Supplementary Figure 4 (Erle & Sheppard, 2014; Kudo et al., 2013).

Animal and in vitro studies have shown increased expression of GTPase RhoA protein induced by T cell inflammatory mediators IL-13 and IL-17 in airway smooth muscle cells (Chiba et al., 2009; Kudo et al., 2013). The Rho A has been found to negatively regulate MLC phosphatase, through the activation of its downstream target Rho associated, coiled-coil containing protein kinase (ROCK), which phosphorylates the myosin phosphatase target subunit 1 (MYPT-1) and consequent upregulation of p-MLC leading to increased ASM contraction, inducing airway hyper responsiveness, as illustrated in Supplementary Figure 4 (Chiba et al., 2009; Erle & Sheppard, 2014).

**13. NO Signaling:** Constitutive expression of iNOS occur in bronchial epithelium to maintain the epithelial barrier integrity, protect against pathogen entry and regulate the mucociliary function (Mattila & Thomas, 2014). Additionally, as illustrated in Figure 8 in the main text, under inflammatory conditions, the LPS or other pro-inflammatory cytokines such as interleukin 1 (IL-1β), interferon (IFN-γ), and tumor necrosis factor (TNF-α), have been shown to stimulate the transcription factors including NFkB and AP-1 to activate iNOS gene expression in airway epithelial cells (Bayarri et al., 2021; Donnelly & Barnes, 2002). Studies have reported the role of multiple signaling pathways mediating the iNOS expression lung epithelial cell such as (i) Receptors including TLR-4, INF-γ receptor, TNFα receptor or IL-1βR have been found to activate NFkB pathway in response to the recognition of cytokine or pathogenic stimuli (ii) INF-γ receptor, TNFα receptor or IL-1βR has been shown to induce AP-1 mediated transactivation of iNOS expression via the stimulation of MAPK/ERK/p38 signaling pathways; (iii) Receptors including INF-γ receptor, TNFα receptor or IL-1βR has also been found to stimulate JAK2/STAT1 pathway, inducing the iNOS expression (Ganster et al., 2001; Jia et al., 2016; Kristof et al., 2001).

Increased expression of iNOS in asthmatic airway epithelium and elevated levels of exhaled NO have been associated with bronchial wall thickening in asthmatic patients (Bayarri et al., 2021; Donnelly & Barnes, 2002). Moreover, IL-13 secreted by eosinophils was shown to augment the iNOS synthesis in both epithelial cells and alveolar macrophages residing in asthmatic airways, thus exacerbating the NO production (Bayarri et al., 2021; Roos et al., 2014). At cellular level, the iNOS enzyme is known to for its catalytic activity in mediating the production of NO and citrulline from the amino acid L-arginine (Bayarri et al., 2021).

As shown in Figure 8 in the main text, epithelial NO diffuses to airway smooth muscle cells and activates lung sGC by binding to reduced ferrous (Fe2+) of the heme group at N-terminal domain of the β subunit of sGC in both epithelial and airway smooth muscle cells (Papapetropoulos et al., 2006). Binding of NO triggers the catalytic activity of the enzyme at its C-terminus resulting in cGMP synthesis from GTP and high level of intracellular cGMP contributes to smooth muscle relaxation via lowering the level of intracellular ca2+ (Papapetropoulos et al., 2006). Among the intracellular downstream targets of cGMP, evidences indicate that cGMP stimulates PKG1 (cGMP dependent Protein Kinase 1), which in turn controls vascular tone by activating cytosolic proteins via phosphorylation, thereby regulating the level of intracellular calcium in vascular smooth muscle cells (Du & Roberts, 2019) . Ex-vivo studies by London et al, reports diminished expression of sGC protein in primary airway smooth muscle cells obtained from patient with severe asthma and consequent decrease in cGMP production leading to increased bronchoconstriction in asthma (London et al., 2018).

Moreover, studies have also reported that NOX-4 derived superoxide, reacts with NO resulting in the generation of peroxynitrite, attributing to the reduced availability of NO and inhibition of the binding to NO to sGC, as seen in Figure 8 in the main text, leading to the suppression of NO-sGC-cGMP-PKG pathway in the asthmatic airway epithelium, which promotes ciliary dysfunction (Bayarri et al., 2021; Price & Sisson, 2019). Physiologically, this NO-sGC-cGMP-PKG pathway has been shown to regulate epithelial sodium channels (ENaC), which plays a critical role in acceleration of wound healing, improving the ciliary beating and alveolar fluid clearance, thus identified to be a potential therapeutic target in the treatment of asthma (Bayarri et al., 2021; Y. Liu et al., 2016; Nie et al., 2009).

TGF-β has been shown to mediate downregulation of GCα1 subunit expression via the activation of MEK/ERK pathway in pulmonary artery smooth muscle cells (PASMCs) obtained from newborn mouse with lung injury (Du & Roberts, 2019). Furthermore, studies suggest that inducing the cGMP production can inhibit TGF-β signaling, that upregulated in asthmatic airway epithelium through the activation of PKGs, which has been shown to inhibit non canonical TGF-β/MEK/ERK signaling pathway, thereby impeding the EMT process involved in airway remodeling pathogenesis of asthma (Bayarri et al., 2021).

**14. MMP-9 Signaling:** Matrixmetalloproteinsases belongs to the family of zinc dependent endopeptidases that catalyzes the proteolytic degradation of ECM components, attributing to the structural changes of airways (Hough et al., 2020; Royce et al., 2012). MMP-9 expression is observed in bronchial epithelium biopsies obtained from patients with asthma and found to play a pivotal role in mediating the eosinophil transmigration across the basement membrane, via degrading the basement membrane components (Han et al., 2003; Okada et al., 1997). Additionally, MMPs (MMP-2, MMP-9, MMP-13, and MMP-14) has been shown to activate latent growth factors like TGF-β and VEGF that embedded within the extracellular matrix (ECM) of airway basement membrane (Al-Alawi et al., 2014; Mott & Werb, 2004). As shown in Supplementary Figure 5, MMP-9 was found to cleave the latent associated peptide (LAP), which non-covalently interacts with TGF-β cytokine forming a latent complex. LAP mediates the covalent attachment of the latent TGF-β complex to a latent TGF-β binding protein (LTBP), facilitating the TGF-β sequestration in ECM (Mott & Werb, 2004). MMP-9 also implicated in the proteolytic cleavage of ECM heparan sulfate proteoglycans and release of bound VEGF from the ECM (Mott & Werb, 2004).

**15. SAM-epinephrine signaling axis:** Preclinical studies show that psychological stress stimulates the release of endogenous opioids or neuropeptides in brain neurons that act on μ-opioid receptors (MORs) that widely expressed by neurons of the central nervous system and immune cells (Miyasaka et al., 2018; Okuyama et al., 2012; Rosenkranz et al., 2022). These opioids like endorphin is found to activate MOR signaling in nonadrenergic neurons of the brain stem, specifically locus coeruleus and adrenergic neurons of central nervous system, which activate the sympathetic nervous system, resulting in the release of epinephrine and norepinephrine from adrenal medulla (E. Chen & Miller, 2007; Miyasaka et al., 2018; Okuyama et al., 2012).

Mechanistic evidence indicates that stress-induced opioid/MOR signaling in the CNS has been shown to cause a significant reduction in regulatory T cells and an increase in the IFN-γ/IL-4 ratio in bronchial lymph node (BLN) cells obtained from stressed mice stimulated with allergen, indicative of a Th2 immune response. Thus, MOR is a central mediator of stress-induced augmentation of allergic airway inflammation, by modulating the allergen stimulated immune response, contributing to asthma exacerbation (Okuyama et al., 2010, 2012). Moreover, Kim et al. (2010) demonstrated that sympathetic activation and release of stress hormones like epinephrine can activate β2-adrenergic receptor (β2AR) signaling and enhance the priming of LPS stimulated bone marrow-derived dendritic cells (BMDCs) and modulate the naive CD4+ polarization to Th2 and Th17 phenotype (B.-J. Kim & Jones, 2010). This activated epinephrine/β2-adrenergic signaling has been shown to increase the antigen-stimulated TLR signaling-dependent expression of costimulatory molecules such as MHCII, CD80, and CD86, and cytokines like IL-23, IL-4 as well as downregulating the IL-12p40 cytokine expression in activated BMDC. Epinephrine primed dendritic cells promote the Th17 and Th2 differentiation through the the IL-23 dependent IL-17 and IL-4-dependent Th2 cytokine production, respectively, in CD4+ T cells (B.-J. Kim & Jones, 2010). Therefore, antigen-primed dendritic cells promote polarization to the dominant Th2/Th17 phenotype, upon exposure to stress-derived epinephrine released from sympathetic nervous endings in bone marrow lymphoid tissue (B.-J. Kim & Jones, 2010). Schematic representation of epinephrine signaling is given in Supplementary Figure 7.

Clinical studies indicate that endogenous opioids like beta-endorphin and endomorphin are found to have a strong association with asthma exacerbation (Miyasaka et al., 2018).

**16.** **Macrophage polarization**: Macrophages are widely distributed throughout the lung microenvironment and two types of macrophages that reside in lung tissue include interstitial macrophages (IMs) and alveolar macrophages (AM) (J.-W. Lee et al., 2021). AMs are in close contact with alveolar epithelial cells, while IMs are found in the parenchyma that lies between the alveolar epithelium and microvascular endothelium (J.-W. Lee et al., 2021). Exposure to allergens, such as cockroach allergen, has been shown to activate immune cells and lung epithelial cells in asthmatics, leading to the production of inflammatory cytokines. These inflammatory mediators have been shown to facilitate the recruitment of blood monocytes, which increases the population of monocyte derived alveolar macrophages (Mo-AM) and promote primary polarization of these AMs into classically activated (M1) and alternatively activated (M2) macrophages (Saradna et al., 2018; Zhu et al., 2020a).

Inflammatory cytokines such as IFN-γ and LPS derived from bacteria that present in airway inflammatory microenvironment in asthma, have been shown to promote M1 macrophage phenotype, as seen in Supplementary Figure 5 (Ross et al., 2021). M1 macrophage phenotype is characterized by the overexpression of MHC class II molecules, CD80, CD86, iNOS, proinflammatory cytokines (TNF-α, IL-1β, IL-6, IL-12, IL-23, IL-27) and chemokines (CCL2) (Ross et al., 2021; Saradna et al., 2018; Zhu et al., 2020b). It has been shown that cytokines generated by M1 macrophages, such as TNFα and IL-12, may stimulate Th1 cells, and Th17 cells can be activated by IL-23 and IL-27, which can promote non-allergic inflammation (Saradna et al., 2018; Zhu et al., 2020b). According to reports from experimental asthmatic mice model studies, exposure to farm dust extract increased the population of M1 macrophages accompanied with higher expression of Th1 and Th17 cells that associated with nonallergic lung inflammation. While exposure to house dust mite extract (HDM) showed accumulation of M2 macrophages with a predominant Th2 cell response (Jiang & Zhu, 2016; Saradna et al., 2018).

Alternatively, the Th2 cytokines such as IL-4 and IL-13 are well known primary inducers of alveolar macrophage differentiation into alternate activated macrophages (AAM) (Ross et al., 2021; Zhu et al., 2020b). Additionally, the IL-33 released from damaged airway epithelium caused by allergens, found to induce M2 polarization (Ross et al., 2021). AAMs have been found to express high level of various phenotypic markers such as macrophage mannose receptor 1 (MRC1/CD206), arginase (Arg-1), chitinase like protein (Ym1/2) and resistin like molecule alpha (FIZZ1) (Saradna et al., 2018; Zhu et al., 2020a). Findings also suggest that M2 macrophages play a major role in allergic asthma pathogenesis (Abdelaziz et al., 2020; Ross et al., 2021; Zhu et al., 2020a). Recent evidences have identified 4 subtypes of AAM (M2) such as M2a, M2b, M2c and M2d, among which, the M2a macrophages has been shown to promote eosinophilic inflammation in asthma (Abdelaziz et al., 2020). Research investigations have reported that M2a macrophages express high level of IL-13, MRC1, transglutaminase (TGM2) and chemokines, such as CCL-17, CCL-18, CCL-22, and CCL-24, which promote the Th2 cell activation and consequently eosinophil infiltration into the lungs (Ross et al., 2021; Saradna et al., 2018). M2a macrophages with higher expression of both MRC1 and MHC-II has been observed in BALFs from asthma patients (Girodet et al., 2016). It has been demonstrated that chemokines like CCL-17, CCL-22 and CCL-24 mediate Th2 cell recruitment and TGM2 promote inflammation by potentiating the enzymatic activity of phospholipase A2 (PLA2) I. This PLA2 has been shown to modulate the production of cysteinyl leukotrienes by mast cells and eosinophils (Abdelaziz et al., 2020).


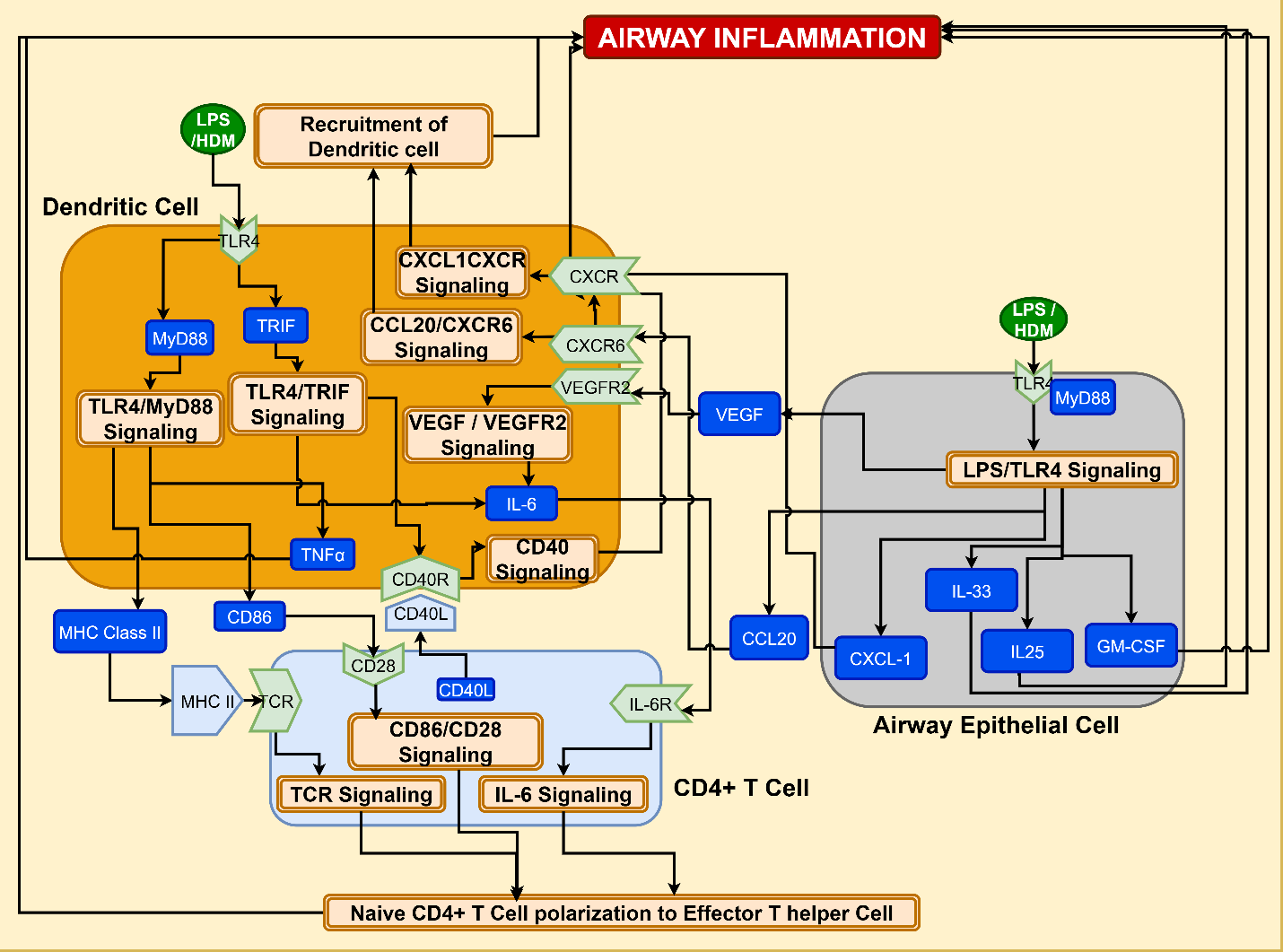
**2. Supplementary Figures**

**Supplementary Figure 1A.** TLR signaling in airway epithelium triggered by aeroallergens or LPS leads to activation of dendritic cells and naïve CD4+ T cell polarization to T helper cells, promoting the airway inflammation.


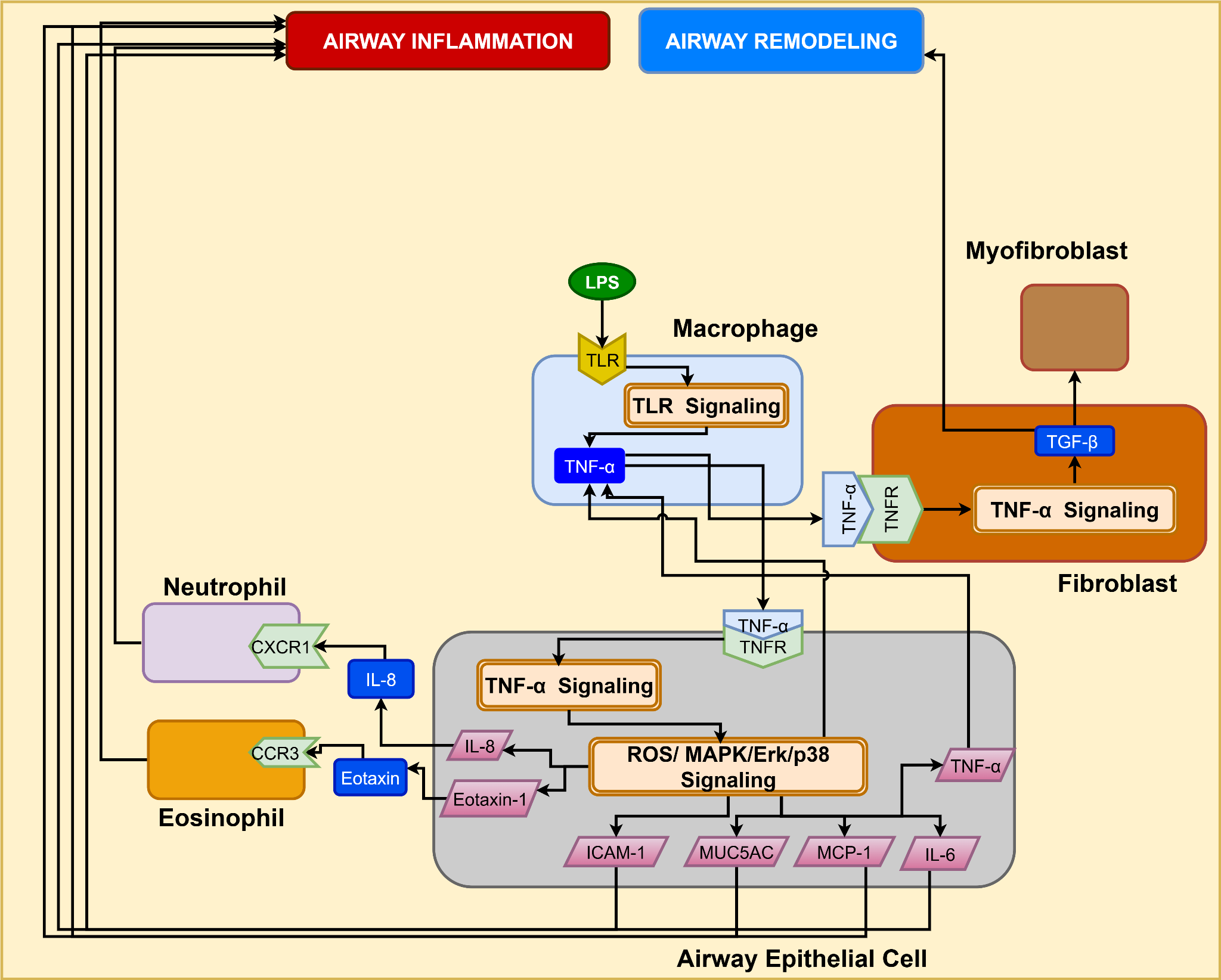


**Supplementary Figure 1B: TNF-α signaling in Macrophage, airway epithelium and fibroblast.** TNF-α signaling contributes to asthma pathogenesis through inducing the epithelial secretion of proinflammatory cytokines and recruitment of inflammatory cells leading to airway inflammation (Brightling et al., 2008). TNF-α signaling promote fibroblast differentiation in to myofibroblast leading to airway remodeling (Brightling et al., 2008).


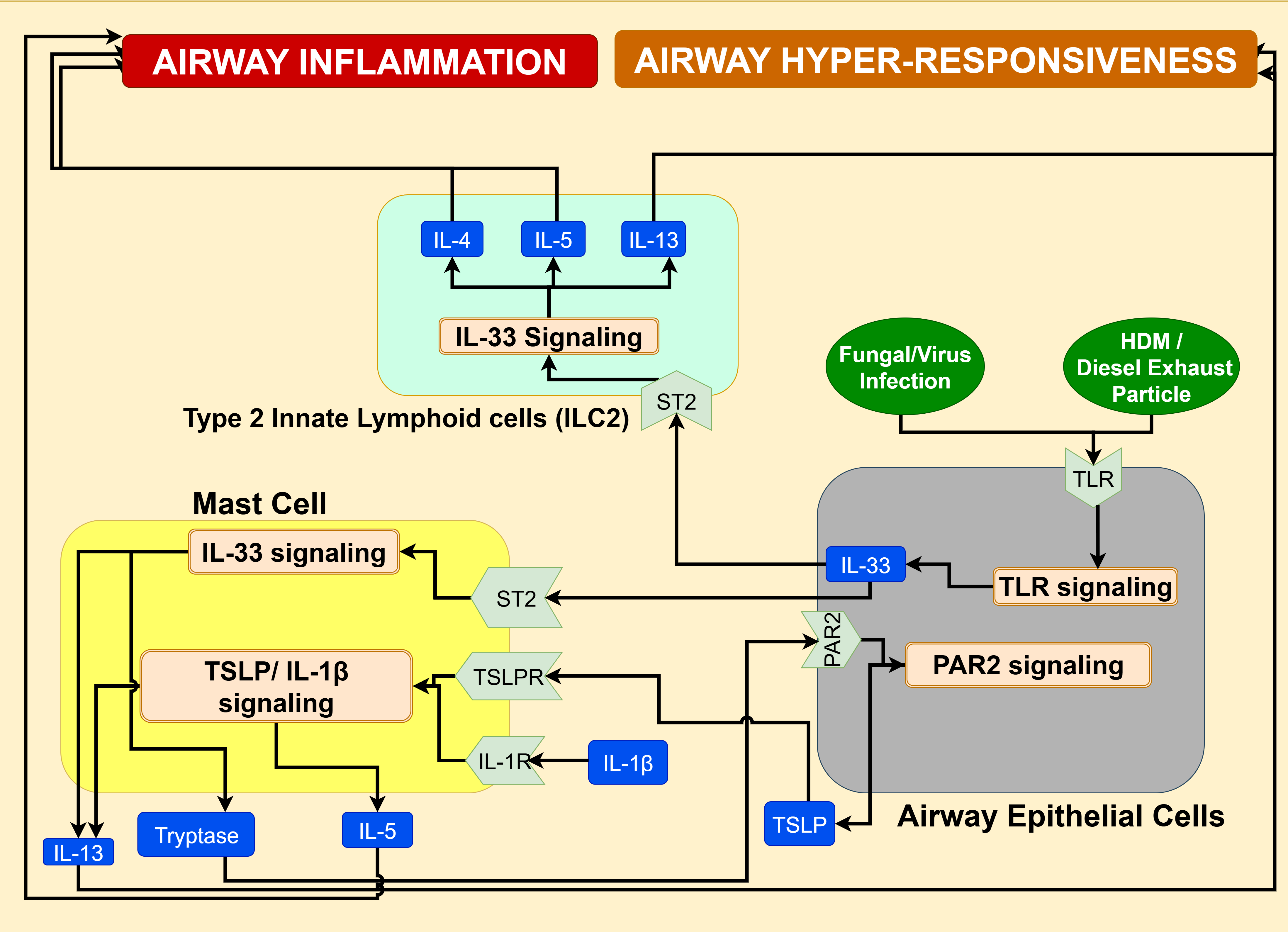


**Supplementary Figure 2A: IL-33 signaling in ILC2 and mast cells.** IL-33 signaling contributes to asthma pathogenesis by promoting type 2 airway inflammation (Yoshida et al., 2021).


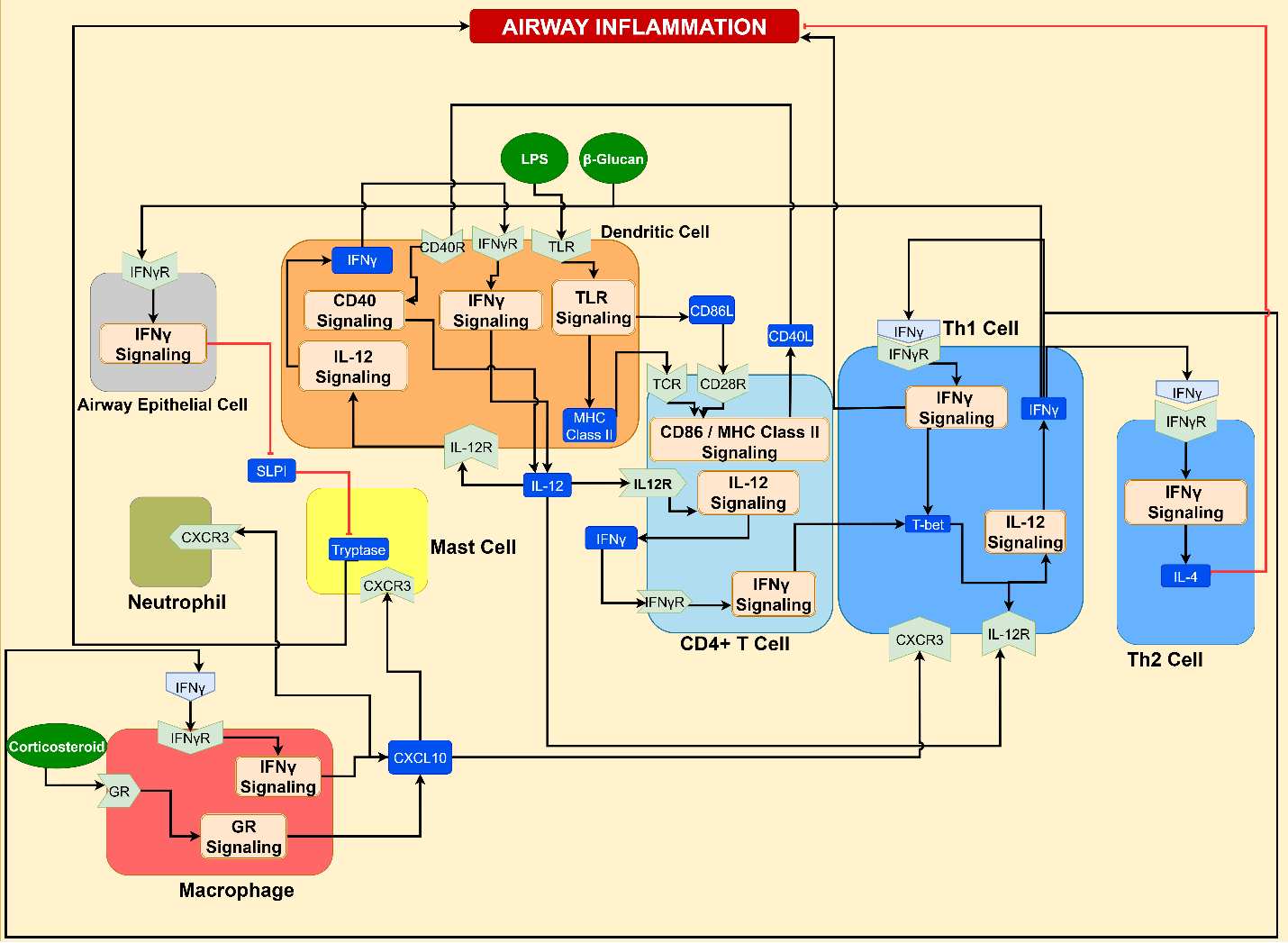


**Supplementary Figure 2B:** IL-12 and IFN-γ signaling in the airway microenvironment promotes the polarization of primed CD4+ T cells towards Th1 differentiation, recruitment of inflammatory cells, and mast cell activation, contributing to persistent airway inflammation.


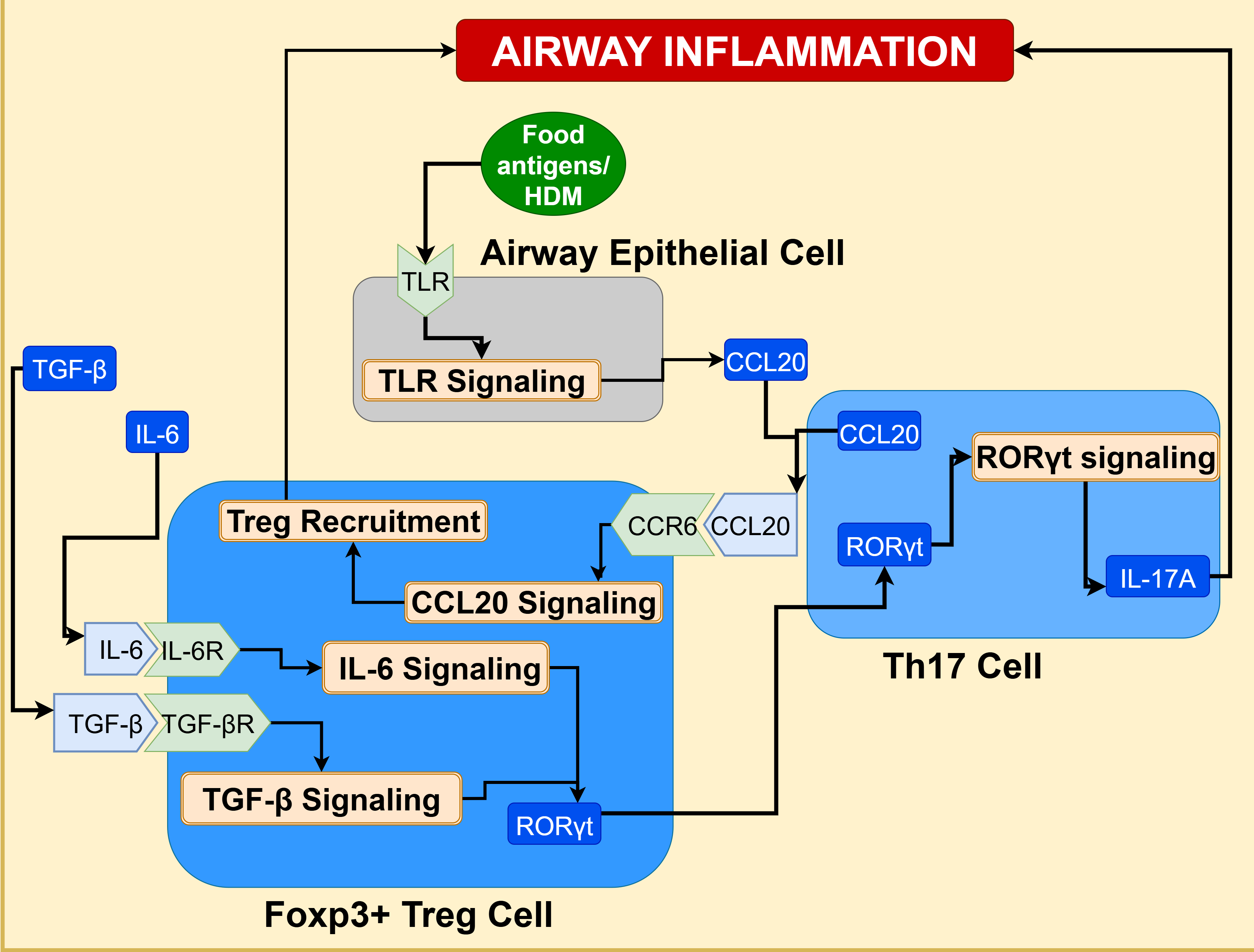


**Supplementary Figure 2C: TGF-β and IL-6 signaling in Treg cells.** TGF-β and IL-6 signaling contribute to asthma pathogenesis by inducing the differentiation of Treg to Th17 cells, contributing to Th17/Treg imbalance (Awasthi et al., 2008; Newcomb & Peebles, 2013; Vroman et al., 2015).


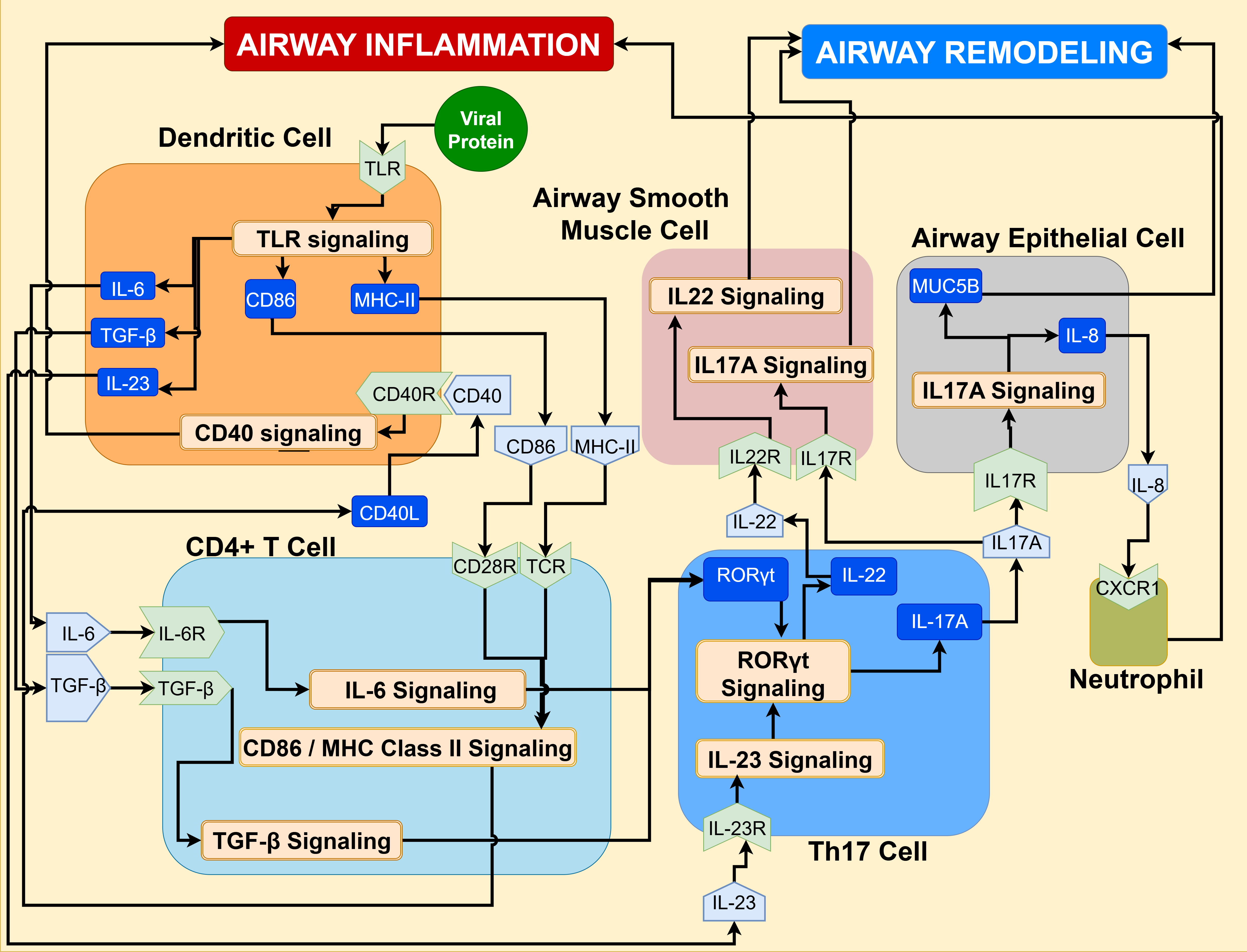


**Supplementary Figure 2D.** IL-6-TGF-β/IL-17 signaling in the airway microenvironment promotes the polarization of primed CD4+ T cells towards Th17 differentiation and promotes IL-17 signaling, leading to the recruitment of inflammatory cells, mucus hypersecretion, and ASMC proliferation, contributing to airway inflammation and remodeling.


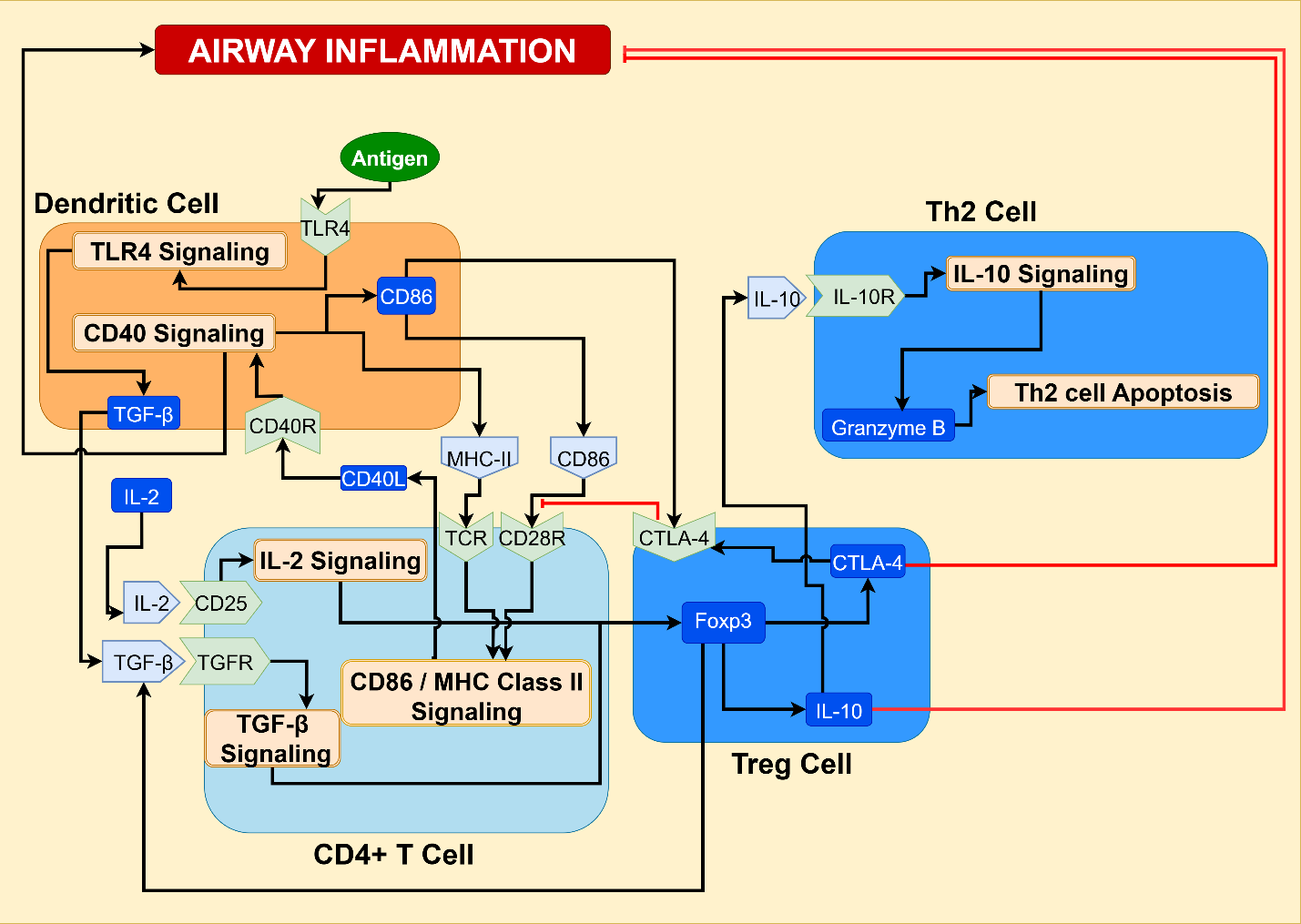


**Supplementary Figure 2E.** IL-2 and TGF-β signaling in the airway microenvironment stimulates the polarization of primed CD4+ T cells towards Treg differentiation and promotes IL-10 signaling, leading to suppression of costimulatory signaling by dendritic cells and inducing Th2 cell death, contributing to the attenuation of the airway


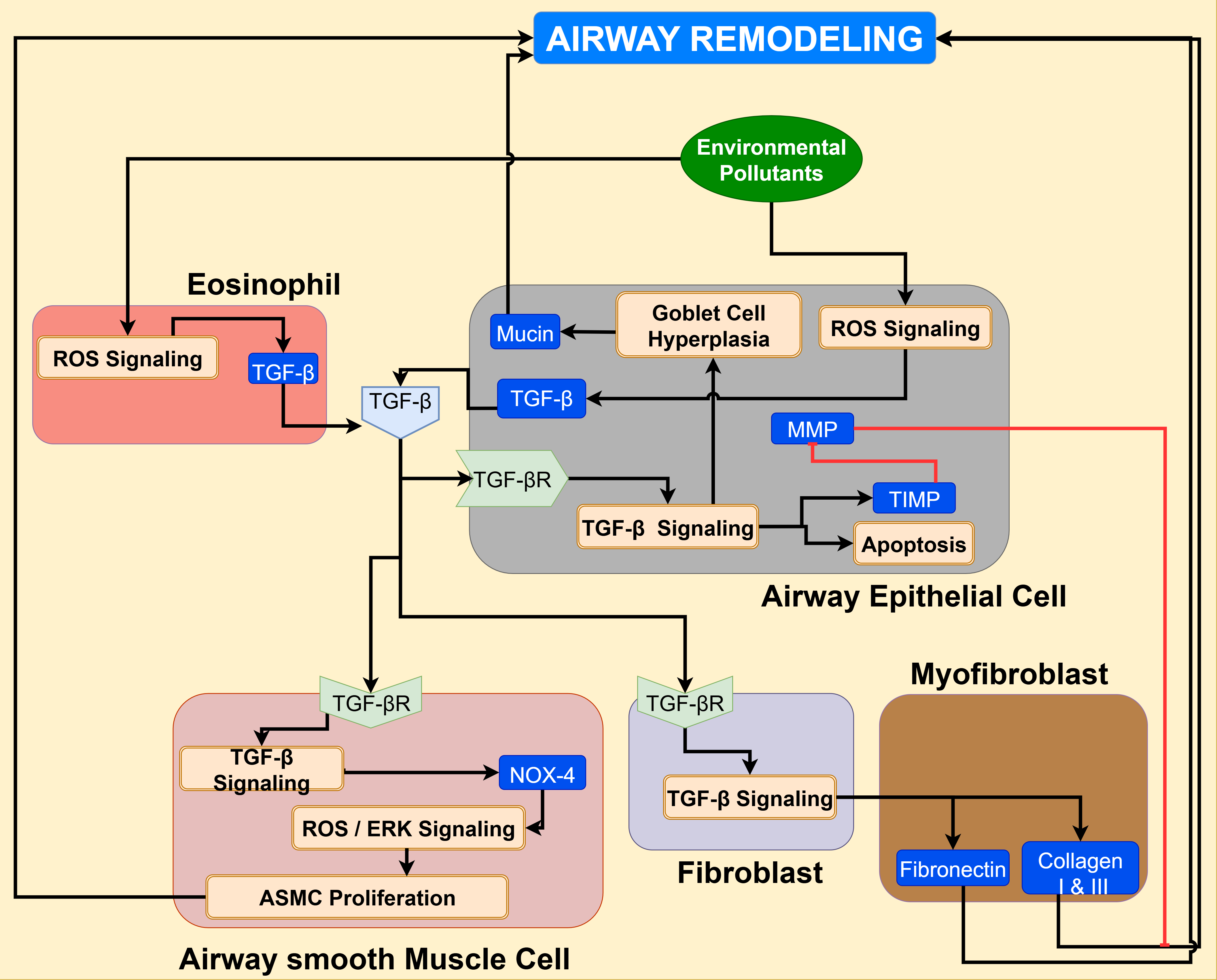


**Supplementary Figure 3A.** TGF-β1 signaling in airway microenvironment promotes smooth muscle cell proliferation, goblet cell hyperplasia and mucus hypersecretion, myofibroblast differentiation and production of extracellular matrix, contributing to remodeling of asthmatic airways. .


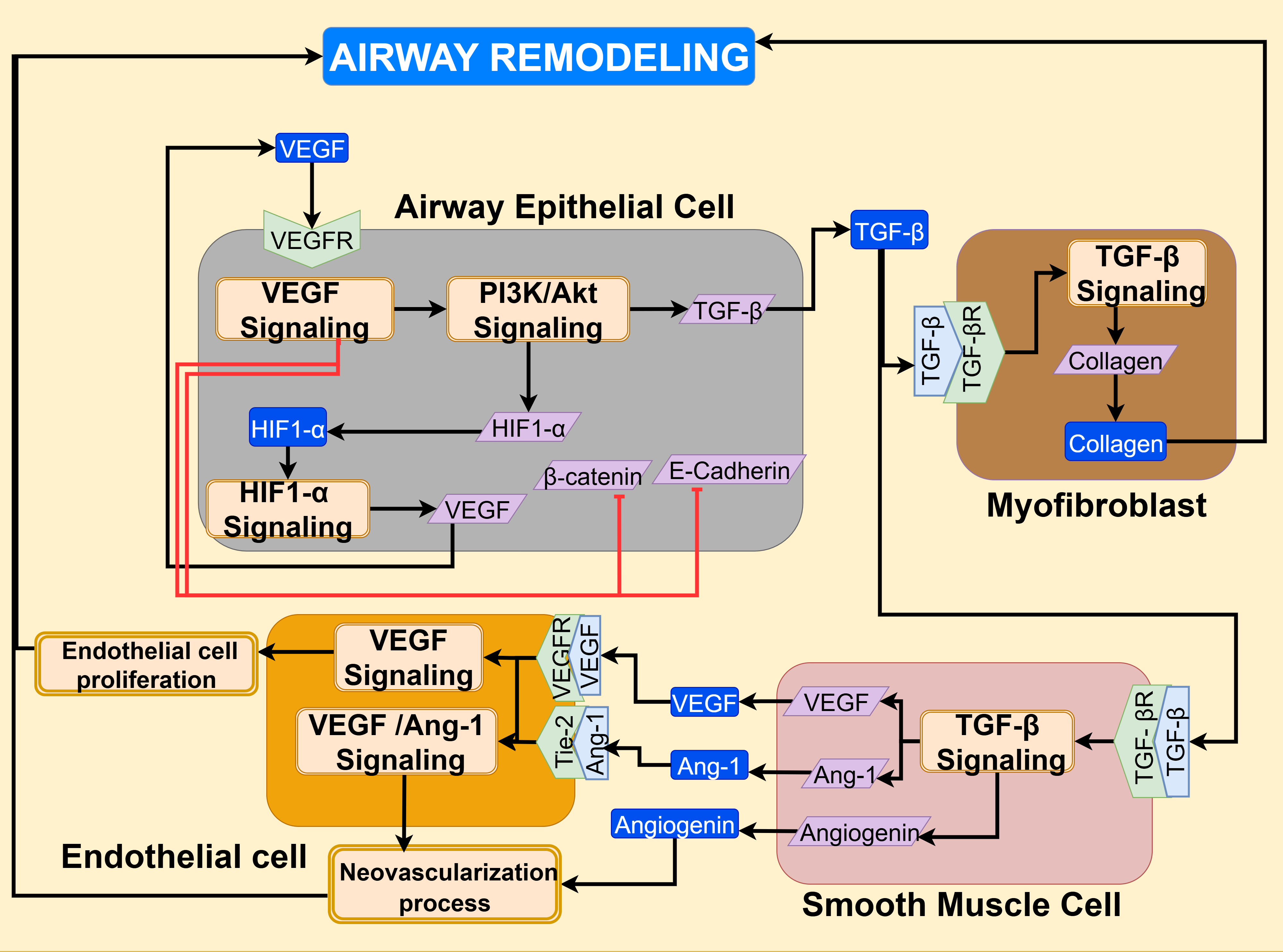


**Supplementary Figure 3B.** VEGF signaling in the airway microenvironment induces sub-epithelial fibrosis via upregulation of TGF-β in lung epithelial cells, causing accumulation of collagen I, III, and V, leading to thickening of the basement membrane and subsequent airway remodeling.


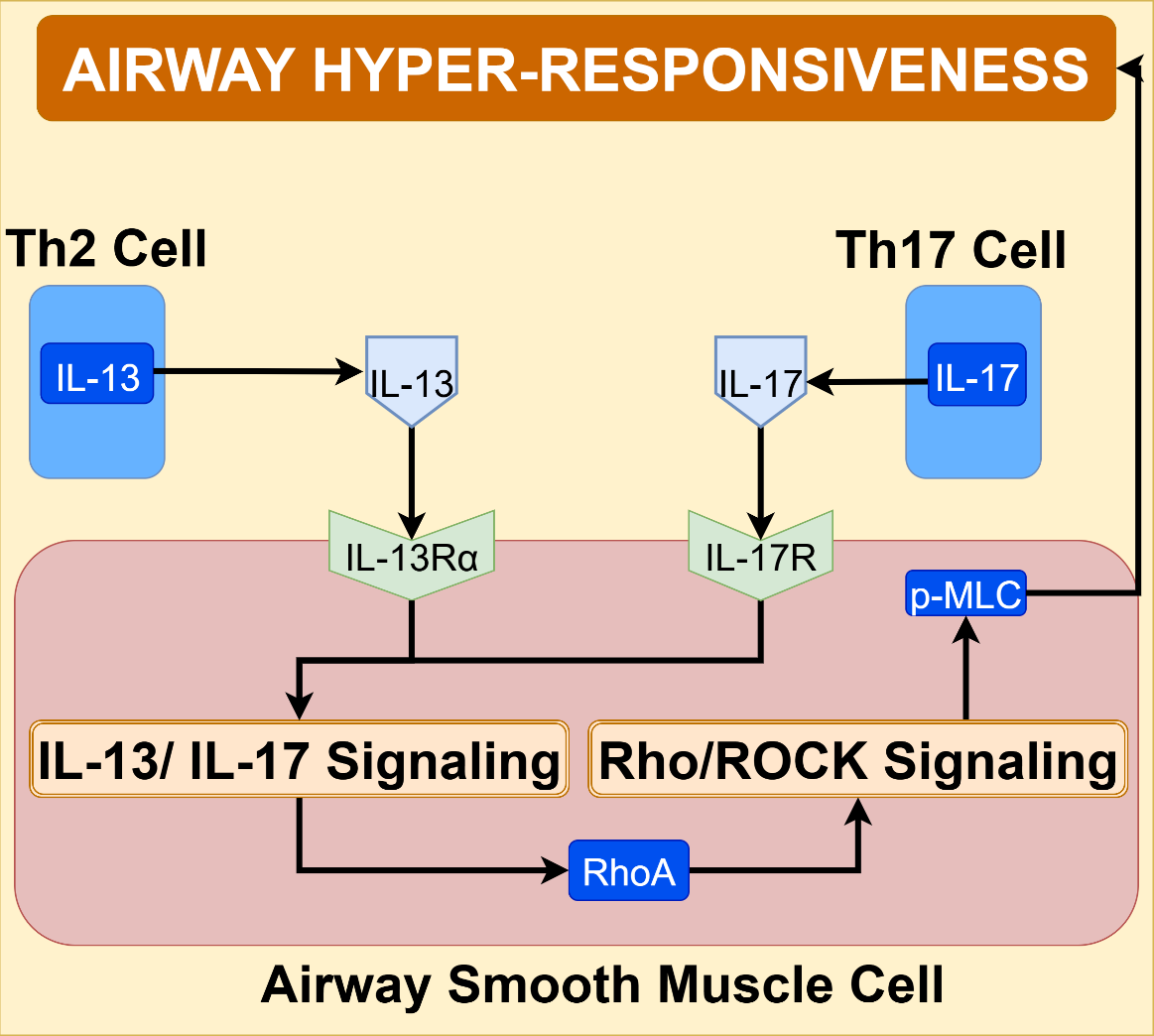


**Supplementary Figure 4:** IL-13/IL-17 signaling in the airway microenvironment with low levels of intracellular Ca2+ triggers bronchoconstriction and airway hyperresponsiveness.


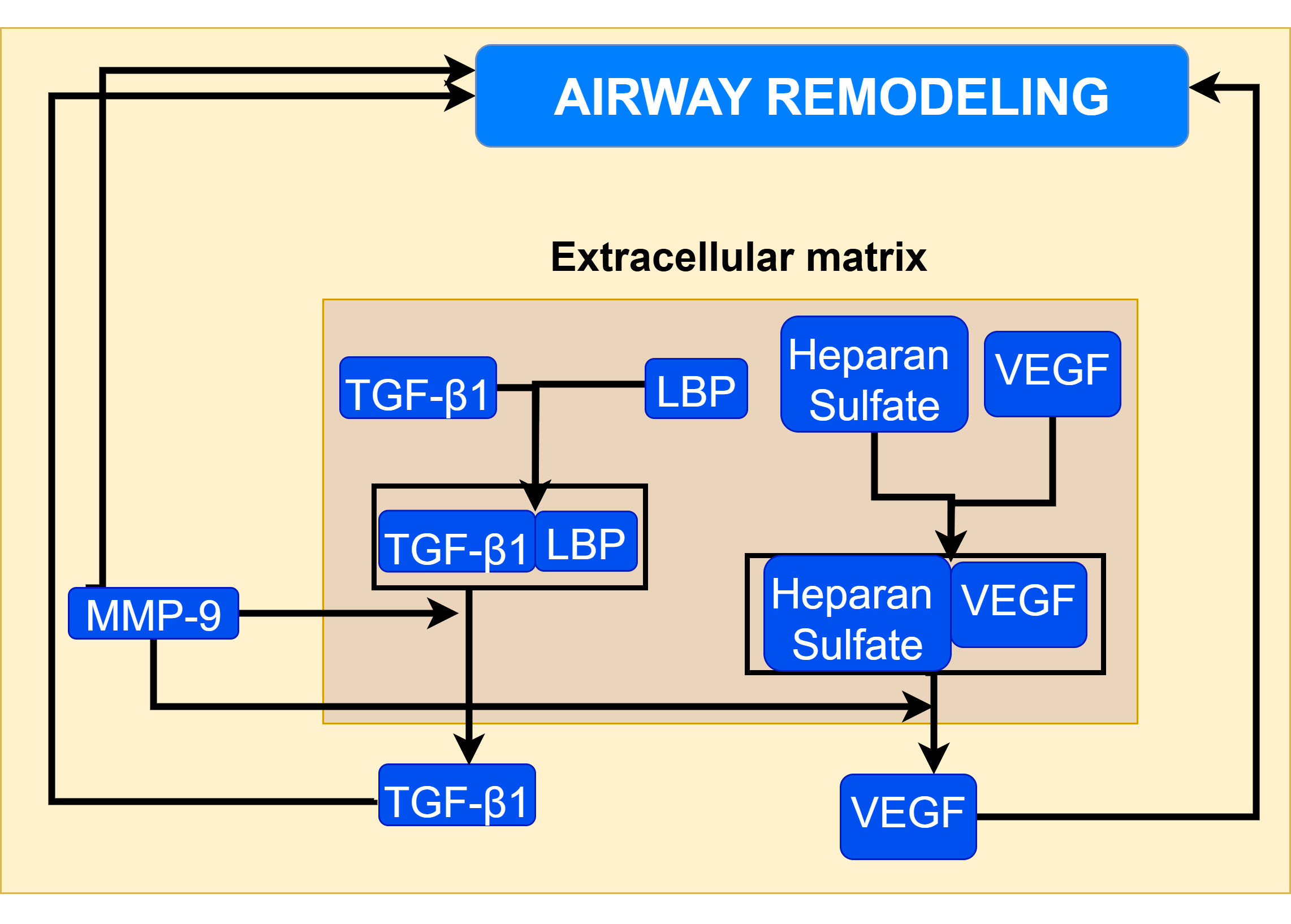


**Supplementary Figure 5:** **MMP-9 signaling in ECM**: airway microenvironment mediates proteolytic activation of the latent form of growth factors including TGFβ, VEGF and release from extracellular matrix, promoting the airway remodeling (Mott & Werb, 2004; Okada et al., 1997).


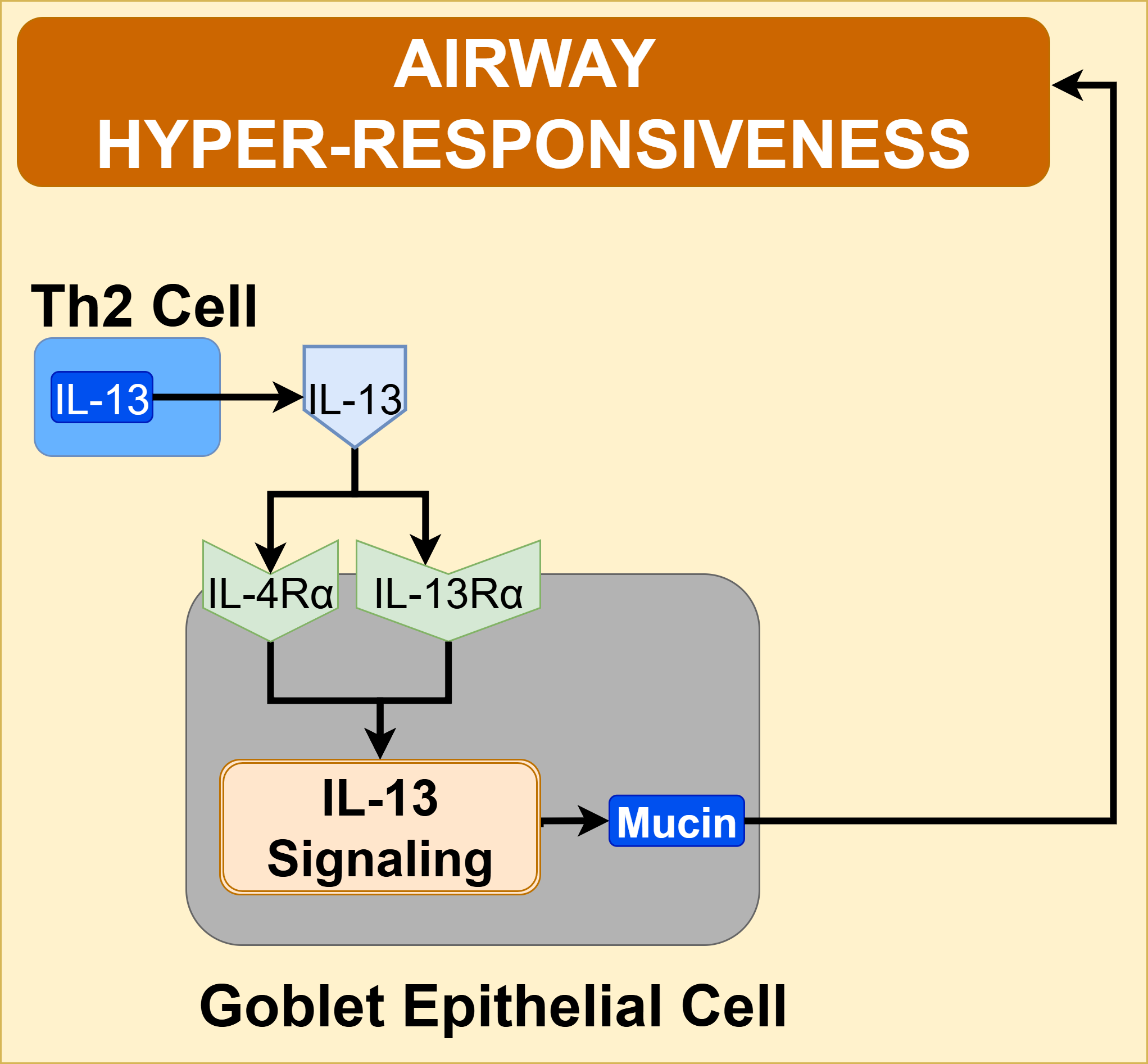


**Supplementary Figure 6: IL-13 signaling in Epithelial cell.** IL-13 signaling in epithelial cells contributes to asthma pathogenesis by promoting mucin secretion, contributing to airway hyperresponsiveness (Guan et al., 2018).


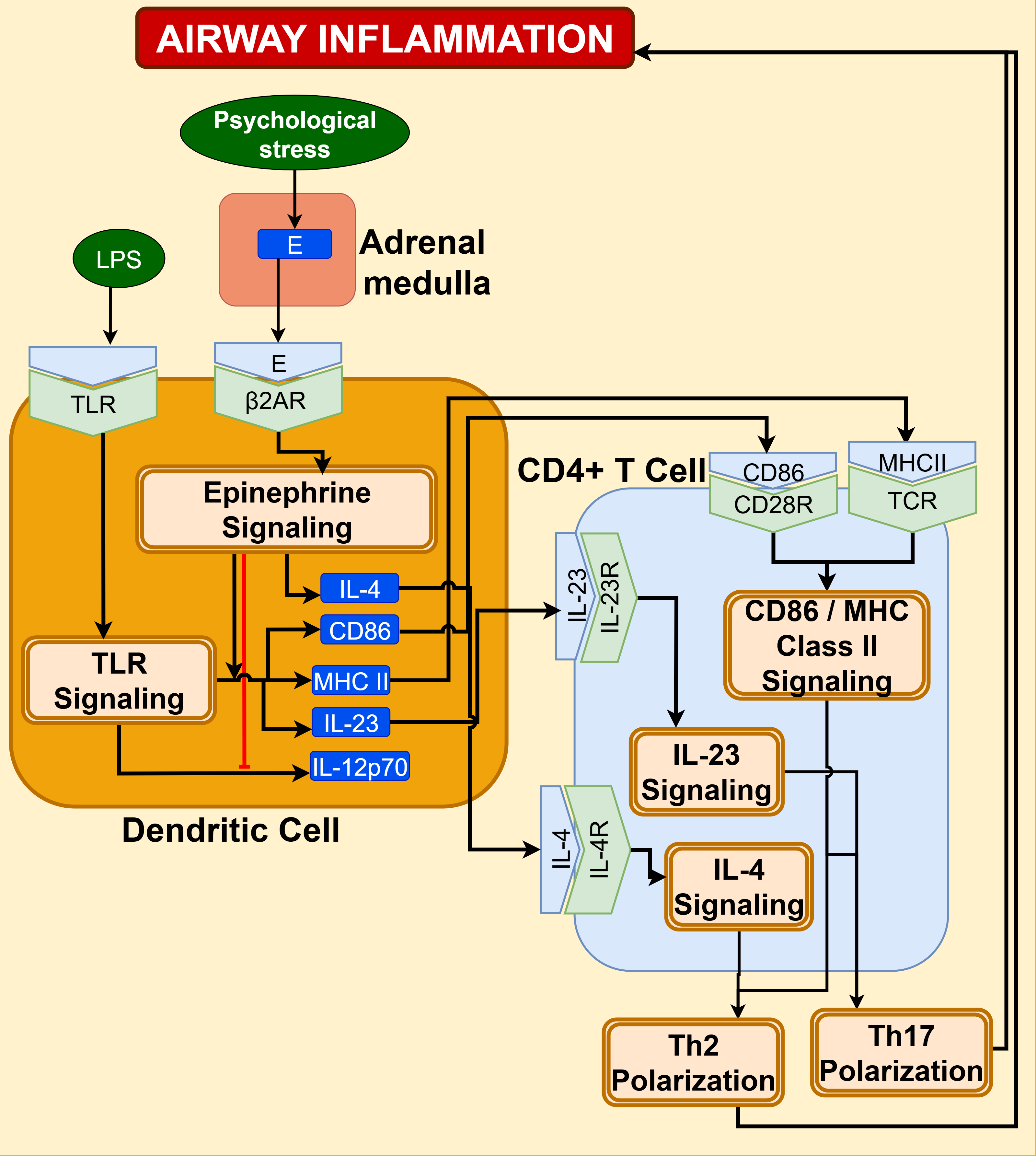


**Supplementary Figure 7:** Epinephrine signaling in dendritic cell contribute to asthma pathogenesis by upregulating the expression of IL-23 and IL-4 cytokines, promoting the Th2 and Th17 polarization, leading to airway inflammation (B.-J. Kim & Jones, 2010).

**Identification:**

**Articles identified through PubMed, Medline, and Google Scholar search**

***n* = 2,817**

(n = 20,231)

**Screening:**

**Duplicates removed**

***n* = 210**

***n* = 195**

**Eligibility:**

**Articles excluded (deemed not relevant)**

***n* = 1,843**

**Full-text articles assessed for eligibility**

***n* = 2,607**

**Included:**

**Studies included in the analysis
*n* = 764**

**Supplementary Figure 8:** PRISMA flow diagram. In the process above, 2,817 articles were identified; 210 duplicates were removed; 2,607 articles were eligible for review, from which 1,843 were removed as they were deemed not relevant; and 764 articles pertinent to the study objective were included in the analysis.


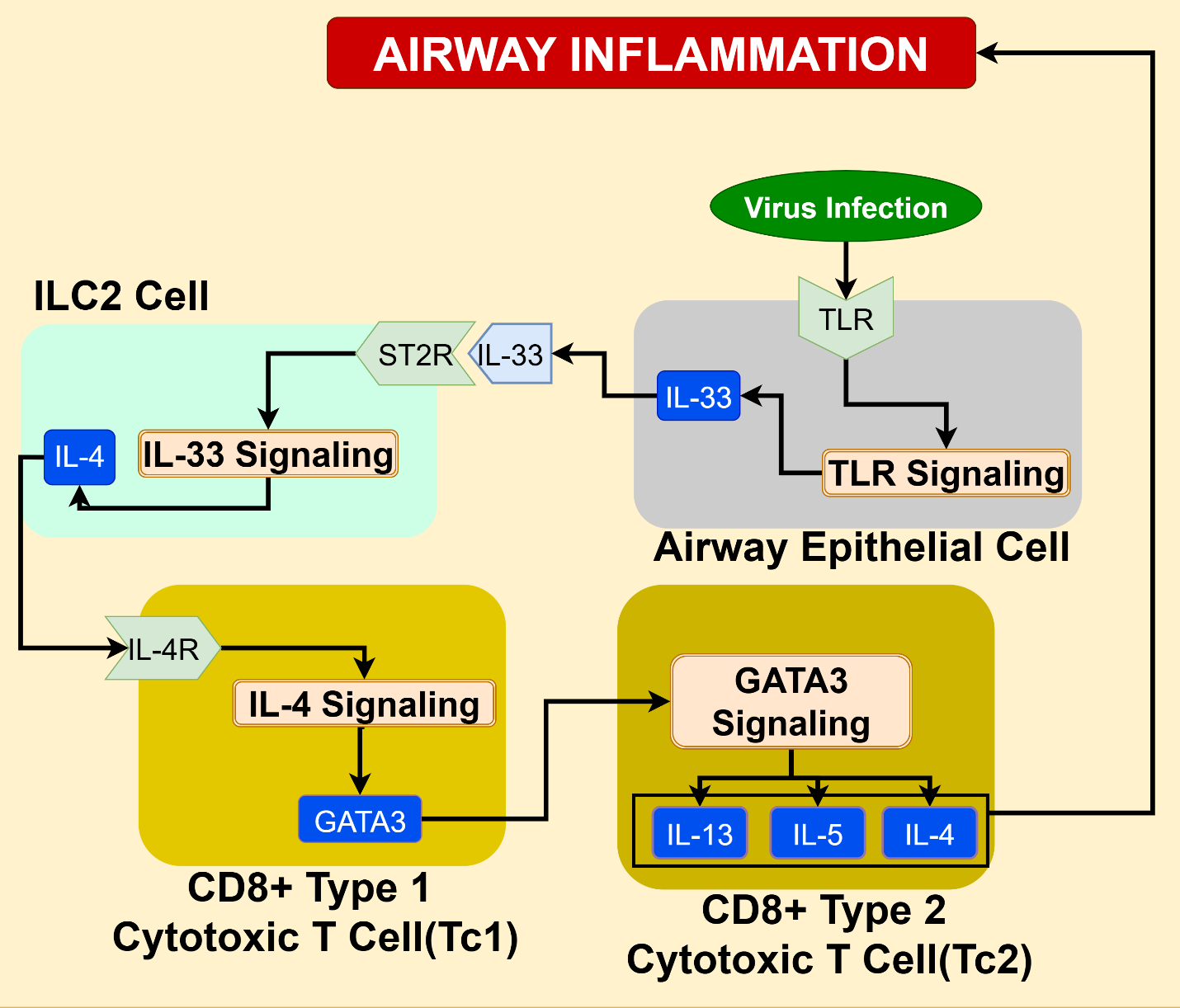


**Supplementary Figure 9:** IL-33/Tc2 cytokine signaling in the airway microenvironment promotes virus-triggered airway type 2 inflammation, contributing to exacerbation of asthma.


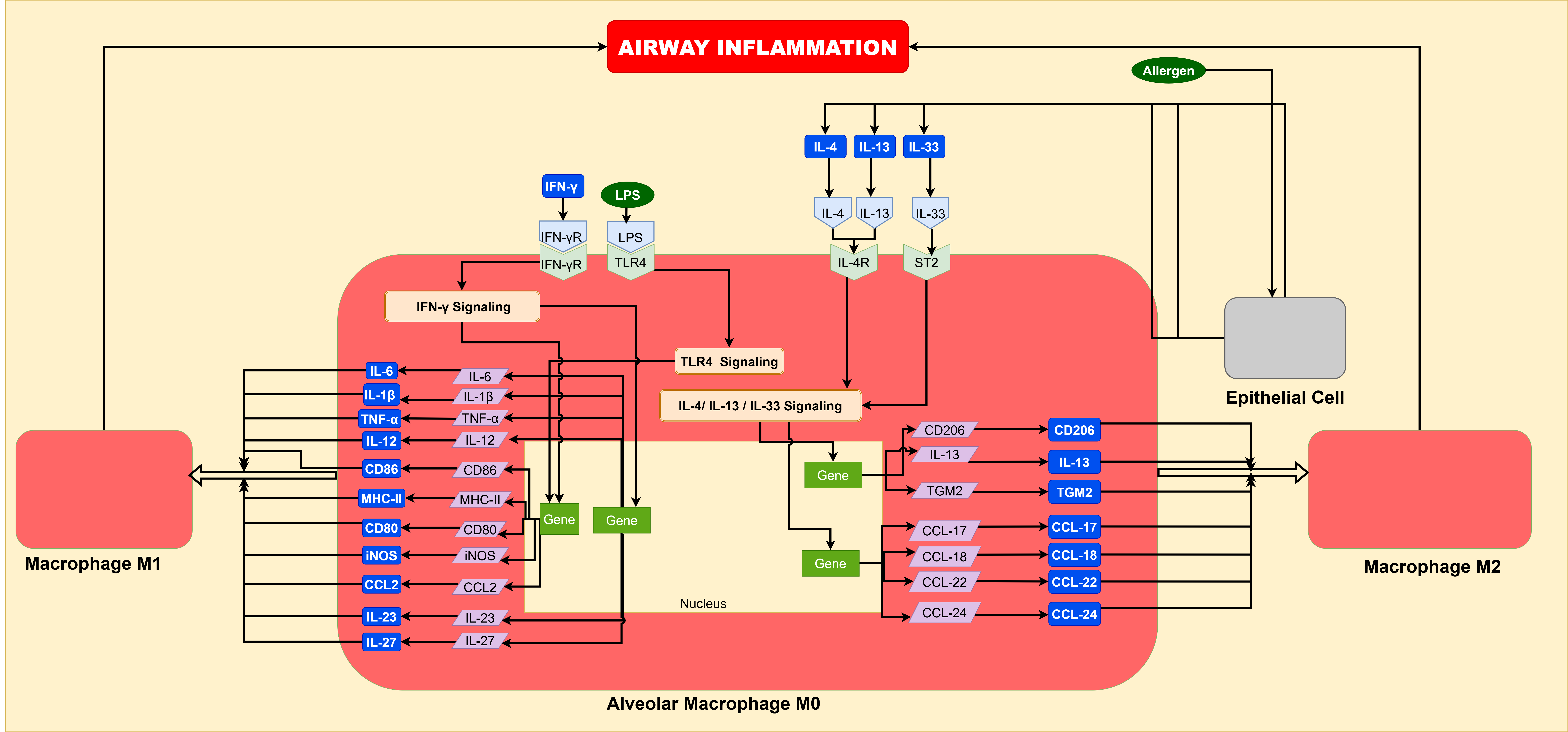


**Supplementary Figure 10:** Macrophage polarization in asthma. Proinflammatory cytokines IL-4, IL-13, IL-33 from epithelial cells induce M2a polarization; whereas LPS and IFNγ induce M1 polarization.

**3. Supplementary Tables**

**Table S1:** MeSH key words used to identify relevant literature

| **MeSH Keywords** |
| --- |
| Asthma and IL-33 AND CD8+ cells |
| Asthma AND Cellular AND Molecular mechanisms AND Allergic inflammation |
| Asthma AND Oxidative stress AND Lung epithelium |
| Asthma AND NADPH oxidase signaling |
| Dual oxidase AND Therapeutic target AND Allergen |
| Asthma pathogenesis AND PM2.5 AND Oxidative stress |
| Human Asthma AND Toll-like receptors AND Experimental models |
| Asthma endotypes AND Phenotypes AND Treatable traits |
| Targeting TNF-α AND Therapeutic approach AND Asthma |
| Dendritic cell activation AND Th2/Th17 AND CD4+ T cell differentiation AND Asthma |
| Asthma AND Dendritic cell AND Lung immunopathology |
| Regulatory T Cell AND Asthma And Interleukin 10 And TGF-β |
| Th17 inflammatory response And Severe neutrophilic asthma |
| Th2 inflammation And GATA-3 and Allergic asthma |
| Macrophage AND T cell crosstalk AND Asthma |
| Th cell subset polarization AND Asthma pathogenesis |
| Severe asthma AND Interferon gamma AND Airway hyperresponsiveness |
| Type 2 And Non-type 2 AND Inflammation AND Asthma |
| Cytokine signaling AND CD4+ T cell differentiation AND Transcriptional regulation AND Epigenetic modification |
| Asthma AND Airway epithelial and Smooth muscle cell pathology |
| Nitric oxide synthase AND Soluble guanylyl cyclase AND Bronchial epithelium |
| Nitric oxide AND Redox regulation AND Airway epithelial cells |
| Airway remodeling and Lung fibrosis and Asthma |
| VEGF and Airway remodeling |
| TGF-β AND Airway remodeling and Hyperresponsiveness and Asthma |
| Gene polymorphisms and Environmental factors and Lung function and Asthma |
| Environmental risk factors AND Epigenetics AND Genetics AND Asthma pathogenesis |
| TGF-β AND Eosinophil and Severe asthma |
| Psychological stress AND asthma AND Inflammation |
| Emotional stress AND neuroimmune crosstalk AND asthma |
| Neuroimmune pathophysiology AND bronchoconstriction |
| Psychological stress and bronchoconstriction |


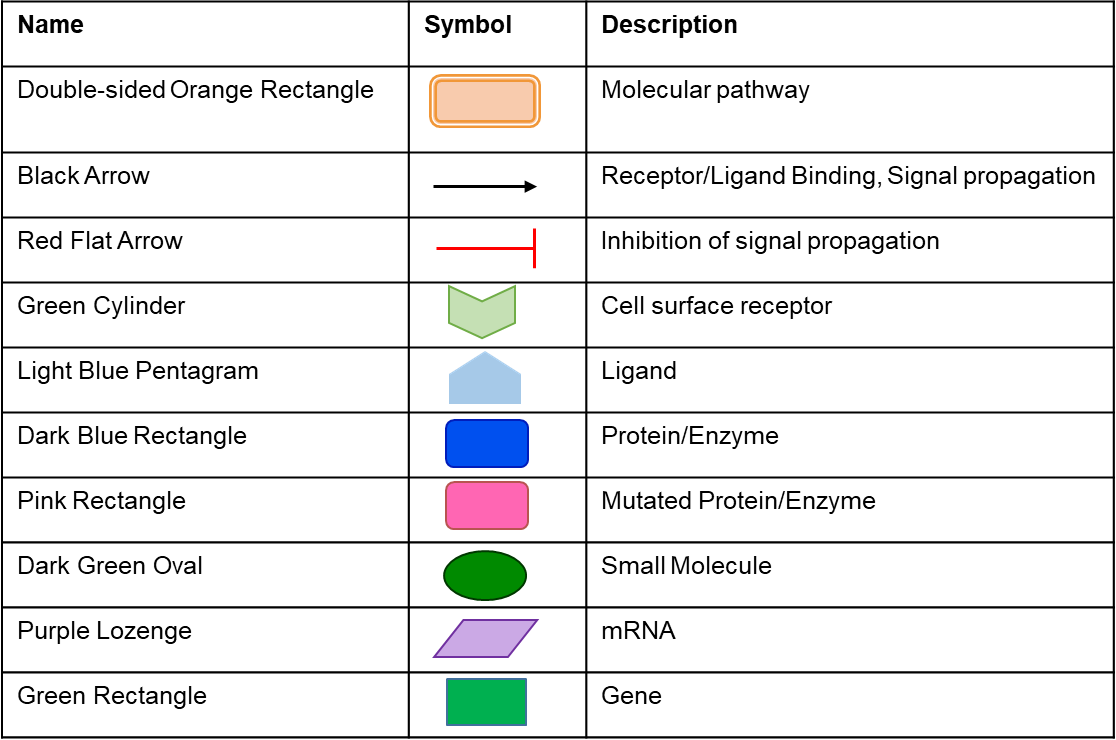
**Table S2.** Legend of symbols used in interactome figures.

**4. Glossary – Main Manuscript and Supplemental Figures**

**Figure 1. The Asthma Lung Microenvironment (ALM).**

**Abbreviations**: ROS, Reactive oxygen species; Th2, Type 2 T helper cell; Th17, T helper 17 cell; ILC2, Type 2 innate lymphoid cell.

**Figure 2. Neuro-immune interactions triggered by psychological stress in ALM.**

**Abbreviations:** BBB, Blood-brain barrier; ACh, Acetylcholine; TRPV1, Transient receptor potential vanilloid 1; mAChR, Muscarinic acetylcholine receptor; ASMC, Airway smooth muscle cell; nAChR, Nicotinic acetylcholine receptor; NK1R, Neurokinin-1 receptor; NP, Neuropeptide; SP, Substance P; NANC, Non-adrenergic, non-cholinergic; CRH, Corticotropin-releasing hormone; ACTH, Adrenocorticotropic hormone; NE, Norepinephrine; LC, Locus coeruleus; SAM, Sympathetic adrenal medullary system; α1AR, α1-Adrenergic receptor; β2AR, Beta-2 adrenergic receptor; HPA, Hypothalamic-pituitary-adrenal axis; GC, Glucocorticoid; GRα, Glucocorticoid receptor α.

**Figure 3. Molecular systems architecture of airway microenvironment signaling in asthma.**

**Abbreviations:** TLR, Toll-like receptor; NOX, NADPH oxidase; IL-33, Interleukin-33; TNFα, Tumor necrosis factor alpha; IL-4, Interleukin-4; IL-12, Interleukin-12; IFNγ, Interferon gamma; IL-6, Interleukin-6; TGF-β, Transforming growth factor beta; IL-17, Interleukin-17; IL-2, Interleukin-2; IL-10, Interleukin-10; IL-13, Interleukin-13; NO, Nitric oxide; EGFR, Epidermal growth factor receptor; MMP-9, Matrix metalloproteinase-9; VEGF, Vascular endothelial growth factor; HDM, House dust mite; ORMDL3, ORMDL sphingolipid biosynthesis regulator 3; GSDMB, Gasdermin B; IL1RL1, Interleukin-1 receptor-like 1; TSLP, Thymic stromal lymphopoietin; HLA, Human leukocyte antigen; Foxp3, Forkhead box P3; IL-9, Interleukin-9; CCR3, CC chemokine receptor 3; CRH, Corticotropin-releasing hormone; GC, Glucocorticoid; TRPV1, Transient receptor potential vanilloid 1; NE, Norepinephrine; ACh, Acetylcholine; SP, Substance P; IL-1β, Interleukin-1 beta.

**Figure 4. Interactome of the molecular systems architecture of the airway microenvironment.**

**Abbreviations:** YKL-40, Chitinase-3-like protein 1; MMP-9, Matrix metalloproteinase-9; ROS, Reactive oxygen species; MUC5AC, Mucin 5AC; DUOX, Dual oxidase; NOX, NADPH oxidase; RNS, Reactive nitrogen species; TGF-β, Transforming growth factor beta; EGFR, Epidermal growth factor receptor; iNOS, Inducible nitric oxide synthase; CXCL1, C-X-C motif chemokine ligand 1; IL-1β, Interleukin-1 beta; IL-8, Interleukin-8; G-CSF, Granulocyte colony-stimulating factor; HIF-1α, Hypoxia-inducible factor-1 subunit alpha; ICAM-1, Intercellular adhesion molecule-1; TLR, Toll-like receptor; PAR, Protease-activated receptor; GM-CSF, Granulocyte-macrophage colony-stimulating factor; VEGF, Vascular endothelial growth factor; IL-13, Interleukin-13; CCL20, C-C motif chemokine ligand 20; IL-33, Interleukin-33; TNFα, Tumor necrosis factor alpha; IFNγ, Interferon gamma; IL-17A, Interleukin-17A; IL-6, Interleukin-6; IL-25, Interleukin-25; HDM, House dust mite; ASMC, Airway smooth muscle cell; IL-22, Interleukin-22; ROCK, Rho-associated coiled-coil containing protein kinase; sGC, Soluble guanylate cyclase; IgE, Immunoglobulin E; IL-4, Interleukin-4; IL-5, Interleukin-5; IL-12, Interleukin-12; CD40R, Cluster of differentiation 40 receptor; TSLP, Thymic stromal lymphopoietin; IL-23R, Interleukin-23 receptor; MHC, Major histocompatibility complex; TCR, T cell receptor; RORγt, Retinoid orphan receptor gamma t; CCR6, C-C chemokine receptor type 6; FoxP3, Forkhead box P3; CTLA, Cytotoxic T-lymphocyte antigen 4; IL-10, Interleukin-10; IL-9, Interleukin-9; BBB, Blood-brain barrier; ACh, Acetylcholine; TRPV1, Transient receptor potential vanilloid 1; mAChR, Muscarinic acetylcholine receptor; ASMC, Airway smooth muscle cell; nAChR, Nicotinic acetylcholine receptor; NK1R, Neurokinin-1 receptor; NP, Neuropeptide; SP, Substance P; NANC, Non-adrenergic, non-cholinergic; CRH, Corticotropin-releasing hormone; ACTH, Adrenocorticotropic hormone; NE, Norepinephrine; LC, Locus coeruleus; SAM, Sympathetic adrenal medullary system; α1AR, α1-Adrenergic receptor; β2AR, Beta-2 adrenergic receptor; HPA, Hypothalamic-pituitary-adrenal axis; GC, Glucocorticoid; GRα, Glucocorticoid receptor α.

**Figure 5. NOX/ROS signaling in the airway microenvironment.**

**Abbreviations:** TLR, Toll-like receptor; PAR2, Protease-activated receptor 2; EGFR, Epidermal growth factor receptor; DUOX, Dual oxidase; NOX, NADPH oxidase; Nrf-2, Nuclear factor erythroid 2-related factor 2; RNS, Reactive nitrogen species; ROS, Reactive oxygen species; IL-13R, Interleukin-13 receptor; CCL20, C-C motif chemokine ligand 20; CXCR6, C-X-C motif chemokine receptor 6; IL-8, Interleukin-8; IL-33, Interleukin-33; HDM, House dust mite; ST2, Interleukin-1 receptor-like 1.

**Figure 6. IL-4 signaling in the airway microenvironment.**

**Abbreviations**: TLR, Toll-like receptor; IL, Interleukin; CD, Cluster of differentiation; TSLP, Thymic stromal lymphopoietin; ST2, Interleukin-1 receptor-like 1; CCR3, C-C chemokine receptor type 3; PGE2, Prostaglandin E2; IgE, Immunoglobulin E; MHC, Major histocompatibility complex; FcεRI, Fc epsilon RI; ILC2, Type 2 innate lymphoid cells.

**Figure 7. EGFR signaling in the airway microenvironment.**

**Abbreviations:** YKL-40, Chitinase-3-like protein 1; EGFR, Epidermal growth factor receptor; TNFα, Tumor necrosis factor alpha; HB-EGF, Heparin-binding EGF-like growth factor.

**Figure 8. NO signaling in the airway microenvironment.**

**Abbreviations:** sGC, Soluble guanylate cyclase; IL, Interleukin; TNFα, Tumor necrosis factor alpha; IFNγ, Interferon gamma; NOX, NADPH oxidase; cGMP, Cyclic GMP; iNOS, Inducible nitric oxide synthase; NO, Nitric oxide; GTP, Guanosine-5'-triphosphate.

**Figure 9. CCR3 signaling in the blood-brain barrier microenvironment.**

**Abbreviations**: CCR3, C-C chemokine receptor type 3; BBB, Blood-brain barrier; NO, Nitric oxide; PG, Prostaglandin; NOX, NADPH oxidase; ROS, Reactive oxygen species.

**Figure 10. TRPV1 signaling in afferent vagal neurons.**

**Abbreviations:** TRPV1, Transient receptor potential cation channel subfamily V member 1; NP, Neuropeptide; ACh, Acetylcholine; NTS, Nucleus tractus solitarius; mAChR, Muscarinic acetylcholine receptor; ASMC, Airway smooth muscle cell.

**Figure 11. Parasympathetic ACh/PLC/IP3-PKC signaling in ASMC.**

**Abbreviations**: ACh, Acetylcholine; ASMC, Airway smooth muscle cell; EGF, Epidermal growth factor; EGFR, Epidermal growth factor receptor; TGF-β, Transforming growth factor beta; mAChR, Muscarinic acetylcholine receptor; Ca2+, Calcium ions.

**Figure 12. Substance P signaling in airway epithelium and airway smooth muscle cells.**

**Abbreviations**: SP, Substance P; NK1R, Neurokinin-1 receptor; ASMC, Airway smooth muscle cell.

**Figure 13. CRH signaling axis under the influence of psychological stress.**

**Abbreviations**: CRH, Corticotropin-releasing hormone; ACTH, Adrenocorticotropic hormone; NE, Norepinephrine; GC, Glucocorticoid; α1AR, α1-Adrenergic receptor; cAMP, Cyclic adenosine monophosphate.

**Figure 14. Glucocorticoid signaling in bronchial epithelium.**

**Abbreviations:** GC, Glucocorticoid; GRα, Glucocorticoid receptor α; IL, Interleukin; TNFα, Tumor necrosis factor alpha; LPS, Lipopolysaccharide.

**Figure 15. NE/IL-1β signaling augments Th2 and Th17 airway inflammation.**

**Abbreviations**: CRH, Corticotropin-releasing hormone; NE, Norepinephrine; ACh, Acetylcholine; IL-1β, Interleukin-1 beta; β2AR, Beta-2 adrenergic receptor.

**Supplementary Figure 1A. TLR signaling in airway epithelium.**

Abbreviations: HDM, House dust mite; LPS, Lipopolysaccharides; MyD88, Myeloid differentiation primary response 88; TRIF, TIR-domain-containing adapter-inducing interferon-β; CXCL1, C-X-C motif chemokine ligand 1; CXCR6, C-X-C motif chemokine receptor 6; CCL20, C-C motif chemokine ligand 20; VEGF, Vascular endothelial growth factor; TNFα, Tumor necrosis factor alpha; MHC, Major histocompatibility complex; IL, Interleukin; TCR, T cell receptor; CD, Cluster of differentiation; GM-CSF, Granulocyte-macrophage colony-stimulating factor.

**Supplementary Figure 1B. TNF-α signaling in macrophage, airway epithelium and fibroblast.**

Abbreviations: TNFα, Tumor necrosis factor alpha; ROS, Reactive oxygen species; MAPK, Mitogen-activated protein kinase; NF-κB, Nuclear factor kappa B; MUC5AC, Mucin 5AC; TGF-β, Transforming growth factor beta.

**Supplementary Figure 2A. IL-33 signaling in ILC2 and mast cells.**

Abbreviations: IL-33, Interleukin-33; ILC2, Type 2 innate lymphoid cells; ST2, Interleukin-1 receptor-like 1; TSLP, Thymic stromal lymphopoietin; PAR2, Protease-activated receptor 2.

**Supplementary Figure 2B. IL-12 and IFN-γ signaling in the airway microenvironment.**

Abbreviations: IFNγ, Interferon gamma; CXCR3, C-X-C motif chemokine receptor 3; SLPI, Secretory leukocyte protease inhibitor; GR, Glucocorticoid receptor; CXCL10, C-X-C motif chemokine ligand 10; TLR, Toll-like receptor; CD, Cluster of differentiation; IL, Interleukin; LPS, Lipopolysaccharides.

**Supplementary Figure 2C. TGF-β and IL-6 signaling in Treg cells.**

Abbreviations: TGF-β, Transforming growth factor beta; IL, Interleukin; Treg, Regulatory T cell; RORγt, Retinoid orphan receptor gamma t; STAT3, Signal transducer and activator of transcription 3.

**Supplementary Figure 2D. IL-6-TGF-β/IL-17 signaling in the airway microenvironment.**

Abbreviations: TLR, Toll-like receptor; CD, Cluster of differentiation; IL, Interleukin; TGF-β, Transforming growth factor beta; MHC, Major histocompatibility complex; MUC5B, Mucin 5B; RORγt, Retinoid orphan receptor gamma t; TCR, T cell receptor; CXCR1, C-X-C motif chemokine receptor 1; ASMC, Airway smooth muscle cell.

**Supplementary Figure 2E. IL-2 and TGF-β signaling in the airway microenvironment.**

Abbreviations: TLR, Toll-like receptor; CD, Cluster of differentiation; IL, Interleukin; TGF-β, Transforming growth factor beta; MHC, Major histocompatibility complex; FoxP3, Forkhead box P3; CTLA, Cytotoxic T-lymphocyte antigen 4.

**Supplementary Figure 3A. TGF-β1 signaling in the airway microenvironment.**

Abbreviations: ROS, Reactive oxygen species; TGF-β, Transforming growth factor beta; MMP, Matrix metalloproteinase; TIMP, Tissue inhibitor of metalloproteinases; NOX, NADPH oxidase; ASMC, Airway smooth muscle cell.

**Supplementary Figure 3B. VEGF signaling in the airway microenvironment.**

Abbreviations: VEGF, Vascular endothelial growth factor; TGF-β, Transforming growth factor beta; HIF-1α, Hypoxia-inducible factor-1 subunit alpha; PI3K, Phosphoinositide 3-kinase; Ang-1, Angiopoietin-1.

**Supplementary Figure 4. IL-13/IL-17 signaling in the airway microenvironment.**

Abbreviations: IL, Interleukin; ROCK, Rho-associated coiled-coil containing protein kinase; p-MLC, Phosphorylated myosin light chain; RhoA, Ras homolog family member A; MYPT-1, Myosin phosphatase target subunit 1.

**Supplementary Figure 5. MMP-9 signaling in ECM.**

Abbreviations: MMP-9, Matrix metalloproteinase-9; ECM, Extracellular matrix; TGF-β, Transforming growth factor beta; VEGF, Vascular endothelial growth factor; LAP, Latent-associated peptide; LTBP, Latent TGF-β binding protein.

**Supplementary Figure 6. IL-13 signaling in epithelial cell.**

Abbreviations: IL-13, Interleukin-13; MUC5AC, Mucin 5AC; ERK1/2, Extracellular signal-regulated kinase 1/2; p38, p38 mitogen-activated protein kinase.

**Supplementary Figure 7. Epinephrine signaling in dendritic cell.**

Abbreviations: β2AR, Beta-2 adrenergic receptor; IL, Interleukin; MHC, Major histocompatibility complex; CD, Cluster of differentiation; Th2, Type 2 T helper cell; Th17, T helper 17 cell; cAMP, Cyclic adenosine monophosphate.

**Supplementary Figure 9. IL-33/Tc2 cytokine signaling in the airway microenvironment.**

Abbreviations: TSLP, Thymic stromal lymphopoietin; ILC2, Type 2 innate lymphoid cells; IL-33, Interleukin-33; ST2, Interleukin-1 receptor-like 1.

**Supplementary Figure 10. Macrophage polarization in asthma.**

Abbreviations: IL, Interleukin; LPS, Lipopolysaccharides; IFNγ, Interferon gamma; M1, Classically activated macrophage; M2, Alternatively activated macrophage.

**4. REFERENCES:**

Abdelaziz, M. H., Abdelwahab, S. F., Wan, J., Cai, W., Huixuan, W., Jianjun, C., Kumar, K. D., Vasudevan, A., Sadek, A., Su, Z., Wang, S., & Xu, H. (2020). Alternatively activated macrophages; A double-edged sword in allergic asthma. *Journal of Translational Medicine*, *18*(1), 1–12. https://doi.org/10.1186/s12967-020-02251-w

Al-Alawi, M., Hassan, T., & Chotirmall, S. H. (2014). Transforming growth factor β and severe asthma: A perfect storm. In *Respiratory Medicine* (Vol. 108, Issue 10, pp. 1409–1423). W.B. Saunders Ltd. https://doi.org/10.1016/j.rmed.2014.08.008

Allakhverdi, Z., Comeau, M. R., Jessup, H. K., Yoon, B. R. P., Brewer, A., Chartier, S., Paquette, N., Ziegler, S. F., Sarfati, M., & Delespesse, G. (2007). Thymic stromal lymphopoietin is released by human epithelial cells in response to microbes, trauma, or inflammation and potently activates mast cells. *Journal of Experimental Medicine*, *204*(2), 253–258. https://doi.org/10.1084/jem.20062211

Ammar, M., Bahloul, N., Amri, O., Omri, R., Ghozzi, H., Kammoun, S., Zeghal, K., & Ben Mahmoud, L. (2022). Oxidative stress in patients with asthma and its relation to uncontrolled asthma. *Journal of Clinical Laboratory Analysis*, *36*(5). https://doi.org/10.1002/jcla.24345

Awasthi, A., Murugaiyan, G., & Kuchroo, V. K. (2008). Interplay between effector Th17 and regulatory T cells. In *Journal of Clinical Immunology* (Vol. 28, Issue 6, pp. 660–670). https://doi.org/10.1007/s10875-008-9239-7

Barlow, J. L., Peel, S., Fox, J., Panova, V., Hardman, C. S., Camelo, A., Bucks, C., Wu, X., Kane, C. M., Neill, D. R., Flynn, R. J., Sayers, I., Hall, I. P., & McKenzie, A. N. J. (2013). IL-33 is more potent than IL-25 in provoking IL-13-producing nuocytes (type 2 innate lymphoid cells) and airway contraction. *Journal of Allergy and Clinical Immunology*, *132*(4), 933–941. https://doi.org/10.1016/j.jaci.2013.05.012

Barnes, P. J. (2017). Cellular and molecular mechanisms of asthma and COPD. In *Clinical Science* (Vol. 131, Issue 13, pp. 1541–1558). Portland Press Ltd. https://doi.org/10.1042/CS20160487

Bayarri, M. A., Milara, J., Estornut, C., & Cortijo, J. (2021). Nitric Oxide System and Bronchial Epithelium: More Than a Barrier. In *Frontiers in Physiology* (Vol. 12). Frontiers Media S.A. https://doi.org/10.3389/fphys.2021.687381

Boxall, C. B., Holgate, S. T., & Davies, D. E. (2006). The contribution of transforming growth factor-β and epidermal growth factor signalling to airway remodelling in chronic asthma. In *European Respiratory Journal* (Vol. 27, Issue 1, pp. 208–229). https://doi.org/10.1183/09031936.06.00130004

Brandt, E. B., Kovacic, M. B., Lee, G. B., Gibson, A. M., Acciani, T. H., Le Cras, T. D., Ryan, P. H., Budelsky, A. L., & Khurana Hershey, G. K. (2013). Diesel exhaust particle induction of IL-17A contributes to severe asthma. *Journal of Allergy and Clinical Immunology*, *132*(5), 1194-1204.e2. https://doi.org/10.1016/j.jaci.2013.06.048

Brightling, C., Berry, M., & Amrani, Y. (2008). Targeting TNF-α: A novel therapeutic approach for asthma. In *Journal of Allergy and Clinical Immunology* (Vol. 121, Issue 1, pp. 5–10). https://doi.org/10.1016/j.jaci.2007.10.028

Burchill, M. A., Yang, J., Vogtenhuber, C., Blazar, B. R., & Farrar, M. A. (2007). IL-2 Receptor β-Dependent STAT5 Activation Is Required for the Development of Foxp3+ Regulatory T Cells. *The Journal of Immunology*, *178*(1), 280–290. https://doi.org/10.4049/jimmunol.178.1.280

Cazzola, M., & Polosa, R. (2006). Anti-TNF-α and Th1 cytokine-directed therapies for the treatment of asthma. *Current Opinion in Allergy & Clinical Immunology*, *6*(1), 43–50. https://doi.org/10.1097/01.all.0000199798.10047.74

Cevhertas, L., Ogulur, I., Maurer, D. J., Burla, D., Ding, M., Jansen, K., Koch, J., Liu, C., Ma, S., Mitamura, Y., Peng, Y., Radzikowska, U., Rinaldi, A. O., Satitsuksanoa, P., Globinska, A., van de Veen, W., Sokolowska, M., Baerenfaller, K., Gao, Y. dong, … Akdis, C. A. (2020). Advances and recent developments in asthma in 2020. In *Allergy: European Journal of Allergy and Clinical Immunology* (Vol. 75, Issue 12, pp. 3124–3146). Blackwell Publishing Ltd. https://doi.org/10.1111/all.14607

Chan, T. K., Tan, W. S. D., Peh, H. Y., & Wong, W. S. F. (2017). Aeroallergens Induce Reactive Oxygen Species Production and DNA Damage and Dampen Antioxidant Responses in Bronchial Epithelial Cells. *The Journal of Immunology*, *199*(1), 39–47. https://doi.org/10.4049/jimmunol.1600657

Chen, E., & Miller, G. E. (2007). Stress and inflammation in exacerbations of asthma. *Brain, Behavior, and Immunity*, *21*(8), 993–999. https://doi.org/10.1016/j.bbi.2007.03.009

Chen, G., & Khalil, N. (2006). TGF-β1 increases proliferation of airway smooth muscle cells by phosphorylation of map kinases. *Respiratory Research*, *7*(1), 2. https://doi.org/10.1186/1465-9921-7-2

Chetta, A., Zanini, A., Foresi, A., D’Ippolito, R., Tipa, A., Castagnaro, A., Baraldo, S., Neri, M., Saetta, M., & Olivieri, D. (2005). Vascular endothelial growth factor up-regulation and bronchial wall remodelling in asthma. *Clinical and Experimental Allergy*, *35*(11), 1437–1442. https://doi.org/10.1111/j.1365-2222.2005.02360.x

Chiba, Y., Nakazawa, S., Todoroki, M., Shinozaki, K., Sakai, H., & Misawa, M. (2009). Interleukin-13 augments bronchial smooth muscle contractility with an Up-regulation of RhoA protein. *American Journal of Respiratory Cell and Molecular Biology*, *40*(2), 159–167. https://doi.org/10.1165/rcmb.2008-0162OC

Chung, F. (2001). Anti-inflammatory cytokines in asthma and allergy: Interleukin-10, interleukin-12, interferon-γ. In *Mediators of Inflammation* (Vol. 10, Issue 2, pp. 51–59). https://doi.org/10.1080/09629350120054518

Coomes, S. M., Kannan, Y., Pelly, V. S., Entwistle, L. J., Guidi, R., Perez-Lloret, J., Nikolov, N., Müller, W., & Wilson, M. S. (2017). CD4 + Th2 cells are directly regulated by IL-10 during allergic airway inflammation. *Mucosal Immunology*, *10*(1), 150–161. https://doi.org/10.1038/mi.2016.47

Doherty, T., & Broide, D. (2007). Cytokines and growth factors in airway remodeling in asthma. In *Current Opinion in Immunology* (Vol. 19, Issue 6, pp. 676–680). https://doi.org/10.1016/j.coi.2007.07.017

Donnelly, L. E., & Barnes, P. J. (2002). Expression and Regulation of Inducible Nitric Oxide Synthase from Human Primary Airway Epithelial Cells. *American Journal of Respiratory Cell and Molecular Biology*, *26*(1), 144–151. https://doi.org/10.1165/ajrcmb.26.1.4477

Du, L., & Roberts, J. D. (2019). Transforming growth factor-downregulates sGC subunit expression in pulmonary artery smooth muscle cells via MEK and ERK signaling. *Am J Physiol Lung Cell Mol Physiol*, *316*, 20–34. https://doi.org/10.1152/ajplung.00319.2018.-TGF

Duchesne, M., Okoye, I., & Lacy, P. (2022). Epithelial cell alarmin cytokines: Frontline mediators of the asthma inflammatory response. In *Frontiers in Immunology* (Vol. 13). Frontiers Media S.A. https://doi.org/10.3389/fimmu.2022.975914

Elser, B., Lohoff, M., Kock, S., Giaisi, M., Kirchhoff, S., Krammer, P. H., & Li-Weber, M. (2002). Transcription factors such as c-maf (Ho 2 Institute for Medical Microbiology. In *German Cancer Research Center (DKFZ)* (Vol. 17). Zheng and Flavell.

Erle, D. J., & Sheppard, D. (2014). The cell biology of asthma. In *Journal of Cell Biology* (Vol. 205, Issue 5, pp. 621–631). Rockefeller University Press. https://doi.org/10.1083/jcb.201401050

Flesch, I. E. A., Hess, J. H., Huang, S., Aguet, M., Rothe, J., Bluethmann, H., & Kaufmann, S. H. E. (1995). Early interleukin 12 production by macrophages in response to mycobacterial infection depends on interferon γ and tumor necrosis factor α. *Journal of Experimental Medicine*, *181*(5), 1615–1621. https://doi.org/10.1084/jem.181.5.1615

Fujisawa, T., Chang, M. M. J., Velichko, S., Thai, P., Hung, L. Y., Huang, F., Phuong, N., Chen, Y., & Wu, R. (2011). NF-κB mediates IL-1β- and IL-17A-induced MUC5B expression in airway epithelial cells. *American Journal of Respiratory Cell and Molecular Biology*, *45*(2), 246–252. https://doi.org/10.1165/rcmb.2009-0313OC

Ganster, R. W., Taylor, B. S., Shao, L., & Geller, D. A. (2001). Complex regulation of human inducible nitric oxide synthase gene transcription by Stat 1 and NF-κB. *Proceedings of the National Academy of Sciences*, *98*(15), 8638–8643. https://doi.org/10.1073/pnas.151239498

Gauthier, M., Chakraborty, K., Oriss, T. B., Raundhal, M., Das, S., Chen, J., Huff, R., Sinha, A., Fajt, M., Ray, P., Wenzel, S. E., & Ray, A. (2017). Severe asthma in humans and mouse model suggests a CXCL10 signature underlies corticosteroid-resistant Th1 bias. *JCI Insight*, *2*(13). https://doi.org/10.1172/jci.insight.94580

Girodet, P.-O., Nguyen, D., Mancini, J. D., Hundal, M., Zhou, X., Israel, E., & Cernadas, M. (2016). Alternative Macrophage Activation Is Increased in Asthma. *American Journal of Respiratory Cell and Molecular Biology*, *55*(4), 467–475. https://doi.org/10.1165/rcmb.2015-0295OC

Guan, Y., Zhu, J. ping, Shen, J., Jia, Y. liang, Jin, Y. chao, Dong, X. wei, & Xie, Q. min. (2018). Salvianolic acid B improves airway hyperresponsiveness by inhibiting MUC5AC overproduction associated with Erk1/2/P38 signaling. *European Journal of Pharmacology*, *824*, 30–39. https://doi.org/10.1016/j.ejphar.2018.01.050

Ha, E. V. S., & Rogers, D. F. (2016). Novel Therapies to Inhibit Mucus Synthesis and Secretion in Airway Hypersecretory Diseases. In *Pharmacology* (Vol. 97, Issues 1–2, pp. 84–100). S. Karger AG. https://doi.org/10.1159/000442794

Habibovic, A., Hristova, M., Heppner, D. E., Danyal, K., Ather, J. L., Janssen-Heininger, Y. M. W., Irvin, C. G., Poynter, M. E., Lundblad, L. K., Dixon, A. E., Geiszt, M., & van der Vliet, A. (2016). DUOX1 mediates persistent epithelial EGFR activation, mucous cell metaplasia, and airway remodeling during allergic asthma. *JCI Insight*, *1*(18). https://doi.org/10.1172/jci.insight.88811

Hammad, H., Chieppa, M., Perros, F., Willart, M. A., Germain, R. N., & Lambrecht, B. N. (2009). House dust mite allergen induces asthma via Toll-like receptor 4 triggering of airway structural cells. *Nature Medicine*, *15*(4), 410–416. https://doi.org/10.1038/nm.1946

Han, Z., Junxu, A., & Zhong, N. (2003). Expression of matrix metalloproteinases MMP-9 within the airways in asthma. *Respiratory Medicine*, *97*(5), 563–567. https://doi.org/10.1053/rmed.2001.1162

Hoshino, M., Takahashi, M., & Aoike, N. (2001). Expression of vascular endothelial growth factor, basic fibroblast growth factor, and angiogenin immunoreactivity in asthmatic airways and its relationship to angiogenesis. *Journal of Allergy and Clinical Immunology*, *107*(2), 295–301. https://doi.org/10.1067/mai.2001.111928

Hough, K. P., Curtiss, M. L., Blain, T. J., Liu, R. M., Trevor, J., Deshane, J. S., & Thannickal, V. J. (2020). Airway Remodeling in Asthma. In *Frontiers in Medicine* (Vol. 7). Frontiers Media S.A. https://doi.org/10.3389/fmed.2020.00191

Hsia, B. J., Whitehead, G. S., Thomas, S. Y., Nakano, K., Gowdy, K. M., Aloor, J. J., Nakano, H., & Cook, D. N. (2015). Trif-dependent induction of Th17 immunity by lung dendritic cells. *Mucosal Immunology*, *8*(1), 186–197. https://doi.org/10.1038/mi.2014.56

Hsieh, A., Assadinia, N., & Hackett, T. L. (2023). Airway remodeling heterogeneity in asthma and its relationship to disease outcomes. In *Frontiers in Physiology* (Vol. 14). Frontiers Media S.A. https://doi.org/10.3389/fphys.2023.1113100

Hynes, G. M., & Hinks, T. S. C. (2020). The role of interleukin-17 in asthma: A protective response? In *ERJ Open Research* (Vol. 6, Issue 2). European Respiratory Society. https://doi.org/10.1183/23120541.00364-2019

Hyun, J. K., Park, Y. D., Uk, Y. M., Kim, J. H., Ju, H. J., Lee, J. G., Yun, S. B., & Yoon, J. H. (2008). The role of Nox4 in oxidative stress-induced MUC5AC overexpression in human airway epithelial cells. *American Journal of Respiratory Cell and Molecular Biology*, *39*(5), 598–609. https://doi.org/10.1165/rcmb.2007-0262OC

Jia, H., Sodhi, C. P., Yamaguchi, Y., Lu, P., Martin, L. Y., Good, M., Zhou, Q., Sung, J., Fulton, W. B., Nino, D. F., Prindle, T., Ozolek, J. A., & Hackam, D. J. (2016). Pulmonary Epithelial TLR4 Activation Leads to Lung Injury in Neonatal Necrotizing Enterocolitis. *The Journal of Immunology*, *197*(3), 859–871. https://doi.org/10.4049/jimmunol.1600618

Jiang, Z., & Zhu, L. (2016). Update on the role of alternatively activated macrophages in asthma. *Journal of Asthma and Allergy*, *9*, 101–107. https://doi.org/10.2147/JAA.S104508

Joseph, C., & Tatler, A. L. (2022). Pathobiology of Airway Remodeling in Asthma: The Emerging Role of Integrins. In *Journal of Asthma and Allergy* (Vol. 15, pp. 595–610). Dove Medical Press Ltd. https://doi.org/10.2147/JAA.S267222

Kaiko, G. E., Horvat, J. C., Beagley, K. W., & Hansbro, P. M. (2008). Immunological decision-making: How does the immune system decide to mount a helper T-cell response? In *Immunology* (Vol. 123, Issue 3, pp. 326–338). https://doi.org/10.1111/j.1365-2567.2007.02719.x

Kariyawasam, H. H., & Robinson, D. S. (2007). The role of eosinophils in airway tissue remodelling in asthma. In *Current Opinion in Immunology* (Vol. 19, Issue 6, pp. 681–686). https://doi.org/10.1016/j.coi.2007.07.021

Kim, B.-J., & Jones, H. P. (2010). Epinephrine-primed murine bone marrow-derived dendritic cells facilitate production of IL-17A and IL-4 but not IFN-γ by CD4+ T cells. *Brain, Behavior, and Immunity*, *24*(7), 1126–1136. https://doi.org/10.1016/j.bbi.2010.05.003

Kim, S. H., Hong, J. H., & Lee, Y. C. (2014). Oleanolic acid suppresses ovalbumin-induced airway inflammation and Th2-mediated allergic asthma by modulating the transcription factors T-bet, GATA-3, RORγt and Foxp3 in asthmatic mice. *International Immunopharmacology*, *18*(2), 311–324. https://doi.org/10.1016/j.intimp.2013.12.009

Kim, Y.-S., Hong, S.-W., Choi, J.-P., Shin, T.-S., Moon, H.-G., Choi, E.-J., Jeon, S. G., Oh, S.-Y., Gho, Y. S., Zhu, Z., & Kim, Y.-K. (2009). Vascular Endothelial Growth Factor Is a Key Mediator in the Development of T Cell Priming and Its Polarization to Type 1 and Type 17 T Helper Cells in the Airways. *The Journal of Immunology*, *183*(8), 5113–5120. https://doi.org/10.4049/jimmunol.0901566

Koff, J. L., Shao, M. X. G., Ueki, I. F., & Nadel, J. A. (2008). Multiple TLRs activate EGFR via a signaling cascade to produce innate immune responses in airway epithelium Koff JL, Shao MX, Ueki IF, Nadel JA. Multiple TLRs acti-vate EGFR via a signaling cascade to produce innate immune responses in airway epithelium. *Am J Physiol Lung Cell Mol Physiol*, *294*, 1068–1075. https://doi.org/10.1152/ajplung.00025.2008.-Toll-like

Komlósi, Z. I., van de Veen, W., Kovács, N., Szűcs, G., Sokolowska, M., O’Mahony, L., Akdis, M., & Akdis, C. A. (2022). Cellular and molecular mechanisms of allergic asthma. In *Molecular Aspects of Medicine* (Vol. 85). Elsevier Ltd. https://doi.org/10.1016/j.mam.2021.100995

Kouzaki, H., O’Grady, S. M., Lawrence, C. B., & Kita, H. (2009). Proteases Induce Production of Thymic Stromal Lymphopoietin by Airway Epithelial Cells through Protease-Activated Receptor-2. *The Journal of Immunology*, *183*(2), 1427–1434. https://doi.org/10.4049/jimmunol.0900904

Kristof, A. S., Marks-Konczalik, J., & Moss, J. (2001). Mitogen-activated Protein Kinases Mediate Activator Protein-1-dependent Human Inducible Nitric-oxide Synthase Promoter Activation. *Journal of Biological Chemistry*, *276*(11), 8445–8452. https://doi.org/10.1074/jbc.M009563200

Kudo, M., Ishigatsubo, Y., & Aoki, I. (2013). Pathology of asthma. In *Frontiers in Microbiology* (Vol. 4, Issue SEP). Frontiers Research Foundation. https://doi.org/10.3389/fmicb.2013.00263

Lambrecht, B. N., Hammad, H., & Fahy, J. V. (2019). The Cytokines of Asthma. In *Immunity* (Vol. 50, Issue 4, pp. 975–991). Cell Press. https://doi.org/10.1016/j.immuni.2019.03.018

Le, A. V., Cho, J. Y., Miller, M., McElwain, S., Golgotiu, K., & Broide, D. H. (2007). Inhibition of Allergen-Induced Airway Remodeling in Smad 3-Deficient Mice. *The Journal of Immunology*, *178*(11), 7310–7316. https://doi.org/10.4049/jimmunol.178.11.7310

Lee, J.-W., Chun, W., Lee, H. J., Min, J.-H., Kim, S.-M., Seo, J.-Y., Ahn, K.-S., & Oh, S.-R. (2021). The Role of Macrophages in the Development of Acute and Chronic Inflammatory Lung Diseases. *Cells*, *10*(4), 897. https://doi.org/10.3390/cells10040897

Lee, K. S., Park, S. J., Kim, S. R., Min, K. H., Lee, K. Y., Choe, Y. H., Hong, S. H., Lee, Y. R., Kim, J. S., Hong, S. J., & Lee, Y. C. (2008). Inhibition of VEGF blocks TGF-β 1 production through a PI3K/Akt signalling pathway. *European Respiratory Journal*, *31*(3), 523–531. https://doi.org/10.1183/09031936.00125007

Liu, K., Hua, S., & Song, L. (2022). PM2.5 Exposure and Asthma Development: The Key Role of Oxidative Stress. In *Oxidative Medicine and Cellular Longevity* (Vol. 2022). Hindawi Limited. https://doi.org/10.1155/2022/3618806

Liu, Y., Jiang, B. J., Zhao, R. Z., & Ji, H. L. (2016). Epithelial sodium channels in pulmonary epithelial progenitor and stem cells. In *International Journal of Biological Sciences* (Vol. 12, Issue 9, pp. 1150–1154). Ivyspring International Publisher. https://doi.org/10.7150/ijbs.15747

London, S., Koziol‐White, C., Guo, C., Panettieri, R., & Gow, A. (2018). Downregulation of Guanylate Cyclase Enzyme in Human Asthma model to Investigate NO‐sGc‐ cGMP as a Therapeutic Pathway in Asthma. *The FASEB Journal*, *32*(S1). https://doi.org/10.1096/fasebj.2018.32.1_supplement.840.11

Lora, J. M., Zhang, D. M., Liao, S. M., Burwell, T., King, A. M., Barker, P. A., Singh, L., Keaveney, M., Morgenstern, J., Gutiérrez-Ramos, J. C., Coyle, A. J., & Fraser, C. C. (2005). Tumor necrosis factor-α triggers mucus production in airway epithelium through an IκB kinase β-dependent mechanism. *Journal of Biological Chemistry*, *280*(43), 36510–36517. https://doi.org/10.1074/jbc.M507977200

Luckheeram, R. V., Zhou, R., Verma, A. D., & Xia, B. (2012). CD4 +T cells: Differentiation and functions. In *Clinical and Developmental Immunology* (Vol. 2012). https://doi.org/10.1155/2012/925135

Ma, Y., Zhang, J. X., Liu, Y. N., Ge, A., Gu, H., Zha, W. J., Zeng, X. N., & Huang, M. (2016). Caffeic acid phenethyl ester alleviates asthma by regulating the airway microenvironment via the ROS-responsive MAPK/Akt pathway. In *Free Radical Biology and Medicine* (Vol. 101, pp. 163–175). Elsevier Inc. https://doi.org/10.1016/j.freeradbiomed.2016.09.012

Magee, C. N., Boenisch, O., & Najafian, N. (2012). The role of costimulatory molecules in directing the functional differentiation of alloreactive T helper cells. In *American Journal of Transplantation* (Vol. 12, Issue 10, pp. 2588–2600). https://doi.org/10.1111/j.1600-6143.2012.04180.x

Mattila, J. T., & Thomas, A. C. (2014). Nitric Oxide Synthase: Non-Canonical Expression Patterns. *Frontiers in Immunology*, *5*. https://doi.org/10.3389/fimmu.2014.00478

McGeachy, M. J., Chen, Y., Tato, C. M., Laurence, A., Joyce-Shaikh, B., Blumenschein, W. M., McClanahan, T. K., O’Shea, J. J., & Cua, D. J. (2009). The interleukin 23 receptor is essential for the terminal differentiation of interleukin 17-producing effector T helper cells in vivo. *Nature Immunology*, *10*(3), 314–324. https://doi.org/10.1038/ni.1698

Mishra, V., Banga, J., & Silveyra, P. (2018). Oxidative stress and cellular pathways of asthma and inflammation: Therapeutic strategies and pharmacological targets. In *Pharmacology and Therapeutics* (Vol. 181, pp. 169–182). Elsevier Inc. https://doi.org/10.1016/j.pharmthera.2017.08.011

Miyasaka, T., Dobashi-Okuyama, K., Takahashi, T., Takayanagi, M., & Ohno, I. (2018). The interplay between neuroendocrine activity and psychological stress-induced exacerbation of allergic asthma. *Allergology International*, *67*(1), 32–42. https://doi.org/10.1016/j.alit.2017.04.013

Moffatt, M. F., Gut, I. G., Demenais, F., Strachan, D. P., Bouzigon, E., Heath, S., von Mutius, E., Farrall, M., Lathrop, M., & Cookson, W. O. C. M. (2010). A Large-Scale, Consortium-Based Genomewide Association Study of Asthma. *New England Journal of Medicine*, *363*(13), 1211–1221. https://doi.org/10.1056/NEJMoa0906312

Mott, J. D., & Werb, Z. (2004). Regulation of matrix biology by matrix metalloproteinases. In *Current Opinion in Cell Biology* (Vol. 16, Issue 5, pp. 558–564). https://doi.org/10.1016/j.ceb.2004.07.010

Nadeem, A., Alharbi, N. O., Vliagoftis, H., Tyagi, M., Ahmad, S. F., & Sayed-Ahmed, M. M. (2015). Proteinase activated receptor-2-mediated dual oxidase-2 up-regulation is involved in enhanced airway reactivity and inflammation in a mouse model of allergic asthma. *Immunology*, *145*(3), 391–403. https://doi.org/10.1111/imm.12453

Newcomb, D. C., & Peebles, R. S. (2013). Th17-mediated inflammation in asthma. In *Current Opinion in Immunology* (Vol. 25, Issue 6, pp. 755–760). https://doi.org/10.1016/j.coi.2013.08.002

Nie, H. G., Chen, L., Han, D. Y., Li, J., Song, W. F., Wei, S. P., Fang, X. H., Gu, X., Matalon, S., & Ji, H. L. (2009). Regulation of epithelial sodium channels by cGMP/PKGII. *Journal of Physiology*, *587*(11), 2663–2676. https://doi.org/10.1113/jphysiol.2009.170324

O’Sullivan, M. J., Phung, T. K. N., & Park, J. A. (2020). Bronchoconstriction: A potential missing link in airway remodelling: Bronchoconstriction causes remodeling. In *Open Biology* (Vol. 10, Issue 12). Royal Society Publishing. https://doi.org/10.1098/rsob.200254

Ojiaku, C. A., Yoo, E. J., & Panettieri, R. A. (2017). Transforming growth factor β1 function in airway remodeling and hyperresponsiveness: The missing link? In *American Journal of Respiratory Cell and Molecular Biology* (Vol. 56, Issue 4, pp. 432–442). American Thoracic Society. https://doi.org/10.1165/rcmb.2016-0307TR

Okada, S., Kita, H., George, T. J., Gleich, G. J., & Leiferman, K. M. (1997). Migration of Eosinophils through Basement Membrane Components in Vitro: Role of Matrix Metalloproteinase-9. *American Journal of Respiratory Cell and Molecular Biology*, *17*(4), 519–528. https://doi.org/10.1165/ajrcmb.17.4.2877

Okuyama, K., Ide, S., Sakurada, S., Sasaki, K., Sora, I., Tamura, G., Ohkawara, Y., Takayanagi, M., & Ohno, I. (2012). Μ-Opioid Receptor-Mediated Alterations of Allergen-Induced Immune Responses of Bronchial Lymph Node Cells in a Murine Model of Stress Asthma. *Allergology International*, *61*(2), 245–258. https://doi.org/10.2332/allergolint.11-OA-0304

Okuyama, K., Wada, K., Sakurada, S., Mizoguchi, H., Komatsu, H., Sora, I., Tamura, G., Ohkawara, Y., Takayanagi, M., & Ohno, I. (2010). The involvement of μ-opioid receptors in the central nervous system in the worsening of allergic airway inflammation by psychological stress in mice. *International Archives of Allergy and Immunology*, *152*(4), 342–352. https://doi.org/10.1159/000288287

Papapetropoulos, A., Simoes, D. C. M., Xanthou, G., Roussos, C., & Gratziou, C. (2006). Soluble guanylyl cyclase expression is reduced in allergic asthma. *American Journal of Physiology-Lung Cellular and Molecular Physiology*, *290*(1), L179–L184. https://doi.org/10.1152/ajplung.00330.2005

Park, J. A., Drazen, J. M., & Tschumperlin, D. J. (2010). The chitinase-like protein YKL-40 is secreted by airway epithelial cells at base line and in response to compressive mechanical stress. *Journal of Biological Chemistry*, *285*(39), 29817–29825. https://doi.org/10.1074/jbc.M110.103416

Pelaia, G., Vatrella, A., Busceti, M. T., Gallelli, L., Calabrese, C., Terracciano, R., & Maselli, R. (2015). Cellular mechanisms underlying eosinophilic and neutrophilic airway inflammation in asthma. In *Mediators of Inflammation* (Vol. 2015). Hindawi Limited. https://doi.org/10.1155/2015/879783

Piggott, D. A., Eisenbarth, S. C., Xu, L., Constant, S. L., Huleatt, J. W., Herrick, C. A., & Bottomly, K. (2005). MyD88-dependent induction of allergic Th2 responses to intranasal antigen. *Journal of Clinical Investigation*, *115*(2), 459–467. https://doi.org/10.1172/JCI200522462

Price, M. E., & Sisson, J. H. (2019). Redox regulation of motile cilia in airway disease. In *Redox Biology* (Vol. 27). Elsevier B.V. https://doi.org/10.1016/j.redox.2019.101146

Raundhal, M., Morse, C., Khare, A., Oriss, T. B., Milosevic, J., Trudeau, J., Huff, R., Pilewski, J., Holguin, F., Kolls, J., Wenzel, S., Ray, P., & Ray, A. (2015). High IFN-γ and low SLPI mark severe asthma in mice and humans. *Journal of Clinical Investigation*, *125*(8), 3037–3050. https://doi.org/10.1172/JCI80911

Rayees, S., Din, I., Singh, G., & Malik, F. A. (2020). Chronic Lung Diseases: Pathophysiology and Therapeutics. In *Chronic Lung Diseases: Pathophysiology and Therapeutics*. Springer Singapore. https://doi.org/10.1007/978-981-15-3734-9

Roos, A. B., Mori, M., Grönneberg, R., Österlund, C., Claesson, H.-E., Wahlström, J., Grunewald, J., Eklund, A., Erjefält, J. S., Lundberg, J. O., & Nord, M. (2014). Elevated Exhaled Nitric Oxide in Allergen-Provoked Asthma Is Associated with Airway Epithelial iNOS. *PLoS ONE*, *9*(2), e90018. https://doi.org/10.1371/journal.pone.0090018

Rosenkranz, M. A., Esnault, S., Gresham, L., Davidson, R. J., Christian, B. T., Jarjour, N. N., & Busse, W. W. (2022). Role of amygdala in stress-induced upregulation of airway IL-1 signaling in asthma. *Biological Psychology*, *167*, 1–26. https://doi.org/10.1016/j.biopsycho.2021.108226

Ross, E. A., Devitt, A., & Johnson, J. R. (2021). Macrophages: The Good, the Bad, and the Gluttony. *Frontiers in Immunology*, *12*(August), 1–22. https://doi.org/10.3389/fimmu.2021.708186

Royce, S. G., Cheng, V., Samuel, C. S., & Tang, M. L. K. (2012). The regulation of fibrosis in airway remodeling in asthma. In *Molecular and Cellular Endocrinology* (Vol. 351, Issue 2, pp. 167–175). https://doi.org/10.1016/j.mce.2012.01.007

Ryu, J. H., Yoo, J. Y., Kim, M. J., Hwang, S. G., Ahn, K. C., Ryu, J. C., Choi, M. K., Joo, J. H., Kim, C. H., Lee, S. N., Lee, W. J., Kim, J., Shin, D. M., Kweon, M. N., Bae, Y. S., & Yoon, J. H. (2013). Distinct TLR-mediated pathways regulate house dust mite-induced allergic disease in the upper and lower airways. *Journal of Allergy and Clinical Immunology*, *131*(2), 549–561. https://doi.org/10.1016/j.jaci.2012.07.050

Sagara, H., Okada, T., Okumura, K., Ogawa, H., Ra, C., Fukuda, T., & Nakao, A. (2002). Activation of TGF-β/Smad2 signaling is associated with airway remodeling in asthma. *Journal of Allergy and Clinical Immunology*, *110*(2), 249–254. https://doi.org/10.1067/mai.2002.126078

Saradna, A., Do, D. C., Kumar, S., Fu, Q.-L., & Gao, P. (2018). Macrophage polarization and allergic asthma. *Translational Research*, *191*, 1–14. https://doi.org/10.1016/j.trsl.2017.09.002

Savin, I. A., Zenkova, M. A., & Sen’kova, A. V. (2023). Bronchial Asthma, Airway Remodeling and Lung Fibrosis as Successive Steps of One Process. In *International Journal of Molecular Sciences* (Vol. 24, Issue 22). Multidisciplinary Digital Publishing Institute (MDPI). https://doi.org/10.3390/ijms242216042

Schülke, S. (2018). Induction of interleukin-10 producing dendritic cells as a tool to suppress allergen-specific T helper 2 responses. *Frontiers in Immunology*, *9*(MAR). https://doi.org/10.3389/fimmu.2018.00455

Siew, L. Q. C., Wu, S. ‐Y., Ying, S., & Corrigan, C. J. (2017). Cigarette smoking increases bronchial mucosal IL‐17A expression in asthmatics, which acts in concert with environmental aeroallergens to engender neutrophilic inflammation. *Clinical & Experimental Allergy*, *47*(6), 740–750. https://doi.org/10.1111/cea.12907

Simcock, D. E., Kanabar, V., Clarke, G. W., Mahn, K., Karner, C., O’Connor, B. J., Lee, T. H., & Hirst, S. J. (2008). Induction of angiogenesis by airway smooth muscle from patients with asthma. *American Journal of Respiratory and Critical Care Medicine*, *178*(5), 460–468. https://doi.org/10.1164/rccm.200707-1046OC

Sturrock, A., Huecksteadt, T. P., Norman, K., Sanders, K., Murphy, T. M., Chitano, P., Wilson, K., Hoidal, J. R., & Kennedy, T. P. (2007). Nox4 mediates TGF-1-induced retinoblastoma protein phosphorylation, proliferation, and hypertrophy in human airway smooth muscle cells. *Am J Physiol Lung Cell Mol Physiol*, *292*, 1543–1555. https://doi.org/10.1152/ajplung.00430.2006.-Trans

Suraya, R., Nagano, T., Katsurada, M., Sekiya, R., Kobayashi, K., & Nishimura, Y. (2021). Molecular mechanism of asthma and its novel molecular target therapeutic agent. In *Respiratory Investigation* (Vol. 59, Issue 3, pp. 291–301). Elsevier B.V. https://doi.org/10.1016/j.resinv.2020.12.007

Sze, E., Bhalla, A., & Nair, P. (2020). Mechanisms and therapeutic strategies for non-T2 asthma. In *Allergy: European Journal of Allergy and Clinical Immunology* (Vol. 75, Issue 2, pp. 311–325). Blackwell Publishing Ltd. https://doi.org/10.1111/all.13985

Tagaya, E., & Tamaoki, J. (2007). Mechanisms of Airway Remodeling in Asthma. In *Allergology International* (Vol. 56). www.jsaweb.jp!

Teixeira, L. K., Fonseca, B. P., Barboza, B. A., & Viola, J. P. (2005). The role of interferon-γ γ γ γ γ on immune and allergic responses. In *Mem Inst Oswaldo Cruz, Rio de Janeiro* (Vol. 100).

Tschumperlin, D. J., Shively, J. D., Swartz, M. A., Silverman, E. S., Haley, K. J., Raab, G., & Drazen, J. M. (2002). Bronchial epithelial compression regulates MAP kinase signaling and HB-EGF-like growth factor expression. *American Journal of Physiology - Lung Cellular and Molecular Physiology*, *282*(5 26-5). https://doi.org/10.1152/ajplung.00270.2001

Türkeli, A., Yilmaz, Ö., Karaman, M., Kanik, E., Firinci, F., İnan, S., & Yüksel, H. (2021). Anti‑VEGF treatment suppresses remodeling factors and restores epithelial barrier function through the E‑cadherin/β‑catenin signaling axis in experimental asthma models. *Experimental and Therapeutic Medicine*, *22*(1). https://doi.org/10.3892/etm.2021.10121

van der Vliet, A., Danyal, K., & Heppner, D. E. (2018). Dual oxidase: a novel therapeutic target in allergic disease. In *British Journal of Pharmacology* (Vol. 175, Issue 9, pp. 1401–1418). John Wiley and Sons Inc. https://doi.org/10.1111/bph.14158

VIGNOLA, A. M., CHANEZ, P., CHIAPPARA, G., MERENDINO, A., PACE, E., RIZZO, A., la ROCCA, A. M., BELLIA, V., BONSIGNORE, G., & BOUSQUET, J. (1997). Transforming Growth Factor- β Expression in Mucosal Biopsies in Asthma and Chronic Bronchitis. *American Journal of Respiratory and Critical Care Medicine*, *156*(2), 591–599. https://doi.org/10.1164/ajrccm.156.2.9609066

Vroman, H., van den Blink, B., & Kool, M. (2015). Mode of dendritic cell activation: The decisive hand in Th2/Th17 cell differentiation. Implications in asthma severity? In *Immunobiology* (Vol. 220, Issue 2, pp. 254–261). Elsevier GmbH. https://doi.org/10.1016/j.imbio.2014.09.016

Walker, L. S. K. (2013). Treg and CTLA-4: Two intertwining pathways to immune tolerance. In *Journal of Autoimmunity* (Vol. 45, pp. 49–57). https://doi.org/10.1016/j.jaut.2013.06.006

Wan, W. Y. H., Hollins, F., Haste, L., Woodman, L., Hirst, R. A., Bolton, S., Gomez, E., Sutcliffe, A., Desai, D., Chachi, L., Mistry, V., Szyndralewiez, C., Wardlaw, A., Saunders, R., O’Callaghan, C., Andrew, P. W., & Brightling, C. E. (2016). NADPH Oxidase-4 Overexpression Is Associated with Epithelial Ciliary Dysfunction in Neutrophilic Asthma. *Chest*, *149*(6), 1445–1459. https://doi.org/10.1016/j.chest.2016.01.024

Yoshida, K., Takabayashi, T., Kaneko, A., Takiyama, M., Sakashita, M., Imoto, Y., Kato, Y., Narita, N., & Fujieda, S. (2021). Baicalin suppresses type 2 immunity through breaking off the interplay between mast cell and airway epithelial cell. *Journal of Ethnopharmacology*, *267*. https://doi.org/10.1016/j.jep.2020.113492

Zakeri, A., & Russo, M. (2018). Dual role of toll-like receptors in human and experimental asthma models. In *Frontiers in Immunology* (Vol. 9, Issue MAY). Frontiers Media S.A. https://doi.org/10.3389/fimmu.2018.01027

Zhang, X., Xu, Z., Wen, X., Huang, G., Nian, S., Li, L., Guo, X., Ye, Y., & Yuan, Q. (2022). The onset, development and pathogenesis of severe neutrophilic asthma. In *Immunology and Cell Biology* (Vol. 100, Issue 3, pp. 144–159). John Wiley and Sons Inc. https://doi.org/10.1111/imcb.12522

Zhao, S. tao, & Wang, C. zheng. (2018). Regulatory T cells and asthma. In *Journal of Zhejiang University: Science B* (Vol. 19, Issue 9, pp. 663–673). Zhejiang University Press. https://doi.org/10.1631/jzus.B1700346

Zhu, X., Cui, J., Yi, L., Qin, J., Tulake, W., Teng, F., Tang, W., Wei, Y., & Dong, J. (2020a). The Role of T Cells and Macrophages in Asthma Pathogenesis: A New Perspective on Mutual Crosstalk. *Mediators of Inflammation*, *2020*. https://doi.org/10.1155/2020/7835284

Zhu, X., Cui, J., Yi, L., Qin, J., Tulake, W., Teng, F., Tang, W., Wei, Y., & Dong, J. (2020b). The Role of T Cells and Macrophages in Asthma Pathogenesis: A New Perspective on Mutual Crosstalk. *Mediators of Inflammation*, *2020*(Figure 1). https://doi.org/10.1155/2020/7835284

Zuo, L., Lucas, K., Fortuna, C. A., Chuang, C. C., & Best, T. M. (2015). Molecular regulation of toll-like receptors in asthma and COPD. In *Frontiers in Physiology* (Vol. 6, Issue NOV). Frontiers Research Foundation. https://doi.org/10.3389/fphys.2015.00312
